# G-quadruplex located in the 5′UTR of the BAG-1 mRNA affects both its cap-dependent and cap-independent translation through global secondary structure maintenance

**DOI:** 10.1093/nar/gkz777

**Published:** 2019-09-04

**Authors:** Rachel Jodoin, Julie C Carrier, Nathalie Rivard, Martin Bisaillon, Jean-Pierre Perreault

**Affiliations:** 1 Département de Biochimie, Faculté de médecine et des sciences de la santé, Université de Sherbrooke, Sherbrooke, Québec J1E 4K8, Canada; 2 Service de Gastro-entérologie, Département de médecine, Faculté de médecine et des sciences de la santé, Université de Sherbrooke, Sherbrooke, Québec J1H 5N4, Canada; 3 Département d’Anatomie et de Biologie Cellulaire, Faculté de médecine et des sciences de la santé, Université de Sherbrooke, Sherbrooke, Québec J1E 4K8, Canada

## Abstract

The anti-apoptotic BAG-1 protein isoforms are known to be overexpressed in colorectal tumors and are considered to be potential therapeutic targets. The isoforms are derived from alternative translation initiations occuring at four in-frame start codons of a single mRNA transcript. Its 5′UTR also contains an internal ribosome entry site (IRES) regulating the cap-independent translation of the transcript. An RNA G-quadruplex (rG4) is located at the 5′end of the BAG-1 5′UTR, upstream of the known *cis*-regulatory elements. Herein, we observed that the expression of BAG-1 isoforms is post-transcriptionally regulated in colorectal cancer cells and tumors, and that stabilisation of the rG4 by small molecules ligands reduces the expression of endogenous BAG-1 isoforms. We demonstrated a critical role for the rG4 in the control of both cap-dependent and independent translation of the BAG-1 mRNA in colorectal cancer cells. Additionally, we found an upstream ORF that also represses BAG-1 mRNA translation. The structural probing of the complete 5′UTR showed that the rG4 acts as a steric block which controls the initiation of translation at each start codon of the transcript and also maintains the global 5′UTR secondary structure required for IRES-dependent translation.

## INTRODUCTION

The BAG-1 protein (Bcl2-associated athanogene 1) was initially identified as an interactor of the anti-apoptotic protein BCL-2 (1), and is known to be an inhibitor of the intrinsic apoptotic pathway (2,3). BAG-1 was further characterized as being a multifunctional protein. Among its functions, BAG-1 acts as a nucleotide exchange factor that modulates the activity of the chaperones Hsp70/Hsc70 (4,5). BAG-1 is also known to interact with a diverse array of partners including the retinoblastoma protein (pRb) (6); the oncogenic kinase Raf-1 (7); the transcription factor NFκB and several nuclear hormones and growth receptors such as the platelet-derived growth factor receptor (PDGF-R), the hepatocyte growth factor receptor (HGF-R) and the androgen receptor (8–11) to name a few. Overall, depending on the interactions with the various partners, BAG-1 integrates signals from multiple pathways hence modulating not only survival, but also gene transcription, cell proliferation, and growth (12).

**Figure 1.**
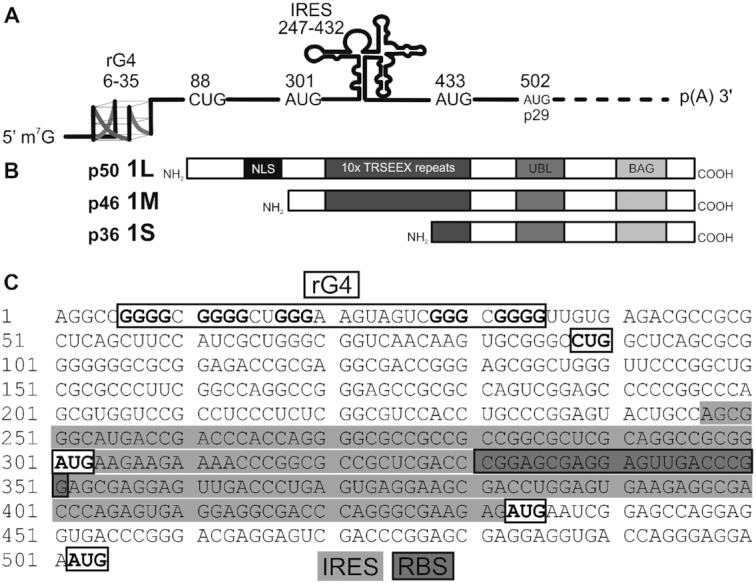
Scheme of the BAG-1 mRNA organization. (**A**)The BAG-1 mRNA presents many features in its 5′UTR: an rG4 secondary structure located at its 5′ end; four in-frame start codons with the first being a non-canonical CUG; and, an IRES secondary structure. (**B**) Translational initiation at the three principal alternative start codons results in the production of three protein isoforms (1L, Long, p50; 1M, Medium, p46; and, 1S, Short, p36) differing from each other by the size of their N-terminal extension. The rarely expressed, shortest BAG-1 isoform p29 is not represented. Its translation starts from the AUG located at position 502. (**C**) Nucleotide sequence of the complete BAG-1 5′UTR. The rG4 region, the start codons, the IRES region and the ribosome binding sites are highlighted.

The BAG-1 protein is expressed in three main isoforms: the long, BAG-1L (50 kDa); the medium, BAG-1M (46 kDa); and, the short, BAG-1S (36 kDa). There is also a fourth isoform, less abundant, BAG-1 p29 (29 kDa) (12,13). All BAG-1 protein isoforms are translated from the same mRNA transcript via two mechanisms. The first mechanism is called ‘leaky scanning’ and it consists of differential rates of translation initiation at either one of the four in-frame start codons present in the 501 nucleotides (nts) long 5′-untranslated region (5′UTR) of the mRNA (13,14), depending on the strength of the initiation context surrounding the start codon. The second mechanism is the internal translation initiation mechanism that uses an internal ribosome entry site (IRES) secondary structure to favor the cap-independent recruitment of the translation initiation complex, and drives the translation initiation at the third start codon (15) (Figure 1A). Because all start codons are in-frame, the resulting products of protein synthesis are thus protein isoforms that differ only in the length of their amino (N)-terminal extensions (Figure 1B). All isoforms possess both the ubiquitin binding ligand (UBL) and the BAG domains at the C-terminal end, domains that are essential for protein-protein interaction with most of the known partners. The isoforms differ in the number of acidic repeats found in the N-terminal region. The BAG-1L isoform is the only one that possesses a nuclear localisation signal (NLS) at its N-terminal end that triggers its localization in the nucleus. BAG-1M shuttles between the nucleus and the cytoplasm, and BAG-1S, the most abundant isoform, is cytoplasmic.

The different isoforms of BAG-1 are known to be overexpressed in many different cancers (16), including colorectal cancer (CRC) (11,17,18). The highest expression of the protein is observed in the late stages of colorectal tumorigenesis (11), and the overexpression of the long isoform BAG-1L is associated with a poorer prognosis (19,20). Notably, BAG-1 silencing in CRC cells induces apoptosis (21,22). Thus, BAG-1 is considered as being a possible therapeutic target in CRC (23), as well as in other cancer types (24–26).

Notably, the 5′UTR of the BAG-1 transcript contains several regulatory features at the levels of RNA sequence and RNA secondary structure (Figure 1C). First, the four alternative in-frame start codons are all located in suboptimal Kozak contexts for translational initiation. Second, the translation of the longest isoform, BAG-1L, is initiated at a non-canonical CUG start codon. Third, the translation of the most abundant isoform, BAG-1S, is regulated by an internal ribosome entry site (IRES) (15). The nucleotides located at positions 247–432 of the 5′UTR adopt a defined secondary structure element that recruits the IRES *trans*-acting factors (ITAF) PTB-1 and PCBP1. This allows the opening of a ribosome binding site (RBS) window and the recruitment of the 40S ribosomal subunit inside the 5′UTR, thereby initiating translation in a manner independent of 5′ cap-recognition (27). This specific regulation of the BAG-1S isoform happens under stress-related conditions such as heat-shock and chemotoxic stresses (15,28) where the canonical cap-dependent translation is repressed. Finally, a G-quadruplex secondary structure located at the 5′ end of the 5′UTR of the BAG-1 mRNA, specifically at positions 6 to 35 (29) (Figure 1A and C), was previously probed *in vitro* and shown to repress the expression of a luciferase reporter gene in three CRC cell lines (30). Based on motif search and bioinformatic rG4 predictions (30–32), the BAG-1 rG4 located at this position in the 5′UTR is the only probable rG4 of the full BAG-1 transcript.

G-quadruplexes (G4) are very stable non-canonical secondary structures formed by G-rich DNA or RNA nucleotide sequences. In a sequence presenting a minimum of four tracts of two or more continuous guanines, each guanine of the tract can form base-pairs with those from the next tracts through Hoogsteen hydrogen bonds, resulting in a co-planar array called the G-quartet. The stacking of two or more G-quartets forms a G4. In an intramolecular G4, the G-quartets are linked to each other via three stretches of random nucleotides that form the loops. The G4s are stabilized by the presence of a monovalent cation, mainly potassium, the most abundant cation in the cell.

RNA G4 (rG4) are highly abundant (33) and are folded *in cellulo* (34). They are involved in many post-transcriptional regulatory mechanisms such as alternative splicing, polyadenylation and mRNA localization (35). Emerging evidence also suggest that G4s dysfunction may be involved in the pathogenesis of diseases such as cancer and neurological disorders (36).

RG4s are specifically bound by RNA-binding proteins and helicases that regulate their formation (37). The rG4s located in the 5′UTR are principally described as being translational repressors (38). The proposed mechanism is a steric blocking of both the translation initiation and the ribosomal scanning because of their high stability (39). However, in contrast to the majority of rG4s, the rG4s located in the 5′UTRs of the VEGF and the FGF-2 transcripts were identified as activators of translation, by being parts of IRES secondary structures that are essential for the cap-independent translation of these mRNAs (40,41). Despite their high 5′UTR abundance, and their known role in translational regulation, the interactions of rG4s with other *cis* translational regulation motifs located in 5′UTRs, such as alternative start codons, non-canonical start codons and IRES, remain unclear.

The translation of the BAG-1 mRNA transcript is regulated by both non-canonical cap-dependent and cap-independent translation mechanisms. The impacts of a 5′UTR rG4 on the translational regulation of a transcript when both types of regulation are simultaneously present has never been analyzed. In this study, we demonstrate that the rG4 region of the BAG-1 mRNA, located upstream of all known translational regulatory elements of the 5′UTR, acts in the control of the BAG-1 mRNA different types of translation and expression, in the context of CRC, through maintenance of the 5′UTR global secondary structure.

## MATERIALS AND METHODS

### Paired colorectal tumor tissue samples

Total protein lysates in RIPA buffer (25 mM Tris–HCl pH 7.6, 150 mM NaCl, 1% NP-40, 1% sodium deoxycholate, 0.1% SDS) and complementary DNAs (cDNA) resulting from the reverse-transcription (RT) of the total RNA extracted from 50 specimens of paired tumoral and healthy colorectal tissues were obtained from a previously described biobank (42). The healthy tissue consists of the margin located at least 10 cm away from the tumor. The tissues were obtained from patients, who did not receive neoadjuvant therapy, undergoing surgical resection. The tissues were processed, classified and graded as previously described (42). The clinicopathological parameters of the patients and tumors are described in Supplementary Table S1. The protocol was approved by the Institutional Human Subject Review Board of the Centre Hospitalier Universitaire de Sherbrooke and the patients’ written, informed consents were obtained.

The BAG-1 mRNA levels were determined by qPCR in human advanced adenomas and adenocarcinomas, and were compared to the paired adjacent healthy tissue for 46 samples (Adenoma *n* = 8; Stage 1 *n* = 8; Stage 2 *n* = 10; Stage 3 *n* = 10; and, Stage 4 *n* = 10). The BAG-1 protein isoform levels were determined by Western blot analysis for 38 pairs of samples (Adenoma *n* = 7; Stage 1 *n* = 7; Stage 2 *n* = 8; Stage 3 *n* = 8; and, Stage 4 *n* = 8). Only a small number of tissue pairs were not in common between both analyses (Adenoma *n* = 3; Stage 1 *n* = 1; Stage 2 *n* = 2; Stage 3 *n* = 2 and Stage 4 *n* = 2).

### Cell culture

The HCT116 colorectal cancer cell line (ATCC, CCL-247) was cultivated in McCoy's 5A medium supplemented with 10% foetal bovine serum (FBS) in a 37°C incubator with a 5% CO_2_ atmosphere. All cell culture reagents were obtained from Multicell, Wisent.

### Treatment of cells with G4-specific chemical ligands

HCT116 cells were seeded at 650 000 cells/well in six-well plates, 24 h prior to treatment. Along with 1 μl/well of lipofectamine 2000 (ThermoFisher), the ligands were then added to the media at final concentrations of 2 μM cPDS (carboxypyridostatin trifluoroacetate salt, Sigma-Aldrich, working solution 1 mM in water), 20 μM Phen-DC3 (Polysciences Inc., working solution 2 mM in DMSO) and 2 μM TmPyP4 (meso-5,10,15,20-Tetrakis-(*N*-methyl-4-pyridyl)porphine, Calbiochem, working solution 1 mM in water) and the cells incubated for 24 h at which point they were compared to vehicle-only treated cells. All treatments were performed in either triplicate (for cPDS) or duplicate (Phen-DC3 and TmPyP4), and were repeated on two different days (*n* = 2). Cells from each well were harvested in 1 ml of ice-cold PBS using a cell scraper. The cell volumes equivalent to 1/5 and 4/5 of a well of a six-well plate were kept for the total RNA and the total protein extractions, respectively. Centrifugation at 1000 RPM for 10 min was performed to isolate the cell pellets, which were then stored at –80°C until the lysis and the RNA and protein extractions were performed.

### Design and cloning of the gene reporter constructs

#### PsiCHECK-2 luciferase reporter

The complete WT, rG4mut and the 1S start codon mutated sequences of the BAG-1 5′UTR with flanking NheI restriction sites were ordered from and chemically synthesized by Biomatik. After NheI digestion, the 5′UTR was ligated to the psiCHECK-2 dual-luciferase reporter plasmid (Promega) upstream of the Rluc coding sequence (CDS). In order to ensure that all start codons were in-frame with the Rluc CDS, two extra nucleotides were added by primer directed mutagenesis. The 1L, 1M and AUG-254 start codons were mutated using primer directed mutagenesis. All sequences were verified by DNA sequencing.

#### pRL-L bicistronic luciferase reporter

The pRL-HL bicistronic luciferase reporter plasmid consists of the Rluc reporter gene, expressed via cap-dependent translation, followed by the NotI restriction site, all located upstream of the HCV IRES sequence that controls the Fluc expression in a cap-independent fashion. The bicistronic plasmid was modified by directed mutagenesis so as to insert a HpaI restriction site at the 3′ end of the HCV IRES. The removal of the HCV IRES sequence was performed by digesting the vector with the NotI and HpaI restriction enzymes. Both the BAG-1 complete 5′UTR WT and the rG4 mutant were amplified from the psiCHECK-2 constructions using primers that inserted both the NotI and HpaI restriction enzyme sites, and the amplicons were then digested and ligated in between the two luciferase reporter genes so as to create either the pRL-BAG1wt or the G4mut-L bicistronic vectors. Other mutations in the BAG-1 IRES sequence, stem3mutA and stem3mutB, were generated using primer directed mutagenesis and were verified by DNA sequencing.

The complete list of sequences used in this study, as well as the list of primers, are available in Supplementary Tables S4 and S5.

### Transfections and luciferase assays

#### Transfection of the monocistronic psiCHECK-2 luciferase reporter construct

Twenty-four hours prior to transfection, HCT116 cells were seeded at 650 000 cells/well in a six-well plate. The cells were transfected using 125 ng/well of the psiCHECK-2 construction along with 2375 ng/well of the carrier plasmid PUC19 using 2.5 μl/well of lipofectamine 2000 (ThermoFisher) in serum-free media. The serum was added 4 h after transfection. Twenty-four hours later, the cells were harvested on ice using 1 ml of PBS 1× and a cell scraper. The cell lysate was divided in three parts: 1/5 (200 μl) for qPCR, 1/5 (200 μl) for the luciferase assay and 3/5 (600 μl) for western blot.

#### Transfection of the bicistronic pRL-L luciferase reporter construct

Twenty-four hours prior to transfection, HCT116 cells were seeded at either 300 000 cell/well in 12-well plates, or at 100 000 cells/well in a 24-well plates. The cells in the 12-well plates were transfected using 1000 ng/well of the bicistronic constructions and 2 μl/well of lipofectamine 2000 while those in the 24-well plated received 500 ng/well and 1 μl/well, all in serum-free media. The serum was added 4 h after transfection. The cells were harvested 24 h later. For the experiments performed in the 12-well plates, half of the cells (500 μl) were used for qPCR and half for the luciferase assay. In the case of 24-well plates, all of the cells were recovered and lysed in order to perform the luciferase assay.

#### Luciferase assay

The DualGlo luciferase assay kit from Promega was used according to the manufacturer's protocol. Briefly, the cells were lysed in the corresponding cell volume amount of the kit's 1× passive lysis buffer. A volume of 5–10 μl of the cell lysate was used, and 100 μl of each of the Fluc and Rluc luciferase substrates was added sequentially. Readings of 5 s were performed using a Glomax 20/20 luminometer.

### Western blot

#### Endogenous BAG-1 isoforms in colorectal tumors and paired margins

Proteins (20 μg) derived from the total protein lysates of the tissue samples obtained from the biobank were separated on a 10% SDS-PAGE gel, and then was transferred to a polyvinylidene difluoride (PVDF) membrane. The membrane was blocked for 30 min at room temperature in Tris buffered saline (TBS) with 2.5 % (w/v) nonfat dry milk, then it was incubated overnight (O/N) at 4°C with the primary mouse mAb anti-BAG-1 antibody (CC9E8, Santa Cruz Biotechnologies) which had been diluted 1:100 in phosphate buffered saline (PBS) with 2.5 % (w/v) nonfat dry milk (PBS-milk 2.5 %). After three washes in PBS with 0.1 % Tween-20 (PBS-T), the membrane was incubated for 1 h at room temperature with the secondary anti-mouse IgG (H+L)-Alkaline phosphatase-conjugated antibody (Promega) at a dilution of 1:7500 in PBS-milk 2%. After two washes for 10 min each with PBS-T, and one with PBS only, the membrane was developed using 1 ml of alkaline phosphatase substrate (CDP-Star (Applied Biosystems) diluted to 1× in 100 mM Tris pH 9.5 and 100 mM NaCl buffer). The membrane was rinsed with PBS-T, and then was exposed to an X-ray film for diverse exposure times. The loading control ERK2 was obtained via 2 h of incubation at 37°C of the membrane with the rabbit anti-ERK2 antibody (C14, Santa Cruz Biotechnologies) diluted 1:5000 in PBS-milk 5%. After washes with PBS-T, the membrane was incubated for 1 h at room temperature with the secondary anti-rabbit L-HRP antibody (NA934, GE Healhcare) diluted 1:5000 in PBS-milk 5%. Revelation was performed using the western lightning plus-ECL enhanced chemiluminescence substrate (PerkinElmer), and was detected using the ImageQuant LAS4000 machine (GE Healthcare). Quantification of the band densities was obtained using the ImageJ software.

#### Endogenous BAG-1 in HCT116 cells treated with ligands

The cell lysis for total protein extraction was performed by the addition of 100 μl of 1.5× Laemmli buffer (3.75% SDS, 15% Glycerol, 150 mM Tris–HCl pH 6.8) per cell pellet that corresponded to 4/5 of a well of a six-well plate of treated cells. The samples were boiled for 5 min at 90°C, and were then sonicated twice for 2 s at 16% amplitude. The samples were then centrifuged for 1 min at 13 000 RPM, and the protein concentration in the supernatant was evaluated using the BCA assay (Pierce) according to the manufacturer's protocol. Protein samples (30 μg) were loaded on a 10% SDS-PAGE gel, and the western blot against the endogenous BAG-1 protein was performed as described above. After membrane stripping with two washes for 10 min each with NaOH 0.5 N and one wash for 10 min in PBS, the membrane was blocked for 20 min in PBS-milk 2.5%. The membrane was then incubated O/N at 4°C with the loading control anti-β-actin mouse mAb antibody (A5441, Sigma) diluted 1:1 000 in PBS-milk 2.5%. After three washes for 10 min each with PBS-T, the membrane was incubated for 1h at room temperature with the secondary anti-rabbit L-HRP antibody (NA934, GE Healhcare) diluted 1:5000 in PBS-milk 5%. Revelation was performed using the western lightning plus-ECL enhanced chemiluminescence substrate (PerkinElmer), and was detected using the ImageQuant LAS4000 machine (GE Healthcare). Quantification of the band densities was performed using the ImageJ software. The BAG-1 protein isoforms abundance levels were normalized over the abundance level of the β-actin loading control.

#### N-terminal extended Rluc isoforms in transfected HCT116 cells

Using a cell volume equivalent to 3/5 of a six-well plate of transfected cells, the lysis was performed with 70 μl of 1.5× Laemmli buffer. Protein samples (30 μg) were migrated on a 10%, SDS-PAGE gel. The gel was then transferred to a nitrocellulose membrane. The membrane was blocked in PBS-Milk 4% for 15 min at room temperature, and then was incubated O/N in PBS-Milk 4 % with the primary antibodies rabbit polyclonal Anti-Renilla Luciferase antibody (PM047, MBL) diluted 1:1000 and anti-β-actin mouse mAb (A5441, Sigma) diluted 1:2000. The membrane was washed three times for 10 min with PBS–Tween 0.1 %, and then was incubated for 1 h at room temperature with the secondary antibodies Alexa fluor-680 Goat anti-Mouse IgG (A21057, Life technologies) and IRDye 800CW Donkey anti-Rabbit IgG (#926–32213, Mandel), both diluted 1:10 000 in PBS-Milk 4%. After three washes for 10 min each with PBS-T, the membrane was revealed using the Odyssey imaging system (LI-COR Biosciences). Quantification of the band densities was obtained using the ImageJ software.

### Total RNA extraction from HCT116 cells and DNase treatment

The total RNA extraction was performed using a cell volume corresponding to 1/5 of a six-well plate, or 1/2 of a 12-well plate, depending on the experiment described in the previous sections. The cells were homogenized with 250 μl of QIAazol (QIAGEN). The RNA extraction was then performed by adding 50 μl of chloroform, incubating at room temperature for 2 min and centrifuging at 13 000 RPM for 15 min. The aqueous phase was then transferred into a new tube and the RNA was precipitated by the addition of 125 μl of isopropanol. After a 5 min incubation at room temperature and a centrifugation at 13 000 RPM at 4°C for 20 min, the resulting RNA pellet was washed with 225 μl of 70 % ethanol and centrifuged again at 13 000 RPM at 4°C for 10 min. The resulting pellet was air dried and dissolved in 30 μl of H_2_O.

The RNA samples were treated with DNase prior to RT-PCR. Briefly, 1 μg of total RNA was incubated in a final volume of 10 μl with 1 μl of 10× DNase reaction buffer and 1 unit of RQ1 RNAse-free DNAse (both from Promega) for 30 min at 37°C. After incubation, 90 μl of H_2_O were added and the RNA was recovered by phenol-chloroform extraction followed by ethanol precipitation. RNA pellet was dissolved in 5 μl H_2_O (resulting in a concentration of ∼200 ng/μl) prior to be sent to the RNomics Platform of the Université de Sherbrooke for RNA quality control evaluation and the reverse transcription and qPCR reactions.

### RNA quality control, reverse transcription and qPCR

All of these steps were performed by the RNomics Platform of the Université de Sherbrooke. RNA integrity was assessed using an Agilent 2100 Bioanalyzer (Agilent Technologies). Reverse transcription (RT) was performed on 1.1 μg total RNA with final concentration of 10 units of Transcriptor reverse transcriptase, 60 μM of random hexamer, 1 mM each dNTP (all from Roche Diagnostics) and 10 units of RNAseOUT (Invitrogen*)* according to Roche Diagnostics’ protocol in a total volume of 10 μl. All forward and reverse primers were individually dissolved to 20–100 μM stock solution, in 10 mM Tris, 0.1 mM EDTA buffer (Integrated DNA technologies, IDT), and were then diluted as a primer pair to 1 μM in RNase DNase-free water (IDT). Quantitative PCR (qPCR) reactions were performed in a 10 μl final volume in 384-well plates on a CFX-384 thermocycler (BioRad) with 5 μl of 2× iTaq Universal SYBR Green Supermix (BioRad), 10 ng (3 μl) of cDNA and a 200 nM final primer pair concentration (2 μl). The following cycling conditions were used: 3 min at 95°C; and, 50 cycles of: 15 s at 95°C, 30 s at 60°C and 30 s at 72°C. The relative expression levels were calculated using the qBASE framework (43) and the housekeeping genes YWHAZ, MRPL19, PUM1 and SDHA for human cDNA. Primer design and validation was evaluated as described elsewhere (44). In every qPCR run, a no-template control was performed for each primer pair and these were consistently negative. All primer sequences are available in Supplementary Table S4.

### mRNA mono- and bi-cistronic luciferase reporter assays

#### Preparation of the mRNA transcripts

First, the pRL-intercistronBAG1wt and G4mut-L plasmids were created starting from the pRL-BAG-1-L reporter plasmid using primer directed mutagenesis. A 74 nts long intercistron region was added between the RLuc CDS and the complete 5′UTR sequence of BAG-1. Its function was to extend the 3′ extremity after the Rluc CDS in order to augment the stability of the resulting Rluc monocistronic mRNA construct. The DNA templates used for *in vitro* transcription to create both the mono- and bicistronic mRNA constructs (capped and poly-adenylated) for transfection were created by the amplification of either the pRL-intercistronBAG1wt or the G4mut-L plasmid using different sets of primers. The primers were designed so as to add the T7 promoter in 5′ and a 60 nts long poly-A tail in 3′. The primers used are listed in Supplementary Table S4, and the complete mRNA sequences of each construction are listed in Supplementary Table S6. After PCR amplification, the DNA templates were digested with the DpnI restriction enzyme so as to remove any remaining plasmid nucleotides, and were then purified using the PCR purification kit (Biobasic Canada inc.) according to the manufacturer's protocol.

*In vitro* transcription and capping of the mRNA with either the m^7^G-cap or the analog A-cap was performed using the mMessage mMachine Kit (Ambion) according to the manufacturer's instructions. The only alteration to the protocol was to generate the mRNA constructions capped with the A-cap analog. The 2XNTP/CAP solution from the kit was replaced by a G(5′)ppp(5′)A RNA cap structure analog (NEB) in order to obtain the 2X NTP/Analog solution with final concentrations of 12 mM A-cap analog, 15 mM each of rATP, rCTP and rUTP and 3 mM rGTP. After transcription, DNase treatment and lithium chloride precipitation were performed following the manufacturer's protocol, and samples of the mRNA constructions were verified on denaturing agarose gels for their integrity.

#### mRNA transfection and luciferase assay

HCT116 cells were seeded at 160 000 cells/well in 24-wells plates 24h prior to transfection. A total amount of 500 ng of mRNA constructions (either 250ng of the Fluc monocistronic constructions co-transfected with 250 ng of the RLuc monocistronic control, or 500 ng of a bicistronic construction) were transfected using 1 μl/well of lipofectamine 2000 in serum-free media. The cells were harvested 4 h after transfection. Half of the cell volume was used for the DualGlo luciferase assay (Promega) following manufacturer's protocol (as described previously). The remaining half of the cell volume was kept for total RNA extraction in order to perform the RNA level quantifications by reverse transcription (as described previously), followed by the droplet digital PCR (ddPCR) quantification of the cDNA. The ratio of Fluc/Rluc luciferase expression levels of each construction was corrected by dividing it by the corresponding Fluc/Rluc RNA levels ratio as measured by the ddPCR quantification. The results are reported as a percentage relative to the m^7^G-cap WT monocistronic construction set to 100%.

### ddPCR quantification

The ddPCR quantification was performed by the RNomics Platform of the Université de Sherbrooke. Briefly, the ddPCR reactions for both Fluc and Rluc were composed of 10 μl of 2X QX200 ddPCR Supermix for probe (Bio-Rad), 10 ng (3 μl) of cDNA, a 250 nM final concentration of the probe solutions for both Fluc (FAM, from IDT) and Rluc (HEX, from IDT) and a 0.9 μM final concentration of the primer pair solutions for each target gene in a 20 μl reaction. The ddPCR fourplex reactions for the Reference genes were composed of 10 μl of 2× QX200 ddPCR Supermix for probe (Bio-Rad), 10 ng (3 μl) cDNA, a 250 nM final concentration of the probe solutions for MRPL19 (FAM) and YWHAZ (HEX), a 125 nM final concentration for the probe solutions for both PUM1 (FAM) and B2M (HEX) and a 0.9 μM final concentration of the primer pair solutions for each reference gene in a 20 μl reaction.

Each reaction mix (20 μl) was converted to droplets with the QX200 droplet generator (Bio-Rad). Droplet-partitioned samples were then transferred to a 96-well plate, sealed and cycled in a C1000 deep well Thermocycler (Bio-Rad) using the following cycling protocol: 95°C for 5 min (DNA polymerase activation); 50 cycles of 95°C for 30 s (denaturation), 59°C for 1 min (annealing) and 72°C for 30 s (extension); and, post-cycling steps of 4°C for 5 min, 90°C for 5 min (signal stabilization) and a hold at 4°C. Reference gene reactions were cycled using the following cycling protocol: 95°C for 5 min (DNA polymerase activation); 50 cycles of 94°C for 30 sec (denaturation), 59°C for 1 min (annealing/extension); and, post-cycling steps of 98°C for 10 min (enzyme deactivation) and a hold at 4°C. The cycled plate was then transferred and read using the QX200 reader (Bio-Rad) either immediately or the next day. The concentrations reported are in copies/uL of the final 1x ddPCR reaction (using QuantaSoft software from Bio-Rad) (45).

### SHAPE and RNA secondary structure analyses

#### Transcription of RNA

The DNA templates for the *in vitro* transcription of the RNAs to be used for SHAPE were created by the amplification of 5 ng of the psiCHECK-2 constructions with either the complete WT or the rG4mut BAG-1 5′UTR using primers that inserted the RNA polymerase T3 promoter binding site at the 5′ end of the BAG-1 5′UTR and which conserved the next 40 nts of the psiCHECK-2 plasmid sequence at the 3′end of the BAG-1 5′UTR. The BAG-1 5′UTR with the IRES mutated sequences were amplified from either the WT or the rG4mut pRL-BAG1-L constructions. In so doing, the T3 promoter binding site was inserted upstream of the BAG-1 5′UTR, and the same 40 nts-long 3′ end flanking sequence that was inserted into the constructs originating from the psiCHECK-2 plasmid was inserted in 3′. The primers used for DNA template preparation and amplification are listed in Supplementary Table S4, and the full RNA sequences used for SHAPE are listed in Supplementary Table S5. The *in vitro* transcription was performed as described previously (46).

#### Selective 2′-hydroxyl acylation analyzed by primer extension

The pre-folding of RNA (5 pmol) was performed in folding buffer (20 mM Li Cacodylate pH 7.5, 100 mM KCl) in a total volume of 10 μl. The RNA was incubated for 5 min at 75°C, and then was slow-cooled to room temperature for 1 h. The selective 2′-hydroxyl acylation reaction was performed by adding either 1 μl of a freshly prepared 600 mM solution of the SHAPE reagent Benzoyl Cyanide (BzCN, CAS#613-90-1, Sigma-Aldrich, dissolved in DMSO) or 1 μl of DMSO (no SHAPE reagent control) and incubating for 1 min at room temperature. A volume of 90 μl of H_2_O was then added, the RNA was ethanol precipitated and the resulting pellet dissolved in 10 μl of 0.5X TE buffer (5 mM Tris–HCl pH 7.5, 0.5 mM EDTA). Subsequently, the primer extension step was performed using the Superscript III Reverse transcriptase (Life technologies). Two primer extension reactions were performed in parallel using two different 6-FAM-labeled primers (Applied Biosystems), one for each reaction. Primer 1 bound the flanking 28 nts located at the 3′ end, and primer 2 the middle of the 5′-UTR (at positions 301–320) in order to compensate for the reduced reverse transcription of the enzyme after ∼300 nts. The RNA was unfolded by heating at 95°C for 3 min, and was then snap-cooled on ice. Annealing of the 6-FAM labeled primers (1 pmol) was performed by heating at 65°C for 5 min; then at 37°C for 5 min and finally at 4°C for 1 min. The reverse transcriptase reaction was then performed for 30 min at 52°C in a buffer with final concentrations of 1× first strand buffer, 10 mM DTT, 1 mM of each dTNP and 20% DMSO.

In order to obtain the DNA sequencing reactions necessary for the subsequent quantitative SHAPE analysis, primer extensions reactions were performed on untreated RNA sequences. The primer extension reactions required in order to obtain the sequencing reactions were performed under the same conditions as the reverse transcriptase reactions using 5 pmol of RNA without pre-folding in the presence of an additional 1 mM of either ddCTP or ddGTP and using the corresponding NED-labeled primer 1 or 2 (Applied Biosystems). The fluorescent primers used are listed in Supplementary Table S4. Following the primer extension reactions for both the SHAPE reactions and the sequencing reactions, 2 μl of 2 N NaOH was added to each and the samples heated at 95°C for 5 min in order to degrade the RNA. The cDNA samples were ethanol precipitated, and the resulting pellet air-dried. Capillary electrophoresis of the cDNA was performed at a sequencing and genotyping facility (Plateforme de séquençage et de génotypage; CHUL, Québec, Canada). There, the DNA pellets were dissolved in a mixture of 10 μl each of H_2_O and formamide with the addition of a Lyz labelled control DNA ladder (Life Technologies). Each SHAPE reaction and no SHAPE reagent control reaction was electrophoresed in the presence of the ddCTP sequencing reaction on an ABI 3100 Genetic Analyzer (Life Technologies). The electrophoresis was then repeated with both the SHAPE and the no SHAPE control reactions in the presence of the ddGTP sequencing reactions.

#### Quantitative SHAPE analysis and secondary structure prediction

The quantitative SHAPE reactivity for each nucleotide was determined from the electropherograms using the QuSHAPE software version 1.0 (47). The normalized reactivity for each nucleotide was then averaged from the four SHAPE experiments (two replicates with primer 1 and two replicates with primer 2) and used as pseudo-energy constraints for RNA secondary structure prediction using the default slope (1.8 kcal/mol) and intercept (–0.6 kcal/mol) values in the Fold tool of the RNAstructure software version 5.7 (48). Comparison and clustering of the ensemble of possible secondary structures respecting the SHAPE constraints for the different RNA sequences was performed using the StructureXplore software (49). The predicted minimum free energies (MFE), in kcal/mol, of the different regions of the secondary structures were evaluated using the *RNAeval* function of the ViennaRNA package (50). The secondary structure representations were made with VARNA (51), and the Arc-plots were made with R-CHIE (52).

### Statistical analysis

All statistical analysis and tests were performed using GraphPad Prism version 7.03 for Windows (GraphPad Software, La Jolla, CA, USA, www.graphpad.com). Each statistical test performed, including the number of replicates and the number of independent experiments, are indicated in the figure legends. *P*-values < 0.05 were considered as being significant.

## RESULTS AND DISCUSSION

### BAG-1 expression is post-transcriptionally regulated in CRC cell lines and tumors

The rG4 located in the 5′UTR of the BAG-1 mRNA was identified during an analysis of the rG4s associated with the CRC pathway that could affect mRNA translation (30).The initial step was thus to confirm the post-transcriptional regulation of the BAG-1 mRNA in human CRC cells. In order to do so, the expression levels of the BAG-1 mRNA and of its protein isoforms were measured in paired tissue samples extracted from colorectal tumors at different stages and from their surrounding healthy margins. The stages of the tumor (adenoma to stages 1 to 4) represent the size and the degree of invasiveness of the tumor, as compared to the nearby tissue and lymph nodes, and the presence or not of metastasis. A higher stage represents a more advanced tumor progression (53). These analyses were also performed in both normal and CRC cells in culture. It was initially speculated that if BAG-1 expression is indeed post-transcriptionally regulated in colorectal tumor settings, the RNA levels should not correlate with the protein isoform levels.

**Figure 2.**
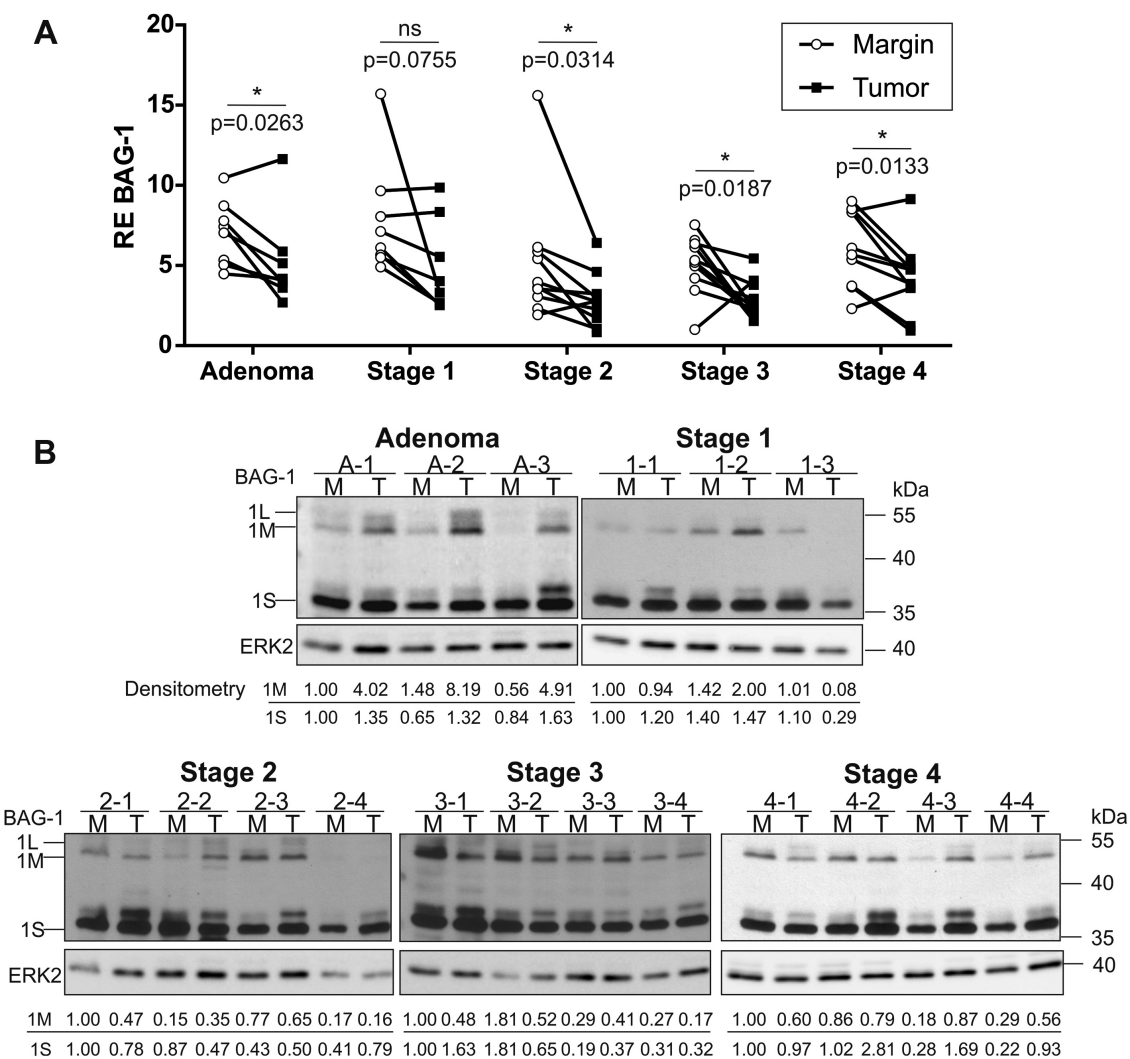
RNA and protein expression levels of BAG-1 in the paired tissues of colorectal tumors at different stages and in their adjacent healthy tissue (margin). (**A**) Relative expression levels of the BAG-1 mRNA in the paired adjacent healthy tissues (Margin, white circle) and the tumors (black square) at different stages as measured by RT-qPCR. Each tissue pair is connected by a line (*n* = 8 for adenoma and stage 1, *n* = 10 for stages 2, 3 and 4). The statistical analysis performed was a paired t-test, with the tumor being compared to the margin. ns = no significant difference, * *P*≤ 0.05. (**B**) Protein expression levels, as measured by Western blot, of the three BAG-1 isoforms in the same pairs of margin-tumor tissues as in (A). The positions of the protein ladder are indicated on the left. ERK2 was used as the loading control. The relative densities of the 2 most abundant isoforms, BAG-1M and BAG-1S, are annotated under each lane. The isoforms’ band densities were corrected on the corresponding ERK2 loading control band density and reported relative to the density, of the isoform of the first lane of each blot which was set to 1.00 (*n* = 3 for adenoma and stage 1, *n* = 4 for stages 2, 3 and 4). The Western blots of the remaining tissue pairs are available in the supplementary material.

The RNA levels were compared in eight tissue pairs for each of the adenomas and the stage 1 tumors, and in ten tissue pairs for each tumor of stages 2, 3 and 4. With the exception of the stage 1 tumors, all of the tumor stages demonstrated significant decrease in the BAG-1 mRNA levels expressed in tumors compared to their normal adjacent tissues (margins) (Figure 2A). The tumor over margin (T/M) fold changes of the RNA expression levels for each pair are listed in Supplementary Table S2.

Next, we measured the protein levels of the three BAG-1 protein isoforms by western blotting using an antibody that recognizes their common C-terminal regions. The protein extracts were derived from the same paired tissue samples as in the Figure 2A. An increase in all isoforms was observed in adenoma tissues compared to their margins (Figure 2B and Supplementary Figure S1). An increase in protein isoform expression levels, or differences in the molecular weights of the isoforms, were also observed in certain tumors from stages 1 to 4 (Figure 2B and Supplementary Figure S1). These differences between tumors could arise from intrinsic heterogeneity, and/or differentiation state of the tumors between patients. In this regard, BAG-1 isoforms levels differ along the crypt axis and during development of the colon (18,54). Changes in molecular weight can be attributed to post-translational modifications (55). The loss of expression of the longer isoforms BAG-1L and BAG-1M was also observed in one stage 2 tumor. Thus, in contrast to the mRNA levels that decreased in the tumors regardless of the stage, the protein levels were either maintained or increased in the tumors. Supplementary Table S2 presents the quantified T/M-fold changes of the RNA and the protein isoforms levels for each tissue pair. The T/M-fold changes of the protein levels were divided by the T/M-fold changes of the transcript levels. If the RNA levels were correlated with the protein levels of the isoforms, the obtained ratio would be equal to or close to 1. A ratio higher or lower than 1 represents an absence of correlation. Interestingly, an absence of correlation is observed for five out of six adenomas tissue pairs: four out of seven stage one pairs; six out of eight stage two pairs; three out of eight stage three pairs; and, seven out of eight stage four pairs. The tumors presented a higher protein abundance than what it would be expected from the RNA level only. The increase in protein expression, associated with the decrease in the RNA level, is observed more frequently in adenomas and stage 4 tumors. This absence of correlation between the mRNA and protein expression levels suggests a post-transcriptional regulation for the BAG-1 mRNA in colorectal tumors.

This hypothesis was also supported by the RNA and protein levels measured in nine CRC cell lines, and that were compared to two normal intestinal epithelial cell lines (HIEC and CRL-1831). The BAG-1 mRNA level was decreased by at least 2-fold in CRC cell lines as compared to normal cells (Supplementary Figure S2A). The levels of the three BAG-1 isoforms in pooled protein lysates from seven of the CRC cell lines were measured by Western blot and compared to the BAG-1 isoform protein levels in normal HIEC. Different expression levels of the BAG-1 protein isoforms were observed depending on the cell line used, but all CRC cell lines exhibited an increase in the expression of the BAG-1 isoforms as compared to normal HIEC (Supplementary Figure S2B). This observation is reminiscent to previous studies (11,17,18) which also reported an increased BAG-1 protein expression levels in various CRC cell lines. Accordingly, proteogenomic analyses previously demonstrated that the mRNA abundance is not a good predictor of the protein abundance in both colon and rectal tumors (56). Thus, our results from both colorectal cell lines, and tumors indicate that BAG-1 expression is post-transcriptionally regulated since its RNA levels did not correlate with its protein levels.

### Stabilization of the rG4 using chemical ligands decreases endogenous BAG-1 protein isoform's expression

**Figure 3.**
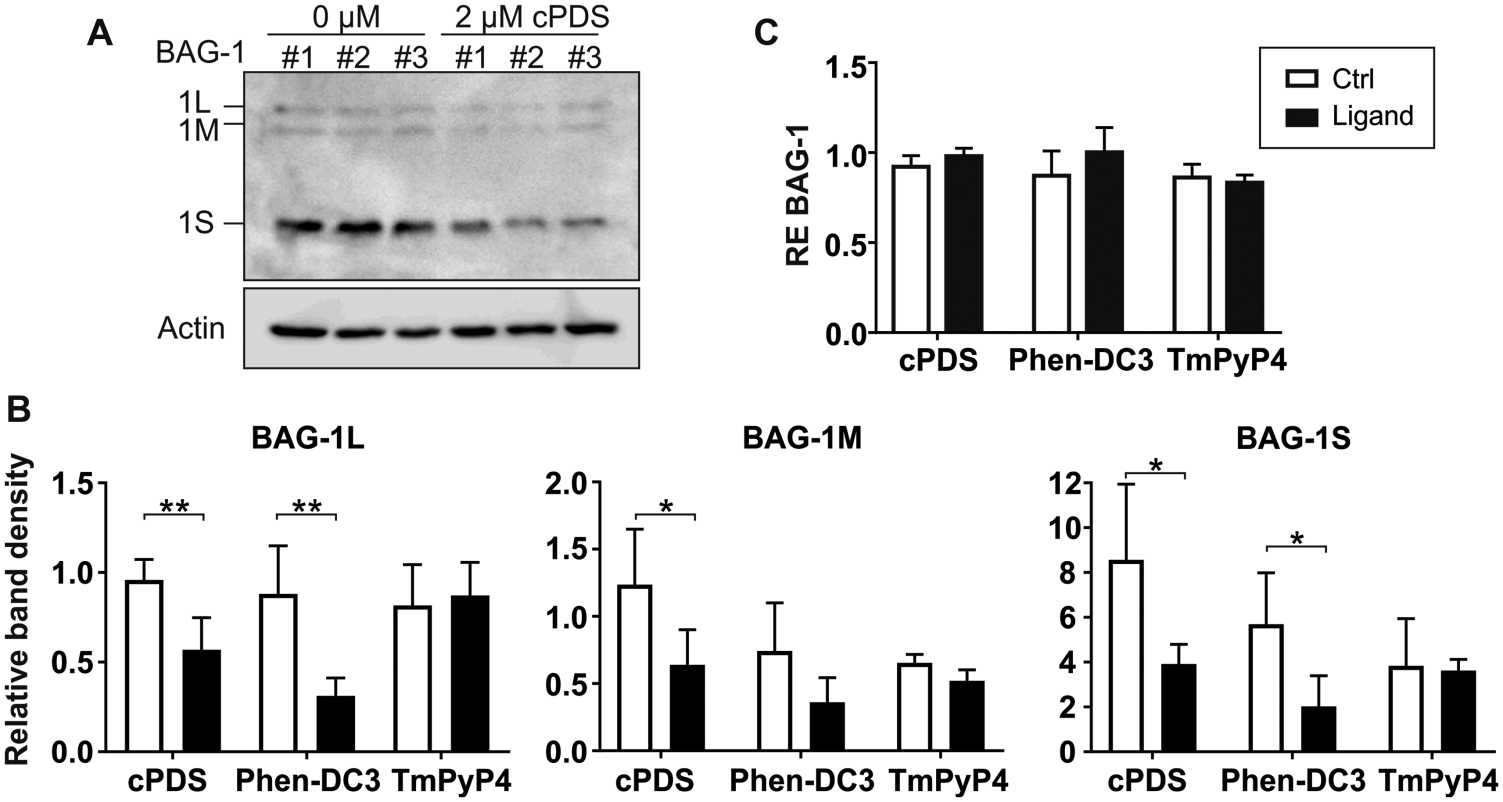
Stabilization of the rG4 with chemical ligands. (**A**) A representative gel of the BAG-1 endogenous protein isoforms from HCT116 cells after a 24 h treatment with 2 μM of the specific rG4 ligand cPDS compared to that of an untreated control. (**B**) Relative densitometry of the BAG-1 isoforms’ protein bands after a 24 h treatment of the cells with the ligand (black bar), or of an untreated control (white bar). The ligand concentrations used were: cPDS 2 μM, Phen-DC3 10μM and TmPyP4 2μM. (**C**) Relative expression levels of the BAG-1 mRNA in the ligand treated cells as compared to the untreated cells. For both (**B**) and (**C**), the results are presented as the means and standard deviations of *n* = 2 (each ligand treatment was performed in triplicate). The statistical analyses performed were an unpaired t-test between the ligand treated and the control untreated cells for each ligand. *P≤ 0.05, **P≤ 0.001.

BAG-1 expression is post-transcriptionally regulated in both CRC cell lines and tumor samples. As rG4s are generally described as being translational repressors, the recently identified rG4 localized in the 5′UTR of the BAG-1 mRNA could regulate the expression of the BAG-1 isoforms. To verify this hypothesis, CRC cells (HCT116) were treated with the RNA quadruplex specific ligand cPDS (57). The binding of this ligand stabilize any rG4 structure and not the BAG-1 rG4 specifically. As most rG4s located in the 5′UTR impede translation due to steric hindrance caused by their high stability, further stabilization of the structure upon ligand binding enhances this repressive effect (58, 59). We expected that if the 5′UTR of the BAG-1 transcript really adopted the rG4 folding *in cellulo*, the cPDS ligand would bind it, stabilize it and further impede the transcript translation. Thus, lower level of the isoforms would be observed in the cPDS condition compared to an untreated sample. Upon treatment with 2 μM cPDS, the protein levels of the three endogenous BAG-1 isoforms decreased by almost 2-fold (Figure 3A and B). Nevertheless, the BAG-1 RNA levels remained unchanged upon treatment (Figure 3C). To validate this result, two other ligands known to bind and stabilize DNA G4s were also tested: Phen-DC3 and TMPyP4 (60,61). However, on RNA G4 structures, the TMPyP4 ligand can exert opposite effects (either stabilize or destabilize) depending on the mRNA (62,63). As shown in Figure 3B, treatment with 10 μM of Phen-DC3 resulted in a 2-fold decrease in the protein isoform levels. In contrast, treatment with 2 μM of TMPyP4 had no effect on BAG-1 protein levels, indicating that this ligand may not stabilize this particular rG4. As observed with the cPDS treatment, the RNA levels remained unchanged following treatment with Phen-DC3 and TMPyP4 ligands (Figure 3C). Taken together, these assays confirmed the post-transcriptional regulation of BAG-1 isoforms expression. Indeed, stabilization with ligands of the rG4 located in the BAG-1 5′-UTR decreased the protein expression levels of the three downstream endogenous isoforms, independently of mRNA changes.

### Disruption of rG4 formation through mutations increases reporter gene expression

General stabilization of rG4s by small-molecule ligands resulted in a decrease in the expression levels of the endogenous BAG-1 protein isoforms. The next step was thus to directly modulate the BAG-1 rG4 using G-to-A mutations that abolish rG4 formation, and then measure the effect on both the RNA and protein levels.

**Figure 4.**
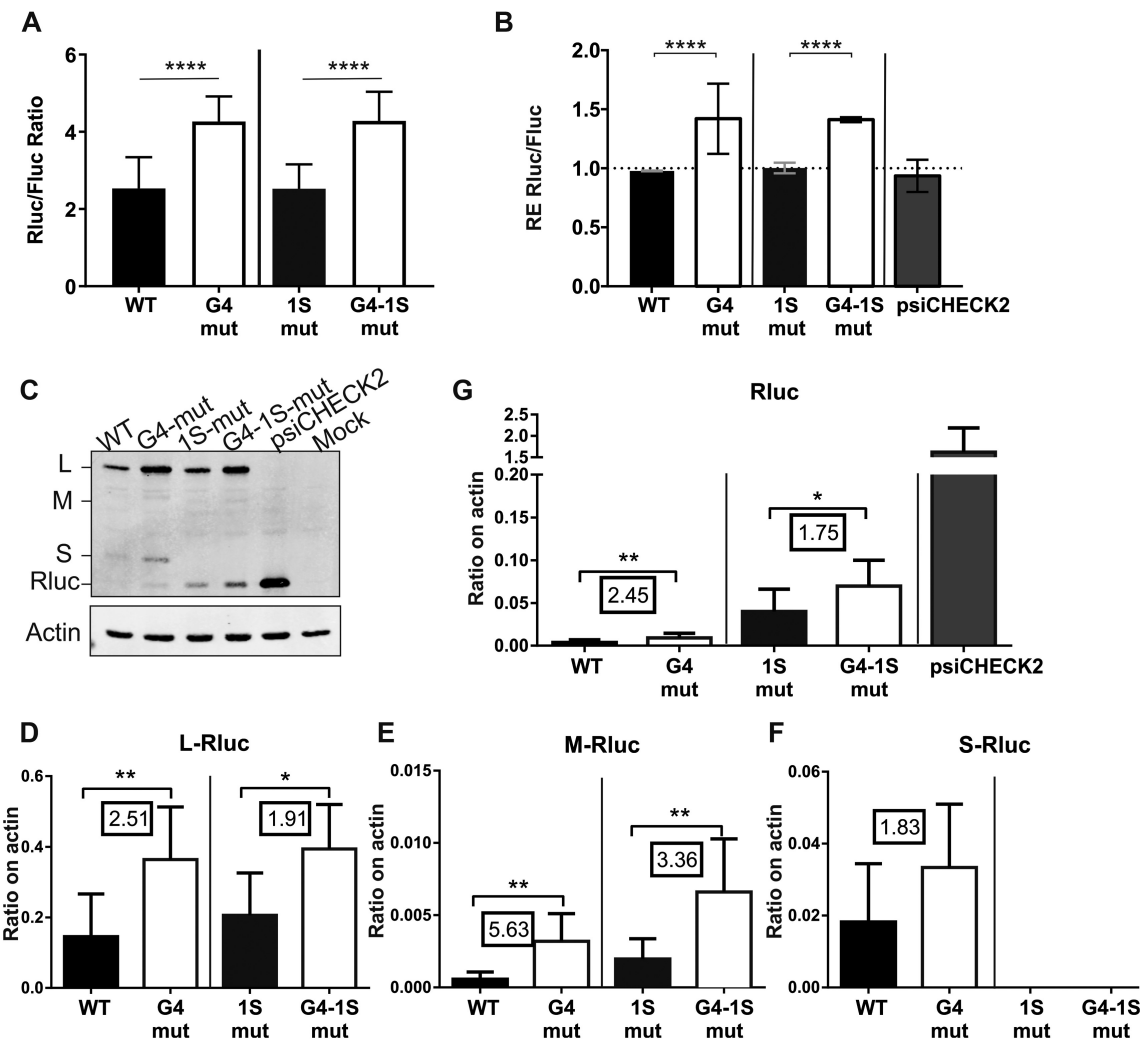
Luciferase, RNA and protein isoform expression levels of reporter assays of the complete 5′UTR of BAG-1 with both the mutated rG4 and the mutated 1S start codon. (**A**) The luciferase assays’ means and standard deviations of the Rluc luminescence levels, normalised over the Fluc luminescence levels, are shown for the WT (black) and rG4 mut (white) psiCHECK-2 constructions that included or not the 1S start codon mutation. The experiments were repeated three times with each of the constructions being transfected in triplicate (*n* = 3). The statistical analysis performed is a two-way ANOVA with Tukey's multiple comparison. ****P≤ 0.0001. (**B**) Relative expression (RE) levels of the Rluc RNA normalised over that of the Fluc RNA after the transfections of the different mutated constructions as measured by RT-qPCR. The bar of the RE level of the reporter plasmid without the insertion of the BAG-1 5′UTR is labeled psiCHECK-2. The statistical analysis performed was a two-way ANOVA with Tukey's multiple comparison test (*n* = 2). ****P≤ 0.0001. (**C**) Representative immunoblot of the Rluc N-extension protein isoforms’ expression levels after both the rG4 and 1S start codon mutations. The psiCHECK-2 transfection lane represents the canonical Rluc without any N-terminal extension. Mock indicates the untransfected control. β-actin was used as a loading control. (**D**-**G**) Quantification of the level of each isoform, normalised over that of the β-actin loading control, (**D**) L-Rluc (**E**) M-Rluc (**F**) S-Rluc (**G**) Rluc. The boxed values are the fold-change in the protein level of the rG4mut construction over that of the WT. The statistical analysis performed was a Mann-Whitney test (*n* = 3). *P≤ 0.05, **P≤ 0.001, ***P≤ 0.0005.

The most common method for assessing any effect of rG4 on the expression level of a given mRNA *in cellulo* is to insert the complete 5′UTR upstream of a luciferase reporter gene and to compare its expression level to that of a second construction in which the rG4 is mutated. Using this approach, we previously demonstrated that the abolition of the rG4 located in the BAG-1 5′UTR resulted in a 3-fold increase in luciferase expression in three CRC cell lines (30). However, only the 5′UTR region corresponding to positions 1 to 87 located upstream of the CUG start codon was used in that study. Herein, we performed the experiment with the complete 501 nts of the 5′UTR including all of the alternative start codons located downstream of the rG4. The nucleotide substitutions chosen were that of the purine G to another purine A in the rG4 G-tracts. Those substitutions were minimal, only seven mutations out of the 501 nts sequence, and did not create unwanted new sequence motifs such as an AUG start codon. With this complete 5′UTR construction, mutation of the rG4 resulted in a 1.7-fold increase in luciferase expression level, even when the 1S start codon coding for the most abundant of all three isoforms was mutated from AUG to AGG (Figure 4A).

### The rG4 affects the protein abundance of all N-terminal extension isoforms

The translation initiation at the three alternative start codons, all of which are in frame with the *Renilla* luciferase (Rluc) reporter gene in the complete 501 nts 5′UTR construction, could result in luciferase protein isoforms with alternative N-terminal extensions. These additions could thus alter the transcription of the DNA reporter and the resulting Rluc protein's folding and enzymatic activity. Consequently, in order to accurately measure the luciferase expression levels in the presence of the N-terminal extensions, immunoblots against the C-terminal region of the Rluc were performed along with RNA quantifications (Figure 4B and C).

As anticipated, the transfection of the complete 5′UTR reporter constructions resulted in the use of the alternative start codons for translation of the Rluc reporter. Rluc isoforms with N-terminal extensions that migrated at correspondingly higher molecular weights than the canonical Rluc were observed (Figure 4C). The canonical Rluc control (36 kDa) was seen in the psiCHECK-2 vector (Figure 4C, lane 5). The isoform L-Rluc was the most expressed from the BAG-1 WT 5′UTR reporter construct, while M-Rluc and S-Rluc are barely detected (Figure 4C). Of note, the ratios of the different isoforms of the Rluc reporter differ from the endogenous BAG-1 isoform ratio, with the L and S isoforms of the reporter being respectively more and less abundant than the endogenous protein. The reporter does not share the same 3′UTR sequence, coding sequence length and composition than the endogenous BAG-1 transcript. These elements are known to also affect translation initiation (64,65), and therefore might explain the different isoform ratio. However, the reporter allow to isolate the 5′UTR effect, and specifically the rG4 effect, on the translation of the different isoforms. The abolition of the rG4 resulted in an increased abundance of 1.8- up to 5.6-fold of all Rluc N-terminal extension isoforms, as well as of the shortest Rluc isoform that lacks the N-terminal extension, as compared with the WT protein levels normalised to the actin loading control (Figure 4D–G). Comparison to the level of the co-expressed Fluc protein is presented in Supplementary Figure S5.

In order to verify if rG4 formation affects all protein isoforms similarly, the rG4 mutation was individually combined with the mutation of each start codon. The rG4 effects were thus measured as the differences in the remaining possible isoform levels between the WT and the rG4 mutated constructions. The 1S-mut construction, in which the start codon of the S isoform was abolished by an AUG to AGG mutation, resulted in the loss of protein expression of that isoform in favor of the next start codon in the sequence, namely the canonical Rluc without N-terminal extension that mimics the shortest BAG-1 p29 isoform (Figure 4C, lane 3). The observed fold-increase in the protein isoform levels is similar between the reporter constructs pair (rG4-mut over WT), and the reporter constructs pair (1S-mut-rG4-mut over 1S-mut only) (Figure 4D–G). This demonstrated that even if the four isoforms are not equally expressed, the rG4 represses the translation of all of them to the same extent.

That said, the Rluc RNA levels were slightly different after transfection of the different constructions (Figure 4B). All constructions bearing the rG4 mutation had relative RNA expression levels 1.5-fold higher than the constructions with the intact rG4. This increase in the RNA levels was still lower than the average 2-fold increase in the protein levels that was observed for all of the rG4-mut constructions. Thus, the increase in the protein expression levels is not directly proportional to that of the RNA levels. The protein levels could also be increased by a more efficient translation of the rG4mut constructions. Both of these effects of the rG4 on the Rluc RNA and protein isoform levels were also observed using constructions in which either the 1L or the 1M start codons was mutated (Supplementary Figure S3). In summary, rG4 formation exerts a repressive effect on the protein expression levels of all in-frame protein isoforms.

This result is reminiscent of both the leaky scanning and the alternative translational initiation mechanisms. At the initiation step, the 43S ribosomal complex scans the 5′UTR until it recognizes one of the in-frame start codons by complementarity, a process that is favored by the strength of the initiation context. Because the rG4 is located upstream of all of the start codons, it impairs the scanning efficiency from the very beginning, before any ‘encounter’ with a potential start codon, thus affecting the translation of all the isoforms. The proximity of BAG-1 rG4 to the 5′-cap (position 6 specifically) might also contribute to its repressive effect on translation. Indeed, rG4 located close to the cap are more detrimental than those located further downstream (66) as they can impede either the co-transcriptional 5′ cap synthesis, or its recognition by the translation initiation factors. The BAG-1 rG4 is located very close to the 5′ end, specifically at position 6. However, we did not observe any difference between the WT and the rG4 mut BAG-1 sequences during both *in vitro* cap-synthesis assays and affinity binding assays with the cap-binding protein eIF4E (data not shown).

### A repressive uORF is present in the BAG-1 5′UTR

In addition to the presence of multiple in-frame start codons located downstream of the rG4, the BAG-1 5′UTR also possesses start codons in the other frames. One of them, the out-of-frame AUG located at position 254, stands out as it presents a more favorable context for translational initiation than all of the in-frame start codons (Supplementary Figure S4A and Supplementary Table S3). The analysis of publicly available ribosome profiling data (67–69) revealed the presence of ribosome-protected fragments (RPF) corresponding to initiating ribosomes at this position in different cell lines (Supplementary Figure S4B). A UGA stop codon is located downstream at position 302 of the 5′UTR, and it could result in a short open-reading frame (ORF) of 16 amino acids. The presence of a short out of frame ORF located upstream of the main protein coding sequence corresponds to the definition of an upstream ORF (uORF). uORF are *cis* regulatory elements that also repress translation. They act as decoys for the ribosomes in order to initiate translation early, before the main ORF. Their presence creates new requirements of translational re-initiation in order to translate the main ORF (70). Typical examples of repressive translation regulation by uORFs in 5′UTRs are the ATF4 and C/EBPα-β transcripts (71,72). Another example is the CAT-1 mRNA which also presents a 5′UTR organisation similar to that of the BAG-1 mRNA with the presence of an uORF located upstream of an IRES. The translation of the uORF in the CAT-1 5′UTR unfolds an inhibitory structure leading to IRES activation, a mechanism described as the ‘Zipper model’ (73). Therefore, the impact of this uncharacterized possible uORF on the BAG-1 regulation of translation was next investigated both in the presence and the absence of the rG4 structure.

**Figure 5.**
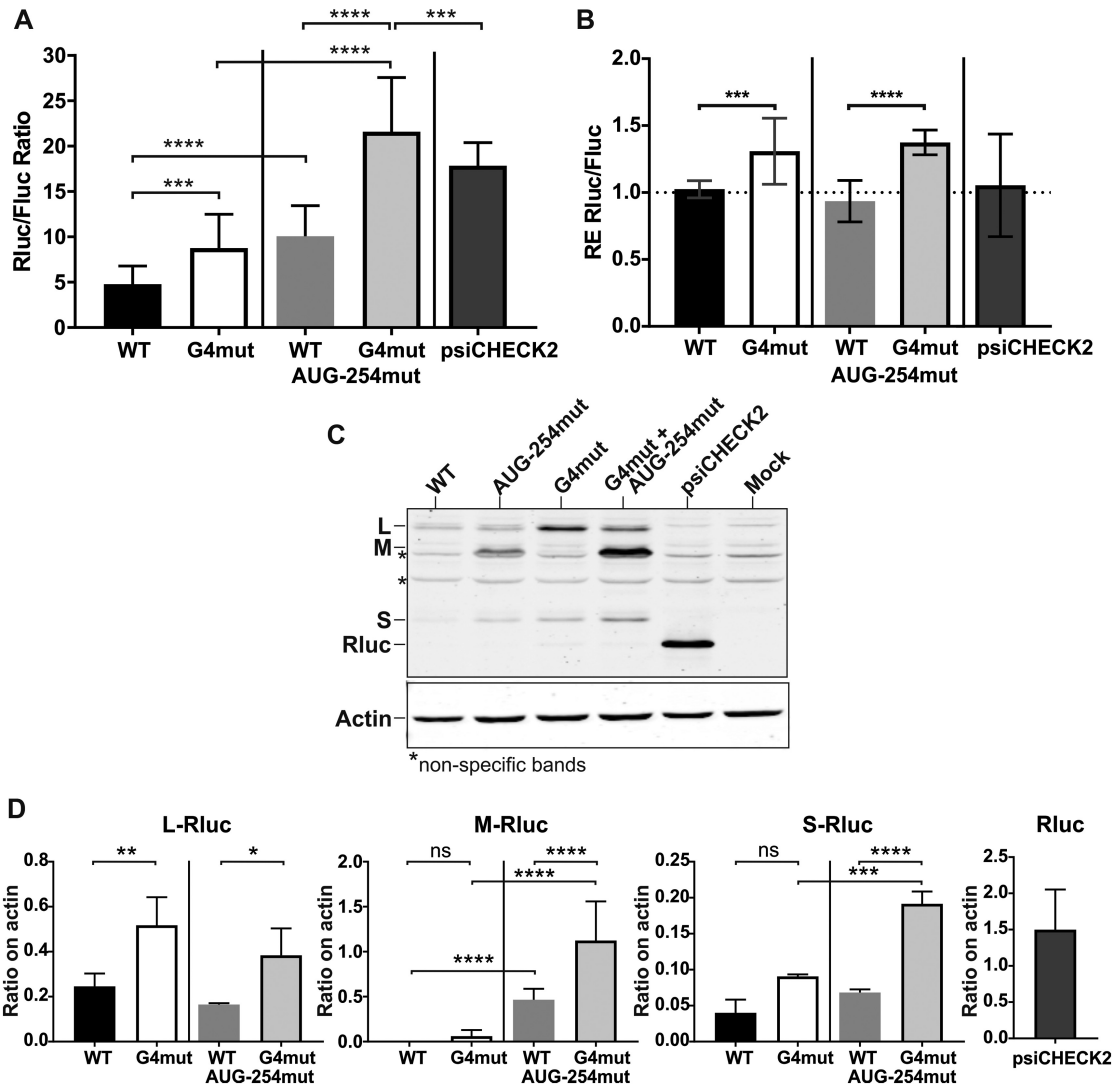
Luciferase, RNA and protein expression levels in reporter assays of the 5′UTR of BAG-1 possessing the mutated AUG-254. (**A**) The luciferase assays’ means and standard deviations of the Rluc luminescence levels, normalised over the Fluc luminescence levels, are shown for all constructions. The statistical analysis performed was a two-way ANOVA with Tukey's multiple comparison (*n* = 2, each construction was transfected in triplicate). ***P≤ 0.001, ****P≤0.0001. (**B**) Relative expression (RE) levels of the Rluc RNA normalised over that of the Fluc RNA after the transfections of the different mutated constructions as measured by RT-qPCR. The bar of the RE level of the reporter plasmid without the insertion of the BAG-1 5′UTR is labeled psiCHECK-2. The statistical analysis performed was a two-way ANOVA with Tukey's multiple comparison test (*n* = 2). ***P≤ 0.001, ****P≤ 0.0001. (**C**) Representative immunoblot of the Rluc N-extension protein isoforms expression levels after both the rG4 and the AUG-254 start codon mutations. The psiCHECK-2 transfection lane represents the canonical Rluc without any N-terminal extension. Mock indicates the untransfected control. β-actin was used as a loading control. (**D**) Quantification of the level of each isoform, normalised over that of the β-actin loading control. The statistical analysis performed was a two-way ANOVA with Tukey's multiple comparison (*n* = 2, each was construction transfected in triplicate). ns = not statistically significant, *P≤ 0.05, **P≤ 0.01, ***P≤ 0.001, ****P≤ 0.0001.

To first confirm whether or not the possible uORF affects the protein expression levels of BAG-1, the AUG located at position 254 was mutated to ACG in the reporter gene with the full-length BAG-1 5′UTR sequence in-frame with the Rluc coding sequence. This silent mutation was chosen in order to conserve the same histidine coding in the main frame of the Rluc N-terminal extension protein isoform, while completely disrupting both the start codon and the translation initiation context sequence of the possible uORF (Supplementary Table S3). The luciferase expression level of the mutated uORF construct was 2-fold higher than that of the WT 5′UTR construct (Figure 5A), indicating that this AUG-254 does act as a repressor element. The repressive effect is post-transcriptional as no difference was observed between the RNA levels of the WT and the AUG-254mut constructs (Figure 5B). At the protein level, the mutation of the AUG-254 increased the abundance of the M-Rluc isoform (Figure 5C, D). This was expected, as the AUG start codon of the M-isoform located at position 301 is the next one in line after the AUG-254 in the scanning of the 5′UTR. A slight decrease in the 1L isoform level was observed when the AUG-254 is mutated, but this difference is not statistically significant. The 1M- isoform possesses a stronger translational initiation context than does the 1L-isoform. Without the repressive AUG-254 start codon, the translation of downstream 1M isoform might be favored over the 1L. The level of the downstream 1S-isoform was also slightly increased upon the AUG-254 mutation, but the difference was statistically significant only when the rG4mut construct was compared to the combined rG4mut-AUG-254mut construct.

Notably, upon rG4 abolition, the mutation of the AUG-254 resulted in a doubled increase of the luciferase expression level as compared to that observed with the AUG-254 mutation alone (Figure 5A). The luciferase expression was even higher than that of the psiCHECK-2 reporter control without the inserted 5′UTR. This effect was also seen at the protein level (Figure 5C, D). As shown previously, the rG4 mutation resulted in an increased abundance of all of the isoforms. The combination of both rG4 and AUG-254 mutations also resulted in a doubled protein levels as compared to that of the AUG-254 mutation alone (Figure 5C, D). Therefore, as this is the case for the in-frame start codons, the rG4 seems to also repress the scanning during the very first steps of translational initiation, before the encounter with the repressive uORF, affecting the initiation at this out-of-frame AUG in a manner similar to that seen at the in-frame start codons.

One could speculate that this newly characterized uORF regulates the BAG-1M isoform. Indeed, this isoform was less expressed than the 1L isoform despite its canonical start codon. Because of its more favorable Kozak context, initiation is very likely favored at AUG-254 rather than at the BAG-1M start codon. Furthermore, the 1M start codon located at position 301 is hidden inside the uORF sequence, limiting the chances of re-initiation and thus reducing its expression. Under stress conditions, where re-initiation is slowed down due to the reduced availability of both the ternary complex and the initiation factors, both the BAG-1M translation, as well as the BAG-1S translation with the start codon situated further downstream at position 501, might be favored. This is a mechanism that is common to other transcripts that possess alternative in-frame start codons along with uORFs (74). However, this remains to be validated experimentally for BAG-1. It is still unknown if the uORF located at position 254 is readily translated into a short peptide, or if it only functions to divert the pre-initiating ribosome complex from the main reading frame of the BAG-1 isoforms.

The BAG-1 5′UTR possesses thus two *cis*-elements that affect the cap-dependent translation: an rG4 and an uORF which both repress translation of the protein isoforms with N-terminal extensions. Recent work by the Balasubramanian group demonstrated that, at the genome level, rG4s are enriched in 5′UTRs with possible repressive uORFs, and that they can stimulate translational initiation at these uORFs (75). The case of BAG-1 5′UTR is however different from their proposed model since the BAG-1 rG4 is located upstream of the uORF instead of downstream. Nevertheless, similarly to Balasubramanian *et al.* (75), our data indicate that when rG4 and uORF are both present, they exert a stronger repressive effect on translation than the presence of only one of them does.

### Disruption of the rG4 formation is detrimental to BAG-1 expression in a bicistronic context

Disruption of the rG4 formation in the BAG-1 5′UTR probably facilitated the scanning of the 5′UTR, hence impacting the alternative translational initiation of the three principal protein isoforms. However, leaky scanning and alternative translational initiation are not the only mechanisms regulating the translation of the BAG-1 mRNA. The identification of a putative uORF suggests that translation re-initiation could take place. In addition, the BAG-1 5′UTR possesses an IRES secondary structure (15). With the collaboration of ITAFs, the secondary structure allows for remodelling of the IRES structure, and the direct recruitment of the 40S ribosomal subunit at the RBS to initiate translation in a cap-independent manner (27). Some rG4 prone sequences were previously found to affect both the folding of the secondary structure and the cap-independent translation of IRES (40,41). Therefore, the possible impact of the BAG-1 rG4 on the IRES-driven translation was investigated.

**Figure 6.**
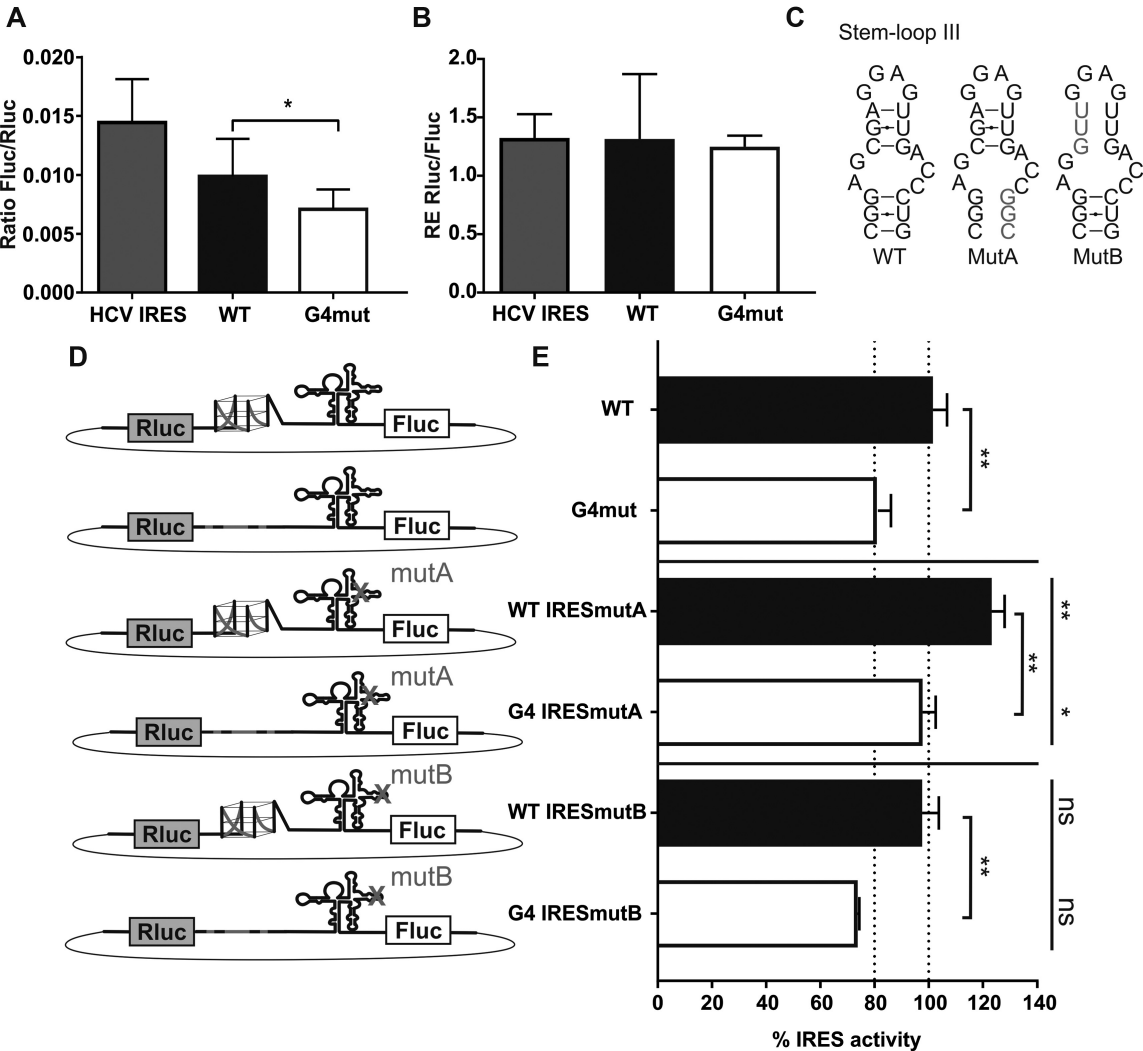
rG4 mutation impairs the cap-independent translation of the BAG-1 IRES. (**A**) The ratios of Fluc/Rluc luciferase levels following transfection of the bicistronic plasmid construct are shown. The HCV IRES (gray) bicistronic construct was used as a positive control, with the well characterized IRES placed upstream of Fluc. The WT (black) represent the bicistronic construct with the full length 5′UTR of BAG-1 located upstream of the Fluc. The G4mut (white) represents the bicistronic construct with the full-length 5′UTR of BAG-1 that included the G-to-A mutations abolishing the folding of the rG4. For each experiment, all constructions were transfected in triplicate. The results presented are the means and standard deviations of *n* = 5 independent experiments. The statistical analysis performed was a paired *t*-test. *P≤ 0.05. (**B**) The ratios of the relative RNA expression levels of Rluc and Fluc following transfection. The ratios are close to 1 and are similar between the three constructs, demonstrating the integrity of the bicistronic construct. The bars indicate the means and standard deviations of *n* = 3. (**C**) Representation of the WT Stem-loop III secondary structure as defined by Pickering *et al.* (27), and that of the IRESmutA containing the GUC to GCC mutation at positions 367–369 and the IRESmutB containing the CGA to GUU mutation at positions 354–356. (**D**) Schematic representations of the bicistronic plasmid constructions with the various rG4 and IRES structure mutations used in the assays. (**E**) Percentage of IRES activity for each construct. The 100% activity level was defined as the Fluc/Rluc ratio of the WT construct. WT constructions in which the rG4 is intact are in black, while the rG4mut constructions, in which the rG4 is abolished by G/A-mutations, are in white. The bars represent the means of two assays (*n* = 2), each sample was transfected in triplicate, and the error bars represent the standard deviations. The top horizontal bar represents the statistical significance as compared to the IRESwt constructions (WT or G4mut, respectively). The statistical analysis performed was a one-way ANOVA with Tukey's multiple comparisons *P≤ 0.05, **P≤ 0.01.

In order to detect both IRES-dependent translation and re-initiation, we employed bicistronic luciferase reporter DNA backbone vectors. In these constructions, the complete 501 nts 5′UTR of BAG-1 was inserted between the Rluc and the Fluc reporter genes. The Rluc is expressed following cap-dependent translation, while the Fluc is translated following cap-independent internal initiation of translation or by re-initiation. The Fluc/Rluc ratio thus represents the bicistronic activity. Again, the WT BAG-1 5′UTR was compared to the rG4 mutant in order to observe the difference in the Fluc normalised expression levels. The well-characterized IRES from Hepatitis C virus (HCV), specifically the initial pRL-HL construction, was used as a positive control for the IRES-dependent translation (Figure 6A). In opposition to the monocistronic luciferase construction for which the rG4 mutation triggered a higher luciferase expression, the transfection of the rG4 mutated bicistronic construction produced a small, but consistent, decrease of 20% in the Fluc/Rluc ratio (Figure 6A). The effect is translational since no difference was seen in the RNA expression levels of the constructions (Figure 6B). The absence of monocistronic Fluc products resulting either from cryptic promoter usage, or unexpected splicing, was confirmed by Northern blot analyses using both Rluc and Fluc specific probes (Supplementary Figure S6).

A decrease in the bicistronic-activity of only 20% was considered as low, so comparisons of this reduction with those of the other mutations known to affect the BAG-1 IRES activity were performed. Pickering *et al.* (27) deciphered the secondary structure of the minimal IRES region of BAG-1 (corresponding to positions 247 to 432 of the 5′UTR) and identified the stem-loop III as being essential for both the recruitment of ITAFs and the IRES-dependent translation (Figure 6C). In their work, the mutation of either the bottom part of the stem-loop III (MutA), or the upper part (MutB), significantly reduced the IRES activity in an *in vitro* translation assay using rabbit reticulocyte lysates. Those mutations were thus added to the bicistronic constructions and compared to both the BAG-1 WT and the rG4 mutant (Figure 6D). Surprisingly, the introduction of these IRES mutations in either the WT or the rG4mut bicistronic constructions did not reduce the bicistronic activity. The IRESmutB presented a bicistronic activity identical to that of the WT construction, while the IRESmutA resulted in a 20% increase in the bicistronic activity (Figure 6E). Independently of the presence of either IRESmutA or IRESmutB, the rG4 mutation still resulted in a 20% decrease in the bicistronic activity as compared to that of the corresponding intact rG4 construction.

The discrepancies in the bicistronic activity levels following the Stem-loop III mutations observed in both the initial work of Pickering *et al.* and our work could be explained by the different translational systems used, specifically rabbit reticulocyte lysates initially and the transfection in HCT116 CRC cells here. Furthermore, the initial sequence for the IRES secondary structure determination and translation assays did not include the nucleotides of the rG4 region (positions 6 to 35). Hence, it is possible that the rG4 secondary structure folding impacts the global secondary structure folding of the 5′UTR, influences secondary structure long-range interactions, or triggers the folding of an alternative secondary structure that could then affect the IRES efficiency and mitigate the IRES mutations A and B. Although this assay did not provide a complete negative control of IRES activity for comparison, it did demonstrate that the 20% decrease in the expression of the DNA bicistronic luciferase transfection assay was reproducible. Therefore, the abolition of the rG4 affected the bicistronic-dependent translation activity in a lesser, but opposite, way compared to that of the cap-dependent translation observed using monocistronic constructs.

### Cap-dependent translation is the main translational mechanism of the BAG-1 5′UTR under normal growth conditions

The DNA transfection of the monocistronic luciferase construct containing the complete BAG-1 5′UTR demonstrated that the rG4 repressed expression because its abolition increased the amount of luciferase protein (Figure 4C). In this assay, the mRNA levels of the rG4mut constructs were also barely increased in comparison to the constructions with the intact rG4 region (Figure 4B). In the DNA transfections of the bicistronic constructs, in which the complete 5′UTR was located between the two luciferases, the rG4 had the opposite effect: its abolition consistently resulted in a 20% decrease of the IRES activity (Figure 6E). Multiple controls were performed in order to eliminate the possibility of artifacts resulting from *in cellulo* modifications of the bicistronic DNA construct after transfection. Nevertheless, to limit the differences observed in the RNA levels between the WT and rG4mut monocistronic constructions in the initial transfections, and to directly account for differences at the translational level, the luciferase reporter assays were repeated using the direct transfection of exact amounts of monocistronic and bicistronic capped and poly-adenylated luciferase reporter mRNAs. Furthermore, the luciferase activity was normalized on the RNA levels post-transfection using the reverse transcription of the total RNA extracts and the ddPCR quantification of the resulting cDNA for both the monocistronic and bicistronic mRNA constructs.

All of the mRNA construct templates were created using the BAG-1 5′UTR bicistronic DNA vector and different sets of primers (see Methods). The resulting templates were then transcribed *in vitro*, capped with either the canonical m^7^G-cap or the A-cap analog, and then polyadenylated. The monocistronic mRNA constructs bearing the BAG-1 5′UTR upstream of the Fluc reporter coding sequence were co-transfected with the Rluc monocistronic control (Supplementary Figure S7A). In order to obtain the translation level of each construct, the Fluc expression level was normalised over the Rluc expression level (Fluc/Rluc ratio) for each construction, either mono- or bicistronic, and was corrected by the corresponding ratio of the Fluc/Rluc RNA levels as measured by RT-ddPCR from the same transfected cell lysate. The results are presented relative to the translation level of the WT monocistronic construct which was set to 100% (Supplementary Figure S7B).

The monocistronic mRNA transfection reproduced the effect of the rG4 observed in the first DNA transfection luciferase assay: the mutation of the rG4 resulted in an increase in translation (Supplementary Figure S7B). In the presence of the A-cap analog, which controlled the 5′ end-dependent but not the m^7^G-independent translation, the translation level of the WT 5′UTR was found to be significantly lower at 9.5% of the m^7^G-dependent translation. The mutation of the rG4 seemed to increase translation up to 19%, but this was not statistically significant due to the low translation level. This indicates that the rG4 could repress both the m^7^G- and 5′ end-dependent translational mechanisms. For the bicistronic mRNAs, no difference in the translational levels was observed between the WT and the rG4mut, with the translation levels corresponding to 15.8% and 17.5% of those of the cap-dependent translation, respectively. The decrease in the IRES activity upon rG4 mutation was not observed in this case. In this assay, the bicistronic translation levels were so low, as compared to that observed with the m^7^G-cap monocistronic mRNAs, that a 20% reduction could be impossible to detect. Another explanation could be the difference between RNA and DNA transfection on the IRES functionality. It is known that some IRES require a ‘nuclear experience’ to be fully functional. The nuclear localisation of the RNA bearing the IRES might be essential for either the modification of the mRNA by methylation or pseudouridylation, or to recruit essential ITAFs that are located primarily in the nucleus (76,77). Caution is thus needed in the interpretation of the BAG-1 IRES activity upon mRNA transfection because it might not exactly reflect the endogenous conditions of the mRNA transcribed in the nucleus.

By comparing the Rluc/Fluc expression levels of the different monocistronic and bicistronic mRNA constructions used here, it seems that the dominant translation mechanism of the BAG-1 isoforms is cap-dependent under the normal HCT116 growth conditions used. This is consistent with previous studies that indicated that the IRES-dependent translation occurs under stress conditions (15,28). However, the initially observed 20% repression of the bicistronic-dependent translation that occurs upon DNA transfection when the rG4 is mutated could be explained by the impact of the rG4 on the global 5′UTR folding affecting the stability of key subdomains of the IRES secondary structure.

### Formation of the rG4 affects the global secondary structure of the 5′UTR

Stable secondary structures located near the m^7^G-cap are known to impede both the scanning of the ribosome and the initiation of translation (78). Furthermore, structural accessibility of the regions surrounding the start codons also affects the translational efficiency, and can influence the leaky scanning mechanism (79). The cap-independent translation mechanism is also very dependent on the accurate secondary structure folding of the IRES. The secondary structure folding of the 5′UTR is thus important for both types of translational initiation, and the impact of the rG4 on the global 5′UTR secondary structure might explain its apparent opposite effects on the cap-dependent and -independent translation mechanisms.

In order to investigate whether or not the rG4 abolition could affect the global 5′UTR folding, and more specifically the secondary structure surrounding each of the start codons and the IRES secondary structure, selective 2′-hydroxyl acylation analyzed by primer extension (SHAPE) was performed on the complete WT, the rG4mut and the IRESmutA BAG-1 5′UTR. In each construction, a 40-nts extension was added to the 3′end of the 501 nts *in vitro* transcribed RNA 5′UTR in order to allow for the primer binding required for reverse transcription. A second primer, binding in the middle part of the UTR (positions 301 to 320) was also used in the primer extension step to optimize the reverse-transcriptase coverage of the 501 nts. The cDNAs were then analyzed by capillary electrophoresis. Flexible nucleotides from the secondary structure are more prone to react with the acylating SHAPE reagent, creating more stops at those positions during primer extension. The averaged reactivity of each nucleotide, from two independent SHAPE experiments from each primer, was used as pseudo-energy constraint in order to predict the secondary structure using the RNAstructure algorithm (48). This software cannot predict rG4 secondary structures. Hence, in order to avoid base-pair predictions for the guanines of the G-tracts elsewhere in the UTR, predictions were concomitantly performed with and without the constraint that the nucleotides located in positions 1 to 35 remain single-stranded (G4ss). This constraint was also used for the G4 mutated sequences. All secondary structures, with or without the G4ss constraint, were then compared. Up to 18 possible secondary structures respecting either the SHAPE pseudo-energy constraint, or the G4ss and SHAPE constraints, were obtained for each 5′UTR WT and mutated sequence (Table 1).

**Table 1.** Number of secondary structure predictions generated by RNAstructure for each of the mutated sequences using the SHAPE pseudo-energy constraints

	Number of secondary structure predictions		
Sequences	Pseudo-energy constraints only	Pseudo-energy constraints + G4ss^a^	Total
**WT**	8	17	25
**G4mut**	12	14	26
**WT IRESmutA**	16	13	29
**G4mut IRESmutA**	16	18	34
**Total**	52	62	114

^a^G4ss represent secondary structure predictions in which the G4 region was constrained to stay single stranded

**Figure 7.**
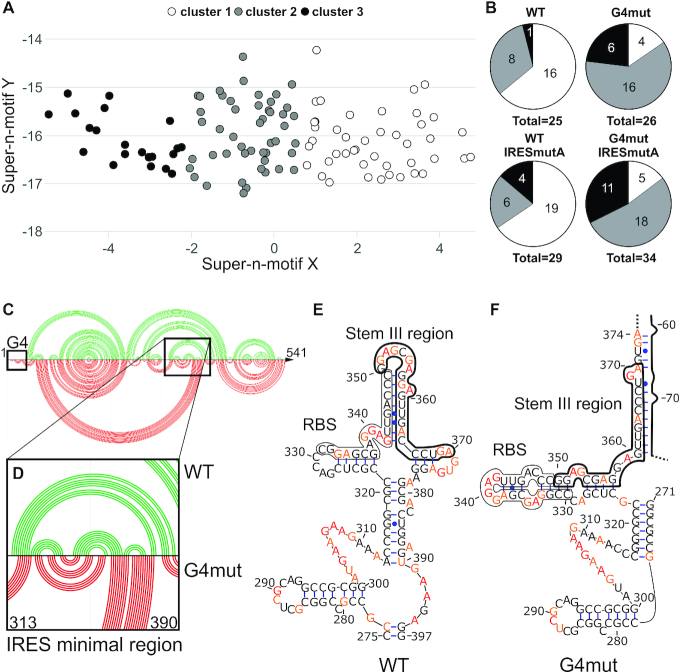
Effects of both the rG4 and IRES mutations on the global secondary structure of the BAG-1 5′UTR, as analyzed by SHAPE. (**A**) Super-n-motif representation of the 114 predicted secondary structures, separated into three clusters (white, cluster 1; gray, cluster 2; black, cluster 3). (**B**) Distribution in the three clusters of the predicted secondary structure of every sequences analyzed. (**C**) Arc-plot representation of the most stable predicted secondary structure of the complete WT (G4ss) sequence (green) compared to that of the rG4mut sequence (red). The rG4 region is boxed. (**D**) Close up of the arc-plot secondary structure of the IRES minimal region from nucleotide positions 313 to 390 (E, F). Most stable secondary structure of the minimal IRES region of the (**E**) WT (G4ss) sequence, and (**F**) rG4mut sequence. The color of the nucleotide represents its normalised SHAPE reactivity: black non-reactive; yellow, reactive; and, red, highly reactive. (*n* = 2 for each of the two primers). Both the RBS and the Stem III region are boxed.

The StructureXplor software was then used to compare and cluster similar secondary structures (80). This software uses the combinations of short secondary structure motifs (Super-n-motif), instead of sequence alignment, to assess secondary structure similarities. Thus, it can compare secondary structures obtained from different mutated sequences (81). The ensemble of the possible predicted structures from the WT, the rG4mut and the IRESmutA 5′UTR sequences could be separated into three distinct secondary structure clusters of different sizes, with the cluster 3 containing less structures than clusters 1 and 2 (Figure 7A). The quality of the clustering was evaluated using the computed silhouette coefficients (possible values from –1 to 1, 1 being the highest clustering quality), which were of 0.616; 0.772; 0.711 for clusters 1 to 3, respectively. This signifies that the secondary structures are similar within each cluster, and that they are well-differentiated between the different clusters. Of note, the secondary structures of the WT and the rG4mut sequences were not uniformly distributed in the 3 clusters. The predicted structures of the WT sequences with or without the G4ss constraint are mostly found in cluster 1, while the predicted structures of the rG4mut sequences with or without the G4ss constraint are found in cluster 2 (Figure 7B, top). This demonstrated that first, the G4ss constraint did not affect the clustering as the secondary structures with this constraint are not all retrieved in the same cluster. Second, it demonstrated that globally, the ensemble of predicted secondary structures obtained is different between the WT and the rG4mut sequences. The abolition of the rG4 folding results in the alteration of the global secondary structure folding of the 5′UTR. However, the IRES mutation A does not affect the global folding, as sequences bearing this mutation are clustered in the same proportions as are the WT or the rG4mutation alone (Figure 7B, bottom).

### The stability of the structural subdomains of the 5′UTR is affected by rG4 formation

In order to evaluate whether or not the rG4 folding affects the secondary structure surrounding either the start codons or the IRES subdomain of the 5′UTR, the most stable predicted secondary structures, based on the SHAPE reactivity constraints for both the WT and the rG4mut sequences, were compared in detail. The base-pairing, excluding the rG4 pairing, was represented using an arc-plot (Figure 7C). Of the 163 and 175 bp of the WT and rG4mut structures, respectively, 78 bp were identical. This represented 48% of the WT and 45% of the rG4mut total base-pairs, and they are shown as mirror images on the Arc-plot.

Stronger base-pairing and higher unfolding energies surrounding start codons are associated with less efficient translational initiation (79). If the rG4 disruption resulted in the generation of more relaxed structural states for the start codons it could explain how protein synthesis is augmented in the rG4 mutant. However, no significant differences in the structures around the start codon regions could explain the change in the expression levels between the rG4 and the WT sequences, as all of the in-frame start codons, and even the uORF AUG-254, were in similarly accessible secondary structures. The base-pairing differences occur mostly in the middle region of the 5′UTR. Secondary structure representations of that region for each sequence (WT, rG4mut, WT-IRESmutA and rG4mut-IRESmutA) are presented in Supplementary Figure S8. Interestingly, this region (Figure 7D) corresponds to the previously characterised IRES structure (27). The secondary structure of the IRES region predicted here differs from that of the previous work mostly by a shift in the binding of the Stem III nucleotides, and by globally having more base-pairing (Supplementary Figure S9). However, the most flagrant alteration upon rG4 abolition was the long-range base-pairing of the nucleotides of the IRES regions, located at positions 360 to 380, with the nucleotides from positions 55 to 77 instead of the intrinsic folding observed for the WT sequence (Figure 7E and F, WT and rG4mut, respectively). This change results in a sliding offset in the base-pairs from the identified RBS and Stem III regions, and affects the stability of both the previously defined Stem III region and the adjacent RBS. Evaluation of the changes, in terms of minimum free energy (MFE) as measured by the RNAeval tool of the Vienna RNA package (50), illustrated the differences in the predicted stabilities of these domains between the various mutated sequences (Table 2). Globally, there was no difference in the stability of the complete 5′UTR secondary structure, with the MFE ranging from –237.2 to –234.1 kcal/mol. However, the minimal IRES subdomain was more stable in the rG4mut folding (–105.7 kcal/mol) as compared to the WT (–74.0 kcal/mol). The disruption of the rG4 thus seems to shift the folding, making the IRES minimal region more stable. Based on the proposed mechanism of the IRES regulation of BAG-1 (27), a more ‘closed’ structure might be more difficult to unfold and therefore impede the binding of the ITAFs that are essential for the recruitment of the 40S ribosomal subunit. Thus, the 20% decrease in bicistronic activity observed for the rG4mutant can be explained by the impact of the rG4′s absence on the IRES subdomains secondary structures which in turn, affects the IRES-dependent translation. An interesting perspective would be to measure the binding affinity of the ITAF depending on the global 5′UTR secondary structure. Our results show that rG4 are not only steric blocks that repress translation. They can also modulate translation by promoting or preventing the formation RNA structural subdomains that contribute to translational regulation.

**Table 2.** Predicted minimum free energies (MFE) of the most stable secondary structures predicted by SHAPE for each mutant and region of the 5′UTR

	Minimum free energy (kcal/mol)			
Sequence region	WT	rG4mut	WT IRESmutA	rG4mut IRESmutA
**Complete 5′UTR**	–236.07	–234.06	–236.07	–237.20
**Minimal IRES**	–74.00	–105.70	–71.10	–100.30
**Stem–loop III**	–8.10 (11 bp)	–26.20 (16 bp)	–24.00 (13 bp)	–28.00 (15 bp)
**RBS**	–14.40 (13 bp)	–7.40 (7 bp)	–7.40 (7 bp)	–7.40 (7 bp)

## CONCLUSION

The BAG-1 protein isoforms are anti-apoptotic proteins which are overexpressed in CRC and associated with a poor prognosis. In this work, we demonstrated that the expression of BAG-1 protein isoforms is controlled at a post-transcriptional level in colorectal cells and in tumors. By using the CRC model cell line HCT116, we showed that the expression of the three main BAG-1 protein isoforms can be repressed by small molecule ligands targeting the rG4 structure of the BAG-1 mRNA. Importantly, this rG4 is localized upstream of several *cis-*regulatory elements in the 5′UTR including some alternative start codons, a non-canonical CUG start codon, an IRES and a putative repressive uORF. In this regard, we confirmed that BAG-1 rG4 represses the dominant cap-dependent translation of the three main protein isoforms, as previously observed with other 5′UTR rG4s (39). Additionally, rG4 mutation clearly inhibits IRES-dependent translation even though the rG4 it is not by itself a structural part of the IRES domain, contrary to the rG4s present in the VEGF and FGF-2 mRNAs (40,41). The rG4 disruption by key G-to-A mutations triggered a shift in the secondary structure of the IRES subdomain located 300 nts away in the 5′UTR.

Taken together, our data suggest a new mode of translational regulation by the BAG-1 rG4. Indeed, by imposing a specific conformation of the 5′UTR, BAG-1 rG4 modulates different types of translation. Along with the repressive uORF, it acts as a roadblock that interferes with both the scanning and the translation of all in-frame isoforms. It also acts as a structural scaffold by maintaining the global folding of the 5′UTR as well as the folding of its internal subdomains including the IRES secondary structure that is essential for translation under stress conditions. The BAG-1 rG4 is thus the first characterized rG4 with functions in both cap-dependent and independent translation.

A proteogenomic analysis previously demonstrated that mRNA abundance is not a good predictor of protein abundance in colonic and rectal tumors (56). Likewise, recent studies have highlighted the altered regulation of translation in various cancers (82), including CRC (83). Indeed, alternative mechanisms of translation, such as leaky scanning, re-initiation and IRES usage seem to be favored, and this has been associated with, higher cell proliferation, invasion and resistance to apoptosis and therapy (84). In this regard, the expression of BAG-1, recently described as a colorectal anti-apoptotic oncogene, is regulated by alternative translation mechanisms, and thus represents a good model to study how a rG4, along with the different regulatory elements of the 5′UTR, controls protein synthesis. The translational repression of specific mRNAs by the use of small molecules targeting the rG4s located in the 5′UTR have been demonstrated (85). Deciphering more examples like the rG4 of the BAG-1 mRNA 5′UTR could represent future avenues for therapies, as well as a better understanding of the mechanisms of action of rG4 on the translation regulation of other mRNAs possessing similar organisation of their 5′UTR. In the future, it might be interesting to study the impact of BAG-1 rG4 in a larger natural context, i.e. in the presence of its 3′UTR. It is known that 3′UTRs can greatly influence translation at several levels and that there is some communication between the 5′ and 3′UTRs (86). So it is not excluded that this type of interaction may exist with the 3′UTR BAG-1 mRNA and that this also influences the initiation of translation that occurs at the 5′end.

## Supplementary Material

gkz777_Supplemental_File
