# Supplementary material for: G-quadruplex located in the 5′UTR of the BAG-1 mRNA affects both its cap-dependent and cap-independent translation through global secondary structure maintenance
Source: Nucleic Acids Res. 2019 Sep 4;47(19):10247–66. doi: 10.1093/nar/gkz777 (PMC6821271; doi:10.1093/nar/gkz777)
Supplement: gkz777_Supplemental_File [file gkz777_supplemental_file.pdf]

Supplementary Data

G-quadruplex located in the 5'UTR of the BAG-1 mRNA affects both its cap-dependent and cap-independent translation through global secondary structure maintenance

Rachel Jodoin[1], Julie Carrier[2], Nathalie Rivard[3], Martin Bisaillon[1] and Jean-Pierre Perreault[1,*]

[1]Département de Biochimie, Faculté de médecine et des sciences de la santé, Université de Sherbrooke, Sherbrooke, Québec, J1E 4K8, Canada

[2]Service de Gastro-entérologie, Département de Médecine, Faculté de médecine et des sciences de la santé, Université de Sherbrooke, Sherbrooke, Québec, J1H 5N4, Canada

[3]Département d'Anatomie et de Biologie Cellulaire, Faculté de médecine et des sciences de la santé, Université de Sherbrooke, Sherbrooke, Québec, J1E 4K8, Canada

Table of content

Supplementary Material and Methods 3

BAG-1 endogenous RNA levels in CRC cell lines 3

Western blot of endogenous BAG-1 in CRC cell lysates 3

Supplementary Figures and Legends 4

Supplementary Figure S1. BAG-1 protein isoforms' expression levels in the supplementary paired tissues samples of colorectal tumors at different stages and their adjacent healthy tissue (margin). 4

Supplementary Figure S2. BAG-1 mRNA and protein isoforms expression levels in normal intestinal epithelial and CRC cell lines. 5

Supplementary Figure S3. RNA and protein isoforms expression levels of the reporter assays of the complete 5'UTR of BAG-1 with both the mutated rG4 and the mutated 1L or 1M start codons. 6

Supplementary Figure S4. The BAG-1 5'UTR possesses a repressive uORF located at position 254. 7

Supplementary Figure S5. Comparison of Rluc/Fluc expression ratios of the different BAG-1 5'UTR reporter constructs at the luciferase, RNA and protein levels upon transfection of HCT116 cells. 8

Supplementary Figure S6. Control of the bicistronic constructions integrity. 10

Supplementary Figure S7. Effects of the rG4 on both the cap-dependent and the cap-independent translation of the transfected mRNA reporter constructions. 11

Supplementary Figure S8. Secondary structure elucidated by SHAPE of the minimal IRES region of the BAG-1 5'UTR. 12

Supplementary Figure S9. Comparison of the IRES secondary structure elucidated by SHAPE using the WT complete BAG-1 5'UTR sequence with the structure elucidated by Pickering *et al.* 2004. 13

Supplementary Tables 14

Supplementary Table S1. Clinicopathological parameters of the CRC patients. 14

Supplementary Table S2. RNA and protein expression levels for each tissue pair presented as fold change of tumor expression level over margin expression level 15

Supplementary Table S3. Translation initiation efficiency of the start codons of the BAG-1 5'UTR. 17

Supplementary Table S4. List of primers and oligonucleotides used in this study. 18

Supplementary Table S5. Sequences of all of the 5'UTRs tested, those of the rG4 and the start codons that were mutated, the SHAPE WT and the mutated sequences. 21

Supplementary Table S6. Sequences of the transfected mono- and bicistronic mRNAs... 25

Supplementary References 37

Supplementary Material and Methods

BAG-1 endogenous RNA levels in CRC cell lines

The cDNAs resulting from the reverse-transcription (RT) of the total RNA extracted from diverse normal and cancerous colorectal cell lines were obtained from the J. Carrier biobank. The qPCR reactions were performed by the RNomics Platform as described in the main manuscript.

Western blot of endogenous BAG-1 in CRC cell lysates

Pooled protein lysates (10 µg) of the colorectal cell lines (HIEC, HCT116, CACO-2/15, SW48, DLD-1, HT-29, Colo205 and SNU) cultured under serum starvation condition were loaded on a 10 % SDS-PAGE gel which was migrated for 2 h 15 min at 150 V and transferred for 1 h at 100 V on a polyvinylidene difluoride (PVDF) membrane which was then blocked 15 min at room temperature in phoshate buffered saline (PBS) with 4% (w/v) nonfat drymilk (PBS-milk 4 %). The Western blot used to detect the BAG-1 protein isoforms was performed as described in the main manuscript, with the exception that β-actin was used as the loading control. After stripping of the membrane in 0.5 N NaOH twice for 10 min, and a thorough washing in PBS, the membrane was blocked in PBS-milk 4 % for 15 min and then incubated for 1 h at room temperature with the primary antibody, mouse anti-β-actin (AS441, Sigma), diluted 1: 1000 in the blocking buffer. After washes in PBS-T, the membrane was incubated for 1 h at room temperature with the secondary antibody, anti-mouse IgG (H+L) (IRDye 800CW, Li-Cor), diluted 1:10 000 in PBS-Milk 4 %. After 3 washes with PBS-T, the membrane was revealed using the Li-Cor Odyssey system.

Supplementary Figures and Legends

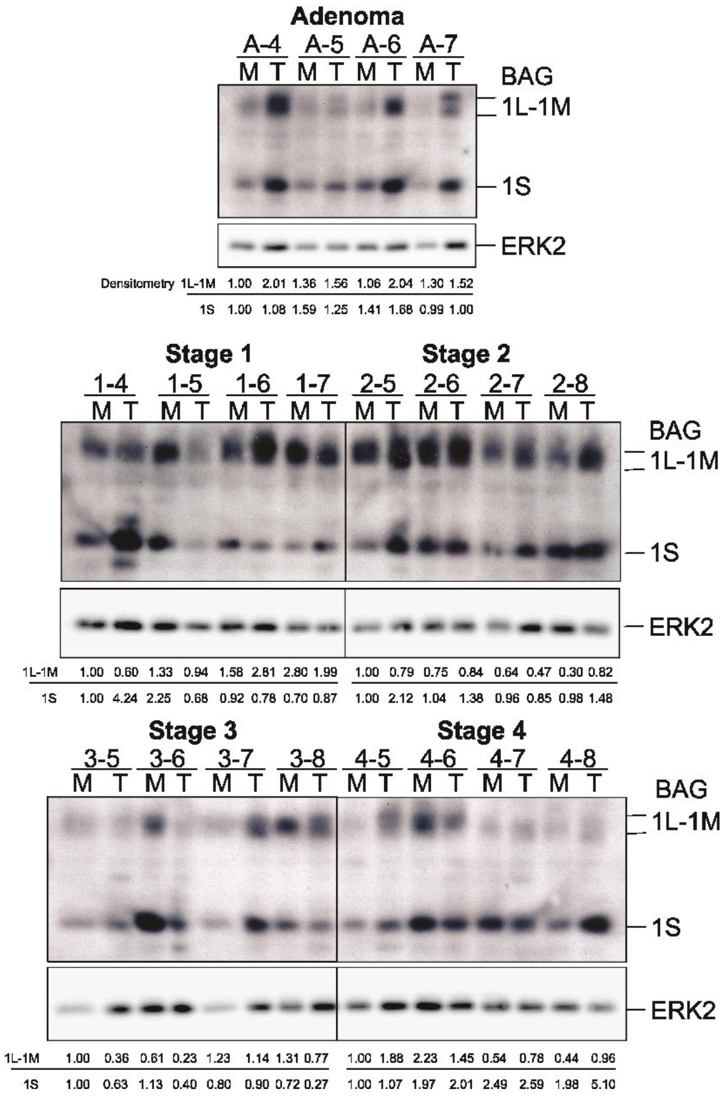

Supplementary Figure S1. BAG-1 protein isoforms' expression levels in the supplementary paired tissues samples of colorectal tumors at different stages and their adjacent healthy tissue (margin).

Protein expression levels, as measured by Western blot, of the three BAG-1 isoforms in the same pairs of margin (M)-tumor tissues (T) as in (**Figure 2A**). ERK2 is used as the loading control. The relative densities of the 2 most abundant isoforms, BAG-1L-1M and BAG-1S, are annotated under each lane. The isoforms band densities were corrected on the corresponding ERK2 loading control density and reported relative to the density of the isoform of the first lane of each blot set to 1.00 (*n*=4 for all stages).

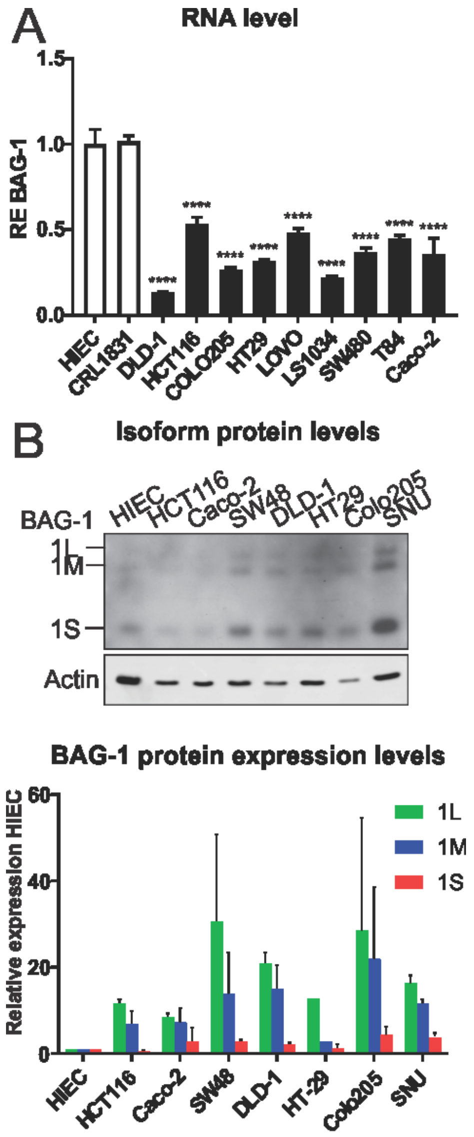

Supplementary Figure S2. BAG-1 mRNA and protein isoforms expression levels in normal intestinal epithelial and CRC cell lines.

(**A**) Relative RNA levels (RE) of BAG-1 as measured by RT-qPCR. The normal colorectal epithelial cell lines are in white, while the CRC cell lines ares in black. The bars represent the means with their standard deviations (n=3). The statistical test performed is a one-way ANOVA with Dunnett's Multiple comparison test. Statistical difference of the BAG-1 RNA RE levels of all cell lines were compared to the RE level of HIEC cells normalised at 1 ****$P \leq 0.0001$. (**B**) *Top:* Representative immunoblot of the BAG-1 protein isoforms in the normal HIEC cell line and in seven CRC cell lines. β-actin was used as the loading control. *Bottom:* Relative expression levels of the three protein isoforms compare to the HIEC normal cell line. The relative expression was measured as the isoforms band densities normalised with the actin loading control, all relative to the HIEC band density which was set to 1. The bars are the means and standard deviation (n=3).

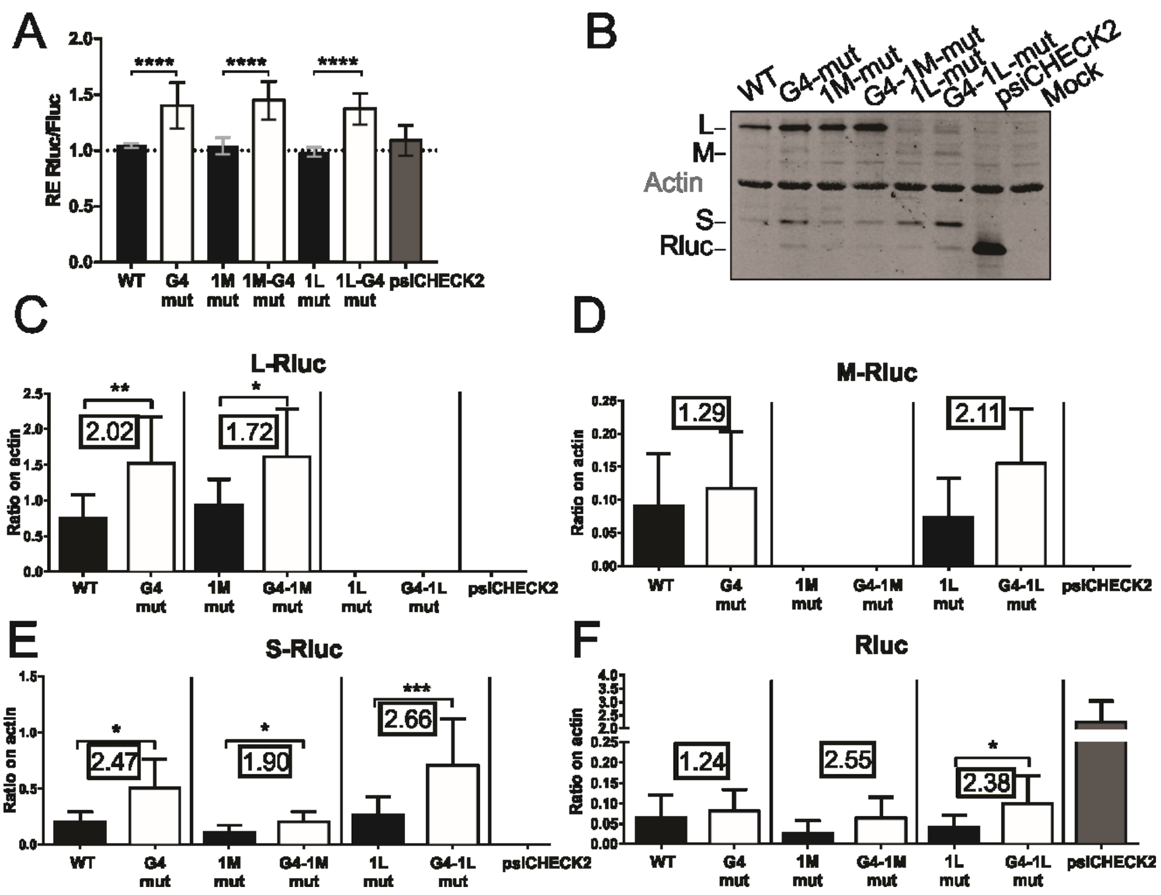

Supplementary Figure S3. RNA and protein isoforms expression levels of the reporter assays of the complete 5'UTR of BAG-1 with both the mutated rG4 and the mutated 1L or 1M start codons.

(**A**) The relative expression levels of the Rluc RNA, normalised over the Fluc RNA after the transfections of the different mutated constructions, as measured by RT-qPCR. The bar labeled psiCHECK-2 represents the reporter plasmid without the BAG-1 5'UTR insertion. The statistical test performed is a two-way ANOVA with Tukey's multiple comparison test, (n=3), ****P≤0.0001. (**B**) Representative immunoblot of the Rluc N-extension protein isoforms expression levels of the rG4 and either the 1L or the 1M start codon mutations. The psiCHECK-2 transfection lane represents the canonical Rluc without the N-terminal extension. Mock represents the untransfected control. β-actin was used as the loading control. (**C-F**) Quantification of the protein level of each isoform, normalised over the β-actin loading control. (**C**) L-Rluc, (**D**) M-Rluc, (**E**) S-Rluc, (**F**) Rluc. The boxed value is the fold-change in the protein level of the rG4mut construction over that of the WT. The statistical test performed is a Mann-Whitney test, (n=3), *P≤0.05, **P≤0.001, *** P≤0.0005.

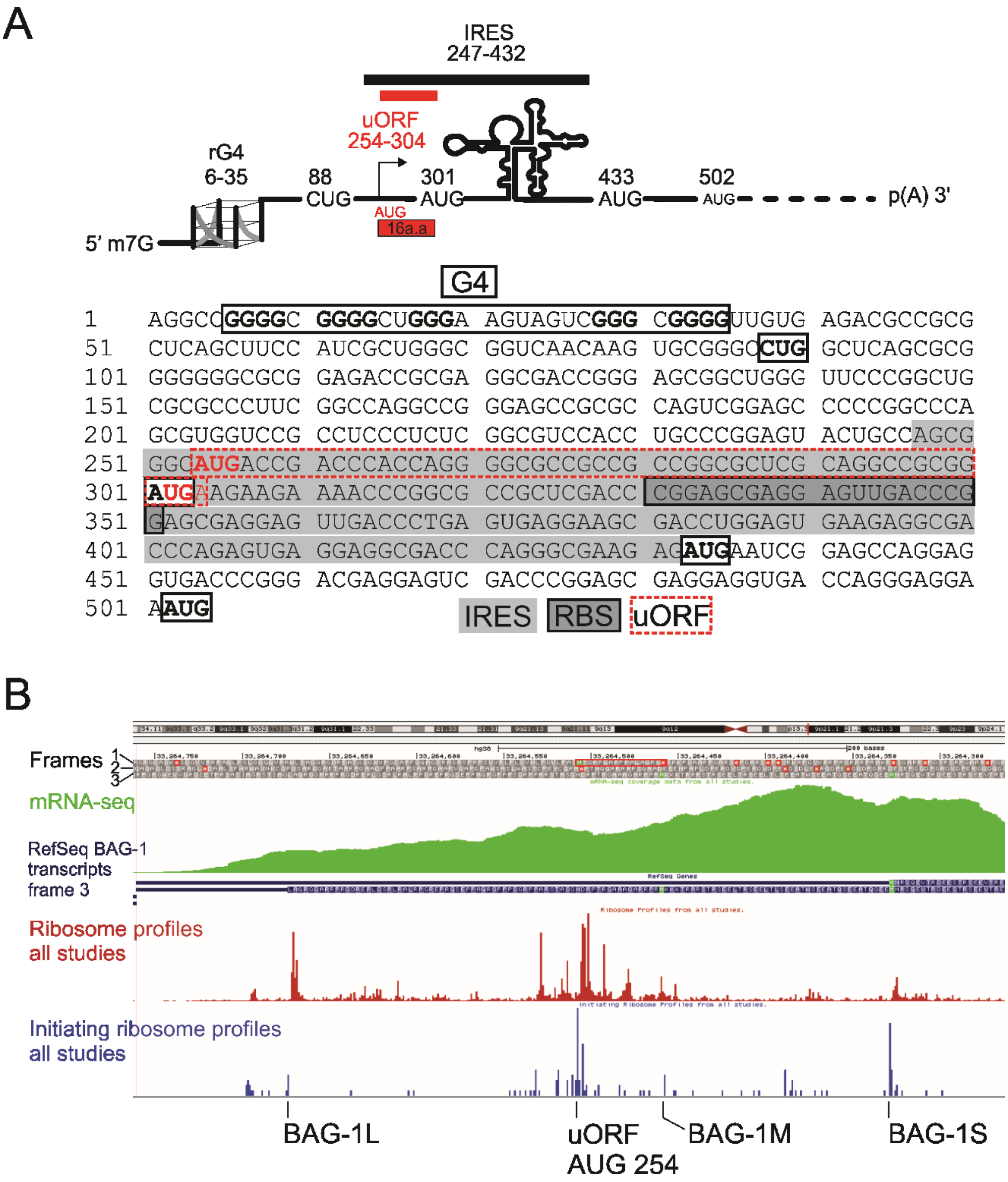

Supplementary Figure S4. The BAG-1 5'UTR possesses a repressive uORF located at position 254.

(A) Scheme of the BAG-1 5'UTR organisation showing the position of the possible uORF that is located within the 5'UTR. The uORF AUG-254 start codon and its UGA stop codon are highlighted in red. The uORF sequence is boxed with a dotted line. (B) Genome-browser view of the aggregate of the multiple ribosome-profiling studies demonstrating the initiation of translation at the AUG position 254.

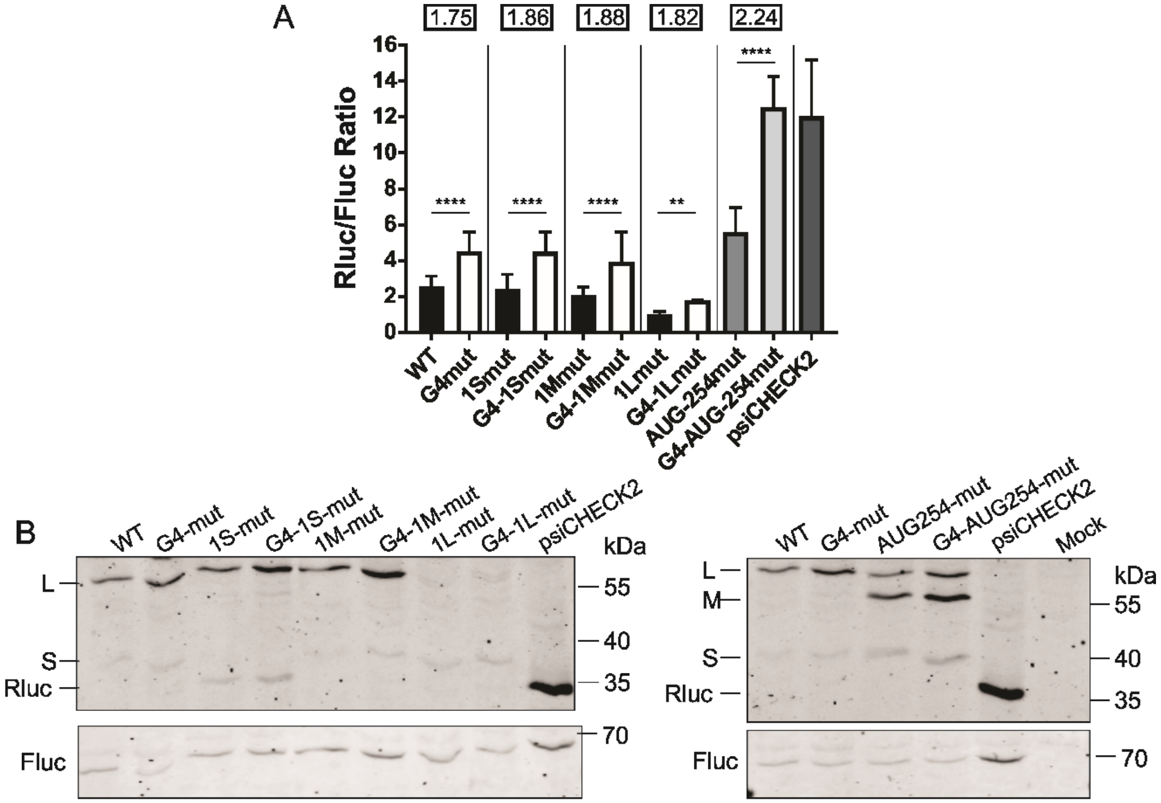

C

**Reporter constructs pairs Expression fold**	**RNA**	**Luc**	**Protein N-terminal isoforms**			
			**L-Rluc**	**M-Rluc**	**S-Rluc**	**Rluc**
G4mut/WT	1.36	1.76	5.28-3.00[1]	-	5.52-1.34[1]	-
1S-G4mut/1Smut	1.41	1.75	1.65	-	-	1.99
1M-G4mut/1Mmut	1.39	1.88	1.28	-	0.92	-
1L-G4mut/1Lmut	1.39	1.82	-	-	1.68	-
G4-AUG254mut/AUG254mut	1.47	2.35	1.83	1.89	1.63	-

1. WT and G4mut protein lysate are the same on both WB of panel B, the second ratio is from the WB on the left

Supplementary Figure S5. Comparison of Rluc/Fluc expression ratios of the different BAG-1 5'UTR reporter constructs at the luciferase, RNA and protein levels upon transfection of HCT116 cells.

(A) Rluc luciferase expression level normalised over the Fluc control expression level for all psiCHECK-2 constructions. Each construction bears the transfection control Fluc CDS, under the HSV-TK promoter and the complete BAG-1 5'UTR bearing mutations in the G4 region or at the start codons inserted upstream of the Rluc CDS, under the SV40 promoter, except for the construct labeled psiCHECK-2 which is the control without the BAG-1 5'UTR. Each bar is the mean and standard deviation of two biological replicates in which each constructions was transfected in

duplicate (n=2). Statistical significance is measured using Two-way ANOVA with Sidak's multiple comparisons, ****$P \leq 0.0001$, **$P \leq 0.01$. The ratios of expression of the G4mut construct over its related G4-WT construct are in boxes over each pair. (B) Representative Western blot of the different N-terminal Rluc isoforms and Fluc normalisation protein 24h post-transfection. Except for the psiCHECK-2 (no 5'UTR) control, the Fluc protein levels are equal between each construct pairs (anti-Luciferase mAb, clone21, #05-603 from EMD Millipore, 1:1000 in PBS-milk 4%). (C) Table presenting the ratios of the G4mut constructs expression levels over their respective G4-WT construct from the luciferase assays, the RT-qPCR assays and the Western blots. The ratios are the means from the results obtained in the luciferase assays and RT-qPCR assays presented here and from the main manuscript (Figure 4A-B, 5A-B, and Suppl. Fig 3A). The protein ratios are from the WB presented here only. Rluc expression levels were normalised over FLuc expression levels for the 3 types of assays. Increased expression from the G4mut constructions observed in the luciferase assays and WB does not arise only from transcriptional differences because both normalised luciferase expression and protein isoforms ratios are 1.3-fold higher than the RNA level ratio which suggest that the rG4 also affects post-transcriptional regulation, possibly translation.

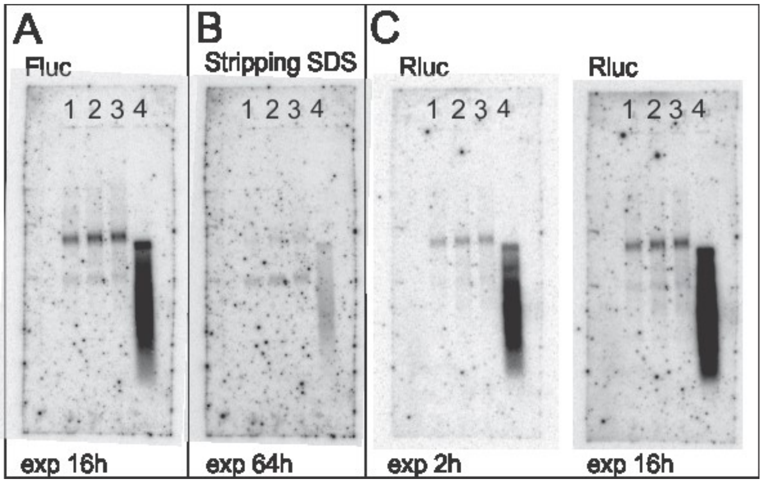

Supplementary Figure S6. Control of the bicistronic constructions integrity.

Total RNA was extracted from HCT116 cells transfected either with the pRL-HL (lane 1), the pRL-BAG1wt-HL (lane 2) or the pRL-BAG1g4mut-HL (lane 3) construction and was migrated on a denaturing agarose gel along with a positive control of *in vitro* transcribed bicistronic RNA derived from pRL-BAG-1wt-HL (lane 4). The gel was transferred to a Hybond XL membrane and probed using the specific Fluc or Rluc probes listed in **Supplementary Table S3** (**A**) Northern blot using the Fluc specific probe. (**B**) The same membrane as in (**A**) was exposed for 64 h following stripping in order to confirm the removal of the Fluc probe. (**C**) Two exposure times of the same Northern blot membrane using the Rluc specific probe. Except for the smear in lane 4 that was caused by the presence of incomplete *in vitro* transcribed sequences, only 1 band is present for each construction at the same position for both probes, indicating that the bicistronic constructions are intact, i.e.no cryptic promoters or splicing sites are present and possess both luciferases on the same RNA strand.

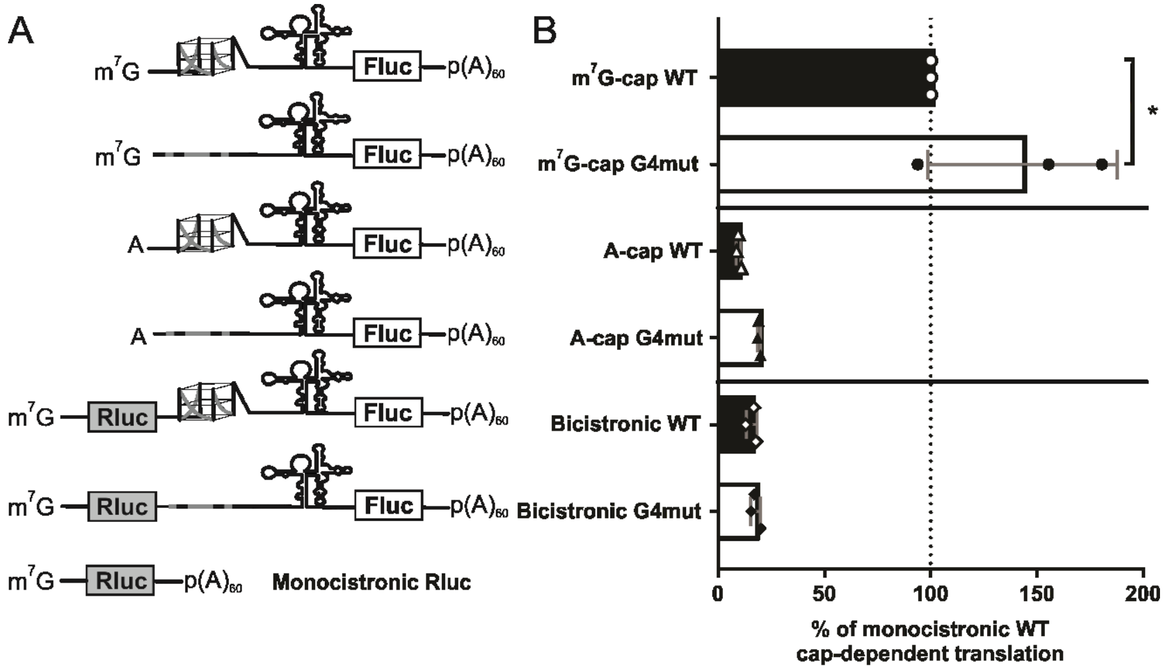

Supplementary Figure S7. Effects of the rG4 on both the cap-dependent and the cap-independent translation of the transfected mRNA reporter constructions.

(**A**) Schematic representation of the mRNA constructions used in the assay. They differ first by being either monocistronic (Fluc only) or bicistronic (Rluc cap-dependent and Fluc cap-independent) and second, by the presence of either the canonical m^7G-cap or the analog A-cap. The monocistronic Rluc mRNA serves as the co-transfection control for the Fluc/Rluc normalisation of the monocistronic constructions. (**B**) The translation level of each transfected mRNA construction is represented as a percentage relative to the monocistronic WT mRNA construction translation level set to 100%. The 100% level was set as the Fluc/Rluc ratio of the luciferase expression levels of the WT monocistronic construction, corrected by the RNA expression level as measured by RT-ddPCR. The assay was repeated three times with each construction transfected in triplicate. Each data point is the mean of the triplicate luciferase expression levels normalised over RNA expression levels. (n=3). The statistical analysis performed is a one-way ANOVA with Sidak's multiple comparisons test, *$P \leq 0.05$.

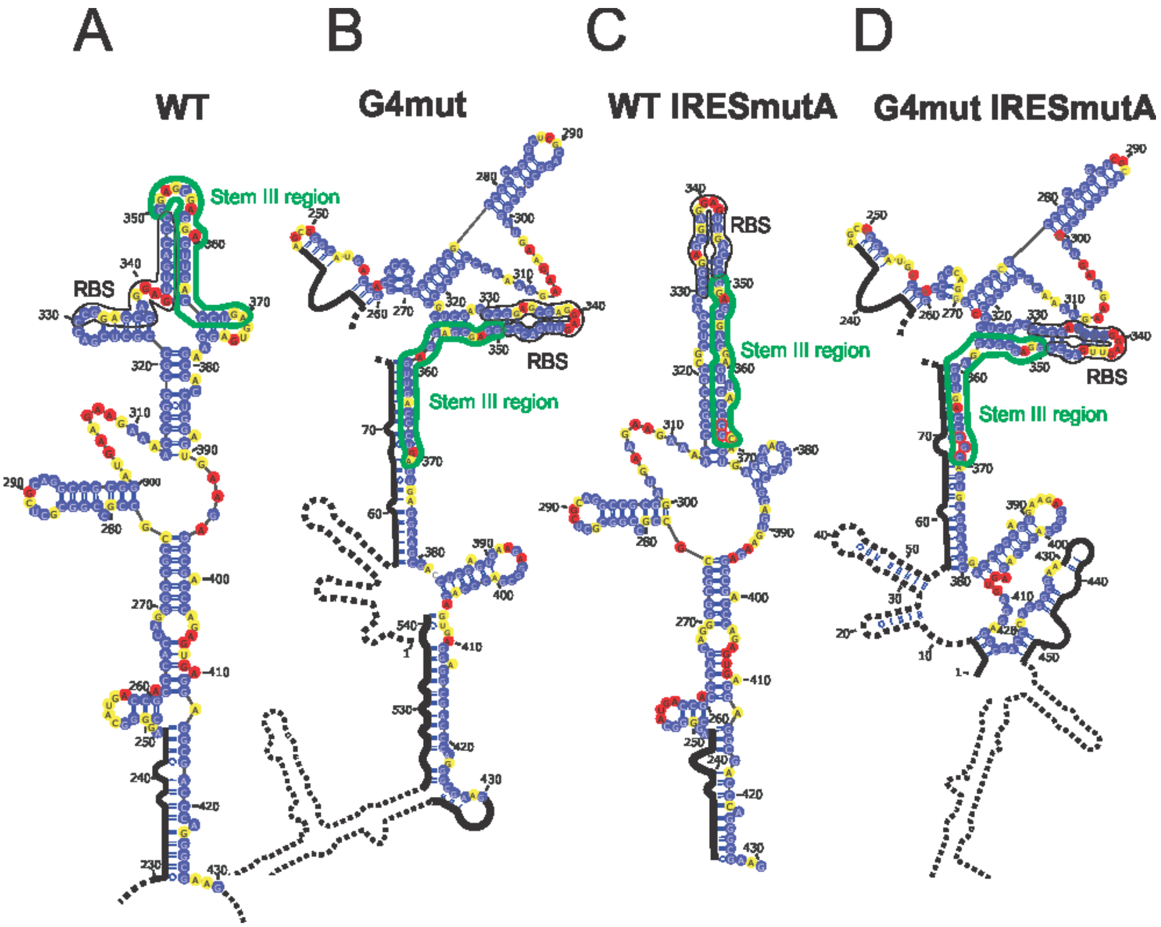

Supplementary Figure S8. Secondary structure elucidated by SHAPE of the minimal IRES region of the BAG-1 5'UTR.

(**A**) WT (**B**) rG4mut (**C**) WT and IRESmutA, and (**D**) rG4mut and IRESmutA. The colors represent the normalised SHAPE reactivity of each nucleotide. Blue, non-reactive; yellow, reactive; and, red, highly reactive. The RBS, the Stem III region and the IRES mut A mutation are all highlighted.

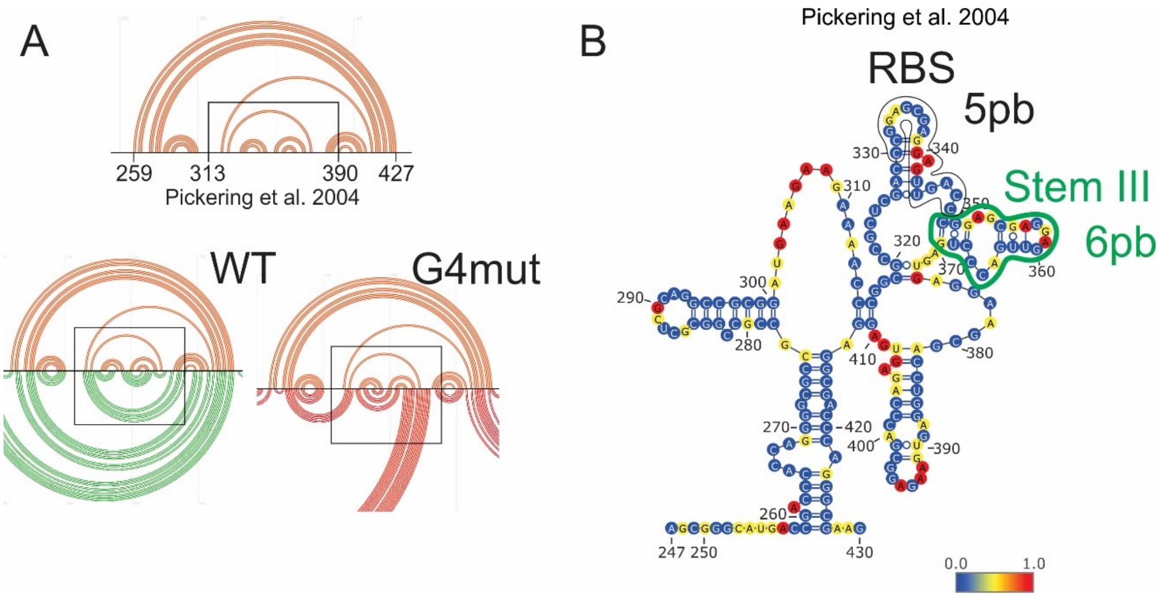

Supplementary Figure S9. Comparison of the IRES secondary structure elucidated by SHAPE using the WT complete BAG-1 5'UTR sequence with the structure elucidated by Pickering *et al.* 2004.

(**A**) Arc-plots of the secondary structures: shown are the IRES secondary structure previously proposed by Pickering *et al.* 2004 (orange) and the WT (green) and rG4mut (red) structures, both of which were proposed in this work. The nucleotide positions are indicated, the boxed region corresponds to the RBS and Stem-loop III domains. Of the total of 42 bp of the structure proposed by Pickering et al., 7 are identical with the WT structure and 12 with the rG4mut structure elucidated in this work. (**B**) Proposed secondary structure of the IRES region of Pickering *et al.* with the RBS and the Stem III highlighted. The color of the nucleotide represent the normalised SHAPE reactivity, as measured in this study, Blue, non-reactive; yellow, reactive; and, red, highly reactive. The reactivities obtained in this work do not agree entirely with the structure proposed by Pickering *et al*. 2004.

Supplementary Tables

Supplementary Table S1. Clinicopathological parameters of the CRC patients.

**Parameters**	**Value**
Age, median years (range)	69 (50-85)
Sex	
Male, no (%)	26 (55%)
Female, no (%)	24 (46%)
Tumor localization	
Left + rectum, no (%)	12 (26%)
Right + transverse, no (%)	34 (72%)
Polypes, no (%)	1 (2%)
Tumor stage (TNM)	
Adenoma, no (%)	9 (19%)
1, no (%)	8 (17%)
2, no (%)	10 (21%)
3, no (%)	10 (21%)
4, no (%)	10 (21%)

Supplementary Table S2. RNA and protein expression levels for each tissue pair presented as fold change of tumor expression level over margin expression level

		T/M-fold change			Protein T/M-fold corrected by the RNA T/M-fold		Protein expression compared to RNA expression level[1]
**Stage**	**Sample pair**	**RNA**	**Isoforms L-M**	**Isoform S**	**Isoforms L-M**	**Isoform S**	
Adenoma	A-1	0,67	4,02	1,35	5,97	2,01	+
	A-2	0,73	5,52	2,03	7,57	2,78	+
	A-3	-	8,73	1,95	-	-	
	A-4	0,51	2,01	1,08	3,95	2,13	+
	A-5	1,11	1,14	0,79	1,03	0,71	=
	A-6	0,93	1,92	1,19	2,06	1,28	+ / =
	A-7	0,36	1,18	1,01	3,24	2,80	+
	A-8	0,67	-	-	-	-	
	A-9	0,81	-	-	-	-	
Stage 1	1-1	0,45	0,94	1,20	2,07	2,64	+
	1-2	0,41	1,40	1,05	3,40	2,53	+
	1-3	0,21	0,08	0,26	0,39	1,25	- / =
	1-4	0,54	0,60	4,24	1,11	7,84	= / +
	1-5	1,04	0,70	0,30	0,68	0,29	= / -
	1-6	0,73	1,78	0,85	2,43	1,16	+ / =
	1-7	0,78	0,71	1,25	0,92	1,60	=
	1-8	1,02	-	-	-	-	
Stage 2	2-1	0,51	0,44	0,78	0,87	1,54	= / +
	2-2	0,91	2,38	0,54	2,62	0,60	+ / =
	2-3	0,69	0,85	1,17	1,22	1,69	= / +
	2-4	0,41	0,92	1,91	2,25	4,66	+
	2-5	0,75	0,79	2,12	1,06	2,82	= / +
	2-6	1,45	1,13	1,32	0,78	0,91	=
	2-7	0,74	0,73	0,89	0,98	1,20	=
	2-8	0,46	2,71	1,51	5,93	3,30	+
	2-9	0,47	-	-	-	-	
	2-10	0,15	-	-	-	-	
Stage 3	3-1	0,25	0,48	1,63	1,94	6,58	+
	3-2	0,45	0,29	0,36	0,63	0,80	=
	3-3	0,25	1,43	1,92	5,71	7,69	+
	3-4	0,71	0,64	1,03	0,91	1,45	=
	3-5	4,06	0,36	0,63	0,09	0,15	-
	3-6	0,85	0,38	0,36	0,44	0,42	-
	3-7	0,68	0,93	1,13	1,38	1,67	= / +
	3-8	0,69	0,59	0,38	0,85	0,55	=

	3-9	0,30	-	-	-	-	
	3-10	0,40	-	-	-	-	
Stage 4	4-1	0,33	0,60	0,97	1,82	2,97	**+**
	4-2	1,09	0,91	2,76	0,84	2,53	**= / +**
	4-3	0,60	4,69	6,09	7,80	10,12	**+**
	4-4	0,44	1,97	4,31	4,51	9,85	**+**
	4-5	0,72	1,88	1,07	2,62	1,50	**+**
	4-6	1,55	0,65	1,02	0,42	0,66	**- / =**
	4-7	0,26	1,44	1,04	5,57	4,01	**+**
	4-8	0,56	2,16	2,57	3,84	4,57	**+**
	4-9	0,80	-	-	-	-	
	4-10	0,36	-	-	-	-	
	4-11	0,84	-	-	-	-	

1. *Protein T/M-expression fold corrected by RNA T/M-expression fold are represented by a "+" if > 1.50, by "-" if < 0.50 and by "=" if between 0.50 and 1.50*

Supplementary Table S3. Translation initiation efficiency of the start codons of the BAG-1 5'UTR.

Isoforms	Frame	Translation initiation context	Translation initiation efficiency[1]
**BAG-1L**	3	GGGC**CUG**G	10.7
**BAG-1M**	3	CCGCGG**AUG**AA	60
**BAG-1S**	3	GAAGAG**AUG**AA	93
**AUG-254**	1	GCGGGC**AUG**AC	103
**AUG-254mut**	1	GCGGGC**ACG**AC	1
**Reference Initiation context[2]**	*NA*	GCCACC**AUG**GG	100

1. Refs:(1, 2)
2. Ref. (3)

Supplementary Table S4. List of primers and oligonucleotides used in this study**.**

Method	Name	Sequence 5'-3'
Cloning	5'UTR BAG-1 WT **NheI** restriction site	**GCTAGC**AGGCCGGGGCGGGGCTGGGAAGTAGTCGGGCGG<br>GGTTGTGAGACGCCGCGCTCAGCTTCCATCGCTGGGCGG<br>TCAACAAGTGCGGGCCTGGCTCAGCGCGGGGGGGCGCGG<br>AGACCGCGAGGCGACCGGGAGCGGCTGGGTTCCCGGCTG<br>CGCGCCCTTCGGCCAGGCCGGGAGCCGCGCCAGTCGGAG<br>CCCCCGGCCCAGCGTGGTCCGCCTCCCTCTCGGCGTCCA<br>CCTGCCCGGAGTACTGCCAGCGGGCATGACCGACCCACC<br>AGGGGCGCCGCCGCCGGCGCTCGCAGGCCGCGGATGAAG<br>AAGAAAACCCGGCGCCGCTCGACCCGGAGCGAGGAGTTG<br>ACCCGGAGCGAGGAGTTGACCCTGAGTGAGGAAGCGACC<br>TGGAGTGAAGAGGCGACCCAGAGTGAGGAGGCGACCCAG<br>GGCGAAGAGATGAATCGGAGCCAGGAGGTGACCCGGGAC<br>GAGGAGTCGACCCGGAGCGAGGAGGTGACCAGGGAGGAA<br>**GCTAGC**
	5'UTR BAG-1 G4mut **NheI** restriction site	**GCTAGC**AGGCCGAGACGAGACTGAGAAGTAGTCGAGCGA<br>GGTTGTGAGACGCCGCGCTCAGCTTCCATCGCTGGGCGG<br>TCAACAAGTGCGGGCCTGGCTCAGCGCGGGGGGGCGCGG<br>AGACCGCGAGGCGACCGGGAGCGGCTGGGTTCCCGGCTG<br>CGCGCCCTTCGGCCAGGCCGGGAGCCGCGCCAGTCGGAG<br>CCCCCGGCCCAGCGTGGTCCGCCTCCCTCTCGGCGTCCA<br>CCTGCCCGGAGTACTGCCAGCGGGCATGACCGACCCACC<br>AGGGGCGCCGCCGCCGGCGCTCGCAGGCCGCGGATGAAG<br>AAGAAAACCCGGCGCCGCTCGACCCGGAGCGAGGAGTTG<br>ACCCGGAGCGAGGAGTTGACCCTGAGTGAGGAAGCGACC<br>TGGAGTGAAGAGGCGACCCAGAGTGAGGAGGCGACCCAG<br>GGCGAAGAGATGAATCGGAGCCAGGAGGTGACCCGGGAC<br>GAGGAGTCGACCCGGAGCGAGGAGGTGACCAGGGAGGAA<br>**GCTAGC**
	5'UTR BAG-1 WT **1Smut NheI** restriction site	**GCTAGC**AGGCCGGGGCGGGGCTGGGAAGTAGTCGGGCGG<br>GGTTGTGAGACGCCGCGCTCAGCTTCCATCGCTGGGCGG<br>TCAACAAGTGCGGGCCTGGCTCAGCGCGGGGGGGCGCGG<br>AGACCGCGAGGCGACCGGGAGCGGCTGGGTTCCCGGCTG<br>CGCGCCCTTCGGCCAGGCCGGGAGCCGCGCCAGTCGGAG<br>CCCCCGGCCCAGCGTGGTCCGCCTCCCTCTCGGCGTCCA<br>CCTGCCCGGAGTACTGCCAGCGGGCATGACCGACCCACC<br>AGGGGCGCCGCCGCCGGCGCTCGCAGGCCGCGGATGAAG<br>AAGAAAACCCGGCGCCGCTCGACCCGGAGCGAGGAGTTG<br>ACCCGGAGCGAGGAGTTGACCCTGAGTGAGGAAGCGACC<br>TGGAGTGAAGAGGCGACCCAGAGTGAGGAGGCGACCCAG<br>GGCGAAGAG**AGG**AATCGGAGCCAGGAGGTGACCCGGGAC<br>GAGGAGTCGACCCGGAGCGAGGAGGTGACCAGGGAGGAA<br>**GCTAGC**
	5'UTR BAG-1 G4mut **1Smut NheI** restriction site	**GCTAGC**AGGCCGAGACGAGACTGAGAAGTAGTCGAGCGA<br>GGTTGTGAGACGCCGCGCTCAGCTTCCATCGCTGGGCGG<br>TCAACAAGTGCGGGCCTGGCTCAGCGCGGGGGGGCGCGG<br>AGACCGCGAGGCGACCGGGAGCGGCTGGGTTCCCGGCTG<br>CGCGCCCTTCGGCCAGGCCGGGAGCCGCGCCAGTCGGAG<br>CCCCCGGCCCAGCGTGGTCCGCCTCCCTCTCGGCGTCCA<br>CCTGCCCGGAGTACTGCCAGCGGGCATGACCGACCCACC<br>AGGGGCGCCGCCGCCGGCGCTCGCAGGCCGCGGATGAAG<br>AAGAAAACCCGGCGCCGCTCGACCCGGAGCGAGGAGTTG<br>ACCCGGAGCGAGGAGTTGACCCTGAGTGAGGAAGCGACC<br>TGGAGTGAAGAGGCGACCCAGAGTGAGGAGGCGACCCAG

		GGCGAAGAG**AGG**AATCGGAGCCAGGAGGTGACCCGGGAC GAGGAGTCGACCCGGAGCGAGGAGGTGACCAGGGAGGAA **GCTAGC**
Amplify 5'UTR	BAG1 complete 5UTR fwd	TCAGTCAGA**GCTAGC**AGGCCG
	BAG1 complete 5UTR rev	AGTCAGT**GCTAGC**TTCCTCCCTGGTCACCTCC
Mut start codon 1M	Q5-BAG-1Mmut_Fwd	AGGCCGCGGAGGAAGAAGAAAACCCGGCG
	Q5-BAG-1Mmut_Rev	GCGAGCGCCGGCGGCGGC
Mut start codon 1L	Fwd_psiCHECK_Nhe1	CGACTCACTATAGGCTAGCAGGCCG
	Rev_BAG1L _mut	CCGCGCTGAGCCCGGCCCGCACTTG
	Fwd_BAG1L_mut	CAAGTGCGGGCCGGGCTCAGCGCGG
	Rev_psiCHECK_Nhe1_end	GCCATGGTGGCTAGCGGTTCCTCCC
Bicistronic construct	Hpa1_Q5_F	GTTAACATGGAAGACGCCAAAAACATAAAGAAAGGC
	Hpa1_Q5_R	GACGTCCTGTGGGCGGCG
	Not1_BAG1_WT_5UTR_f	AAGCACGCGGCCGCAGGCCGGGGCGGGGCTGGG
	Not1_BAG1_G4mut_5UTR_f	AAGCACGCGGCCGCAGGCCGAGACGAGACTGAG
	HpaI_BAG1_5UTR_r	CCGTTAACTTCCTCCCTGGTCACCTCC
IRES mutations	Q5_stm3mutA_F	GGAGTTGACCGGCAGTGAGGAAGCGACC
	Q5_stm3mutA_R	TCGCTCCGGGTCAACTCC
	Q5_IRESmutB_F	CGACCCGGAGGTTGGAGTTGACCCGGAG
	Q5_IRESmutB_R	AGCGGCGCCGGGTTTTCT
qPCR	BAG1_G_f	GGAGAGTAAAAGCCACAATAGAGCAG
	BAG1_G_r	CTGTCTTTGAAATTTTCTGGCAGGAT
	MRPL19_G_3_f	AAGGAGAAAAGTACTCCACATTCCAGAG
	MRPL19_G_3_r	TGGGTCAGCTGTAGTAACACGA
	SDHA_G_f	TGTTGATGGGAACAAGAGGGCA
	SDHA_G_r	GCCTACCACCACTGCATCAAAT
	YWHAZ_G_f	TCCCCAATGCTTCACAAGCAGA
	YWHAZ_G_r	TCTTGTCATCACCAGCGGCAA
	fLuc.q.F2	GTGGGCAAGGTGGTGCCATT
	fLuc.q.R2	AATCATAGGGCCGCGCACAC
	rLuc.q.F2	AAGGGCCTCCACTTCAGCCA
	rLuc.q.R2	TTCTTCAGCACGCGCTCCAC
	fLuc_pRL-HL.q.F1	TGGCAGGTCTTCCCGACGAT
	fLuc_pRL-HL.q.R1	ACACAACTCCTCCGCGCAAC
	rLuc_pRL-HL.q.F1	ACATGGTAACGCGGCCTCTTC
	rLuc_pRL-HL.q.R1	ACCAGATTTGCCTGATTTGCCCA
ddPCR	PUM1_global_for_1	TGAGGTGTGCACCATGAAC
	PUM1_global_rev_1	CAGAATGTGCTTGCCATAGG
	B2M.qref.F3	ACTACACTGAATTCACCCCCACTGA
	B2M.qref.R3	GCTGCTTACATGTCTCGATCCCA
	MRPL19.qref.F1	TCATCGTGGACAAGCACCGC
	MRPL19.qref.R1	TCAGAGGATCTGTTCTTCCCCTTCG
	YWHAZ.qref.F2	TGAAGAGTCATACAAAGACAGCACGC
	YWHAZ.qref.R2	AGACAAAAGTTGGAAGGCCGGT
Northern Blot probes	Fluc_cds_pRL-HL	GGATCTCTCTGATTTTTCTTGCGTCGAG
	Rluc_cds_pRL-HL	CCATAAATAAGAAGAGGCCGCGTTACCA
Creation of DNA templates for mRNA synthesis	Q5_intercistron_WT_fwd	ATTGTAATACTCTAGAGGATCCCCCGGGCGAGCTCCCGC GGCCGCAGGCCGGGGCGG
	Q5_intercistron_G4mut_fwd	ATTGTAATACTCTAGAGGATCCCCCGGGCGAGCTCCCGC GGCCGCAGGCCGAGACGAG
	Q5_intercistron_rev	AGCTAAGAATTTCGTCATCGCTGAATACAGTTACATTTC TAGAATTATTGTTCATTTTTGAG

	P1-Rluc	CGCCGTAATACGACTCACTATAGGGCTAGCCACCATGACTTCGAAAG
	P1-s1-Rluc	CGCCGTAATACGACTCACTATAGGGAGTGGACTTCGGTCCACTCCCCTAGCCACCATGACTTCGAAAG
	P1-rev-Rluc	(T)$_{-60}$GGGAGCTCGCCCGGGGGATCC
	P2-BAG1	CGCCGTAATACGACTCACTATAGGGAGGCCGGGGCGGGGCTGGGAAGTAG
	P2-BAG1G4mut	CGCCGTAATACGACTCACTATAGGGAGGCCGAGACGAGACTGAGAAGTAG
	P2-S1-BAG1-short	CGCCGTAATACGACTCACTATAGGGAGTGGACTTCGGTCCACTCCCAGGCCGGGGCGGGGC
	P2-S1-BAG1G4mut	CGCCGTAATACGACTCACTATAGGGAGTGGACTTCGGTCCACTCCCAGGCCGAGACGAGACTGAGAAGTAG
	P3	(T)$_{-60}$GAATAGAATGACACCTACTCAGAC
	P3-long	(T)$_{-60}$GAATAGAATGACACCTACTCAGACAATGCGATGCAATTTC
	P4-Fluc	CGCCGTAATACGACTCACTATAGGGGAGGAAGTTAACATGGAAGA
SHAPE DNA template for *in vitro* trx	T3_BAG1wt_fwd	AATTAACCCTCACTAAAGAGGCCGGGGCGGGGCTGGGA
	T3_BAG1G4mut_fwd	AATTAACCCTCACTAAAGAGGCCGAGACGAGACTGAGA
	Rluc_psicheck-2_rev	GCTCGGGGTCGTACACCTTG
	SHAPE_BAG1IRESmutA-R	GCTCGGGGTCGTACACCTTGGAAGCCATGGTGGCTAGCGGTTCCTCCCTGGTCACCTCCT
SHAPE primers for RT	Shape_BAG1_rev	6-FAM GCTCGGGGTCGTACACCTTG
	Seq_BAG1_rev	NED GCTCGGGGTCGTACACCTTG
	Shape_BAG1no2_r	6-FAM CGCCGGGTTTTCTTCTTCAT
	Seq_BAG1no2_r	NED CGCCGGGTTTTCTTCTTCAT

Supplementary Table S5. Sequences of all of the 5’UTRs tested, those of the rG4 and the start codons that were mutated, the SHAPE WT and the mutated sequences.

Construction DNA	G4	Mutations	Sequence 5’-3’
BAG-1 complete 5'UTR	WT	WT	AGGCC<u>GGGGCGGGGCTGGGAAGTAGTCGGGCGGGG</u>TTGTGAGACGCCGCGCTCAGCTTCCATCGCTGGG<br>CGGTCAACAAGTGCGGGCCTGGCTCAGCGCGGGGGGGCGCGGAGACCGCGAGGCGACCGGGAGCGGCTG<br>GGTTCCCGGCTGCGCGCCCTTCGGCCAGGCCGGGAGCCGCGCCAGTCGGAGCCCCCGGCCCAGCGTGGT<br>CCGCCTCCCTCTCGGCGTCCACCTGCCCGGAGTACTGCCAGCGGGCATGACCGACCCACCAGGGGCGCC<br>GCCGCCGGCGCTCGCAGGCCGCGGATGAAGAAGAAAACCCGGCGCCGCTCGACCCGGAGCGAGGAGTTG<br>ACCCGGAGCGAGGAGTTGACCCTGAGTGAGGAAGCGACCTGGAGTGAAGAGGCGACCCAGAGTGAGGAG<br>GCGACCCAGGGCGAAGAGATGAATCGGAGCCAGGAGGTGACCCGGGACGAGGAGTCGACCCGGAGCGAG<br>GAGGTGACCAGGGAGGAA
		1S mut	AGGCC<u>GGGGCGGGGCTGGGAAGTAGTCGGGCGGGG</u>TTGTGAGACGCCGCGCTCAGCTTCCATCGCTGGG<br>CGGTCAACAAGTGCGGGCCTGGCTCAGCGCGGGGGGGCGCGGAGACCGCGAGGCGACCGGGAGCGGCTG<br>GGTTCCCGGCTGCGCGCCCTTCGGCCAGGCCGGGAGCCGCGCCAGTCGGAGCCCCCGGCCCAGCGTGGT<br>CCGCCTCCCTCTCGGCGTCCACCTGCCCGGAGTACTGCCAGCGGGCATGACCGACCCACCAGGGGCGCC<br>GCCGCCGGCGCTCGCAGGCCGCGGATGAAGAAGAAAACCCGGCGCCGCTCGACCCGGAGCGAGGAGTTG<br>ACCCGGAGCGAGGAGTTGACCCTGAGTGAGGAAGCGACCTGGAGTGAAGAGGCGACCCAGAGTGAGGAG<br>GCGACCCAGGGCGAAGAG<u>**AGG**</u>AATCGGAGCCAGGAGGTGACCCGGGACGAGGAGTCGACCCGGAGCGAG<br>GAGGTGACCAGGGAGGAA
		1M mut	AGGCC<u>GGGGCGGGGCTGGGAAGTAGTCGGGCGGGG</u>TTGTGAGACGCCGCGCTCAGCTTCCATCGCTGGG<br>CGGTCAACAAGTGCGGGCCTGGCTCAGCGCGGGGGGGCGCGGAGACCGCGAGGCGACCGGGAGCGGCTG<br>GGTTCCCGGCTGCGCGCCCTTCGGCCAGGCCGGGAGCCGCGCCAGTCGGAGCCCCCGGCCCAGCGTGGT<br>CCGCCTCCCTCTCGGCGTCCACCTGCCCGGAGTACTGCCAGCGGGCATGACCGACCCACCAGGGGCGCC<br>GCCGCCGGCGCTCGCAGGCCGCGG<u>**AGG**</u>AAGAAGAAAACCCGGCGCCGCTCGACCCGGAGCGAGGAGTTG<br>ACCCGGAGCGAGGAGTTGACCCTGAGTGAGGAAGCGACCTGGAGTGAAGAGGCGACCCAGAGTGAGGAG<br>GCGACCCAGGGCGAAGAGATGAATCGGAGCCAGGAGGTGACCCGGGACGAGGAGTCGACCCGGAGCGAG<br>GAGGTGACCAGGGAGGAA

		1L mut	AGGCCGGGGCGGGGCTGGGAAGTAGTCGGGCGGGGTTGTGAGACGCCGCGCTCAGCTTCCATCGCTGGG<br>CGGTCAACAAGTGCGGGC**CGG**GCTCAGCGCGGGGGGGCGCGGAGACCGCGAGGCGACCGGGAGCGGCTG<br>GGTTCCCGGCTGCGCGCCCTTCGGCCAGGCCGGGAGCCGCGCCAGTCGGAGCCCCCGGCCCAGCGTGGT<br>CCGCCTCCCTCTCGGCGTCCACCTGCCCGGAGTACTGCCAGCGGGCATGACCGACCCACCAGGGGCGCC<br>GCCGCCGGCGCTCGCAGGCCGCGGATGAAGAAGAAAACCCGGCGCCGCTCGACCCGGAGCGAGGAGTTG<br>ACCCGGAGCGAGGAGTTGACCCTGAGTGAGGAAGCGACCTGGAGTGAAGAGGCGACCCAGAGTGAGGAG<br>GCGACCCAGGGCGAAGAGATGAATCGGAGCCAGGAGGTGACCCGGGACGAGGAGTCGACCCGGAGCGAG<br>GAGGTGACCAGGGAGGAA
		Stem3mutA	AGGCCGGGGCGGGGCTGGGAAGTAGTCGGGCGGGGTTGTGAGACGCCGCGCTCAGCTTCCATCGCTGGG<br>CGGTCAACAAGTGCGGGCCTGGCTCAGCGCGGGGGGGCGCGGAGACCGCGAGGCGACCGGGAGCGGCTG<br>GGTTCCCGGCTGCGCGCCCTTCGGCCAGGCCGGGAGCCGCGCCAGTCGGAGCCCCCGGCCCAGCGTGGT<br>CCGCCTCCCTCTCGGCGTCCACCTGCCCGGAGTACTGCCAGCGGGCATGACCGACCCACCAGGGGCGCC<br>GCCGCCGGCGCTCGCAGGCCGCGGATGAAGAAGAAAACCCGGCGCCGCTCGACCCGGAGCGAGGAGTTG<br>ACCCGGAGCGAGGAGTTGACC**GGC**AGTGAGGAAGCGACCTGGAGTGAAGAGGCGACCCAGAGTGAGGAG<br>GCGACCCAGGGCGAAGAGATGAATCGGAGCCAGGAGGTGACCCGGGACGAGGAGTCGACCCGGAGCGAG<br>GAGGTGACCAGGGAGGAA
		Stem3mutB	AGGCCGGGGCGGGGCTGGGAAGTAGTCGGGCGGGGTTGTGAGACGCCGCGCTCAGCTTCCATCGCTGGG<br>CGGTCAACAAGTGCGGGCCTGGCTCAGCGCGGGGGGGCGCGGAGACCGCGAGGCGACCGGGAGCGGCTG<br>GGTTCCCGGCTGCGCGCCCTTCGGCCAGGCCGGGAGCCGCGCCAGTCGGAGCCCCCGGCCCAGCGTGGT<br>CCGCCTCCCTCTCGGCGTCCACCTGCCCGGAGTACTGCCAGCGGGCATGACCGACCCACCAGGGGCGCC<br>GCCGCCGGCGCTCGCAGGCCGCGGATGAAGAAGAAAACCCGGCGCCGCTCGACCCGGAGCGAGGAGTTG<br>ACCCGGAG**GTT**GGAGTTGACCCTGAGTGAGGAAGCGACCTGGAGTGAAGAGGCGACCCAGAGTGAGGAG<br>GCGACCCAGGGCGAAGAGATGAATCGGAGCCAGGAGGTGACCCGGGACGAGGAGTCGACCCGGAGCGAG<br>GAGGTGACCAGGGAGGAA
	G4mut	WT	AGGCCG**A**G**A**CG**A**G**A**CTG**A**GAAGTAGTCG**A**GCG**A**GGTTGTGAGACGCCGCGCTCAGCTTCCATCGCTGGG<br>CGGTCAACAAGTGCGGGCCTGGCTCAGCGCGGGGGGGCGCGGAGACCGCGAGGCGACCGGGAGCGGCTG<br>GGTTCCCGGCTGCGCGCCCTTCGGCCAGGCCGGGAGCCGCGCCAGTCGGAGCCCCCGGCCCAGCGTGGT<br>CCGCCTCCCTCTCGGCGTCCACCTGCCCGGAGTACTGCCAGCGGGCATGACCGACCCACCAGGGGCGCC<br>GCCGCCGGCGCTCGCAGGCCGCGGATGAAGAAGAAAACCCGGCGCCGCTCGACCCGGAGCGAGGAGTTG<br>ACCCGGAGCGAGGAGTTGACCCTGAGTGAGGAAGCGACCTGGAGTGAAGAGGCGACCCAGAGTGAGGAG<br>GCGACCCAGGGCGAAGAGATGAATCGGAGCCAGGAGGTGACCCGGGACGAGGAGTCGACCCGGAGCGAG<br>GAGGTGACCAGGGAGGAA

		1Smut	AGGCCG**A**G**A**CG**A**G**A**CTG**A**GAAGTAGTCG**A**GCG**A**GGTTGTGAGACGCCGCGCTCAGCTTCCATCGCTGGG<br>CGGTCAACAAGTGCGGGCCTGGCTCAGCGCGGGGGGGCGCGGAGACCGCGAGGCGACCGGGAGCGGCTG<br>GGTTCCCGGCTGCGCGCCCTTCGGCCAGGCCGGGAGCCGCGCCAGTCGGAGCCCCCGGCCCAGCGTGGT<br>CCGCCTCCCTCTCGGCGTCCACCTGCCCGGAGTACTGCCAGCGGGCATGACCGACCCACCAGGGGCGCC<br>GCCGCCGGCGCTCGCAGGCCGCGGATGAAGAAGAAAACCCGGCGCCGCTCGACCCGGAGCGAGGAGTTG<br>ACCCGGAGCGAGGAGTTGACCCTGAGTGAGGAAGCGACCTGGAGTGAAGAGGCGACCCAGAGTGAGGAG<br>GCGACCCAGGGCGAAGAG**AGG**AATCGGAGCCAGGAGGTGACCCGGGACGAGGAGTCGACCCGGAGCGAG<br>GAGGTGACCAGGGAGGAA
		1Mmut	AGGCCG**A**G**A**CG**A**G**A**CTG**A**GAAGTAGTCG**A**GCG**A**GGTTGTGAGACGCCGCGCTCAGCTTCCATCGCTGGG<br>CGGTCAACAAGTGCGGGCCTGGCTCAGCGCGGGGGGGCGCGGAGACCGCGAGGCGACCGGGAGCGGCTG<br>GGTTCCCGGCTGCGCGCCCTTCGGCCAGGCCGGGAGCCGCGCCAGTCGGAGCCCCCGGCCCAGCGTGGT<br>CCGCCTCCCTCTCGGCGTCCACCTGCCCGGAGTACTGCCAGCGGGCATGACCGACCCACCAGGGGCGCC<br>GCCGCCGGCGCTCGCAGGCCGCGG**AGG**AAGAAGAAAACCCGGCGCCGCTCGACCCGGAGCGAGGAGTTG<br>ACCCGGAGCGAGGAGTTGACCCTGAGTGAGGAAGCGACCTGGAGTGAAGAGGCGACCCAGAGTGAGGAG<br>GCGACCCAGGGCGAAGAGATGAATCGGAGCCAGGAGGTGACCCGGGACGAGGAGTCGACCCGGAGCGAG<br>GAGGTGACCAGGGAGGAA
		1Lmut	AGGCCG**A**G**A**CG**A**G**A**CTG**A**GAAGTAGTCG**A**GCG**A**GGTTGTGAGACGCCGCGCTCAGCTTCCATCGCTGGG<br>CGGTCAACAAGTGCGGGCCTGGCTCAGCGCGGGGGGGCGCGGAGACCGCGAGGCGACCGGGAGCGGCTG<br>GGTTCCCGGCTGCGCGCCCTTCGGCCAGGCCGGGAGCCGCGCCAGTCGGAGCCCCCGGCCCAGCGTGGT<br>CCGCCTCCCTCTCGGCGTCCACCTGCCCGGAGTACTGCCAGCGGGCATGACCGACCCACCAGGGGCGCC<br>GCCGCCGGCGCTCGCAGGCCGCGGATGAAGAAGAAAACCCGGCGCCGCTCGACCCGGAGCGAGGAGTTG<br>ACCCGGAGCGAGGAGTTGACCCTGAGTGAGGAAGCGACCTGGAGTGAAGAGGCGACCCAGAGTGAGGAG<br>GCGACCCAGGGCGAAGAG**AGG**AATCGGAGCCAGGAGGTGACCCGGGACGAGGAGTCGACCCGGAGCGAG<br>GAGGTGACCAGGGAGGAA
		Stem3mutA	AGGCCG**A**G**A**CG**A**G**A**CTG**A**GAAGTAGTCG**A**GCG**A**GGTTGTGAGACGCCGCGCTCAGCTTCCATCGCTGGG<br>CGGTCAACAAGTGCGGGCCTGGCTCAGCGCGGGGGGGCGCGGAGACCGCGAGGCGACCGGGAGCGGCTG<br>GGTTCCCGGCTGCGCGCCCTTCGGCCAGGCCGGGAGCCGCGCCAGTCGGAGCCCCCGGCCCAGCGTGGT<br>CCGCCTCCCTCTCGGCGTCCACCTGCCCGGAGTACTGCCAGCGGGCATGACCGACCCACCAGGGGCGCC<br>GCCGCCGGCGCTCGCAGGCCGCGGATGAAGAAGAAAACCCGGCGCCGCTCGACCCGGAGCGAGGAGTTG<br>ACCCGGAGCGAGGAGTTGACC**GGC**AGTGAGGAAGCGACCTGGAGTGAAGAGGCGACCCAGAGTGAGGAG<br>GCGACCCAGGGCGAAGAGATGAATCGGAGCCAGGAGGTGACCCGGGACGAGGAGTCGACCCGGAGCGAG<br>GAGGTGACCAGGGAGGAA

		Stem3mutB	AGGCCG**A**G**A**CG**A**G**A**CTG**A**GAAGTAGTCG**A**GCG**A**GGTTGTGAGACGCCGCGCTCAGCTTCCATCGCTGGG<br>CGGTCAACAAGTGCGGGC**CGG**GCTCAGCGGGGGGGCGCGGAGACCGCGAGGCGACCGGGAGCGGCTG<br>GGTTCCCGGCTGCGCGCCCTTCGGCCAGGCCGGGAGCCGCGCCAGTCGGAGCCCCCGGCCCAGCGTGGT<br>CCGCCTCCCTCTCGGCGTCCACCTGCCCGGAGTACTGCCAGCGGGCATGACCGACCCACCAGGGGCGCC<br>GCCGCCGGCGCTCGCAGGCCGCGGATGAAGAAGAAAACCCGGCGCCGCTCGACCCGGAGCGAGGAGTTG<br>ACCCGGAG**GTT**GGAGTTGACCCTGAGTGAGGAAGCGACCTGGAGTGAAGAGGCGACCCAGAGTGAGGAG<br>GCGACCCAGGGCGAAGAGATGAATCGGAGCCAGGAGGTGACCCGGGACGAGGAGTCGACCCGGAGCGAG<br>GAGGTGACCAGGGAGGAA
**DNA template for *in vitro* transcription of RNA for SHAPE**	**G4**	**mutation IRES**	**Sequence 5’-3’**
BAG-1 5'UTR SHAPE T3 promoter, not transcribed excepted the last **G**<br>Extra sequence for RT primer binding	WT	WT	AATTAACCCTCACTAAA**G**AGGCCGGGGCGGGGCTGGAAGTAGTCGGGCGGGGTTGTGAGACGCCGCGC<br>TCAGCTTCCATCGCTGGGCGGTCAACAAGTGCGGGCCTGGCTCAGCGCGGGGGGGCGCGGAGACCGCGA<br>GGCGACCGGGAGCGGCTGGGTTCCCGGCTGCGCGCCCTTCGGCCAGGCCGGGAGCCGCGCCAGTCGGAG<br>CCCCCGGCCCAGCGTGGTCCGCCTCCCTCTCGGCGTCCACCTGCCCGGAGTACTGCCAGCGGGCATGAC<br>CGACCCACCAGGGGCGCCGCCGCCGGCGCTCGCAGGCCGCGGATGAAGAAGAAAACCCGGCGCCGCTCG<br>ACCCGGAGCGAGGAGTTGACCCGGAGCGAGGAGTTGACCCTGAGTGAGGAAGCGACCTGGAGTGAAGAG<br>GCGACCCAGAGTGAGGAGGCGACCCAGGGCGAAGAGATGAATCGGAGCCAGGAGGTGACCCGGGACGAG<br>GAGTCGACCCGGAGCGAGGAGGTGACCAGGGAGGAACCGCTAGCCACCATGGCTTCCAAGGTGTACGAC<br>CCCGAGC
		Stem3mutA	AATTAACCCTCACTAAA**G**AGGCCGGGGCGGGGCTGGAAGTAGTCGGGCGGGGTTGTGAGACGCCGCGC<br>TCAGCTTCCATCGCTGGGCGGTCAACAAGTGCGGGCCTGGCTCAGCGCGGGGGGGCGCGGAGACCGCGA<br>GGCGACCGGGAGCGGCTGGGTTCCCGGCTGCGCGCCCTTCGGCCAGGCCGGGAGCCGCGCCAGTCGGAG<br>CCCCCGGCCCAGCGTGGTCCGCCTCCCTCTCGGCGTCCACCTGCCCGGAGTACTGCCAGCGGGCATGAC<br>CGACCCACCAGGGGCGCCGCCGCCGGCGCTCGCAGGCCGCGGATGAAGAAGAAAACCCGGCGCCGCTCG<br>ACCCGGAGCGAGGAGTTGACCCGGAGCGAGGAGTTGACC**GGC**AGTGAGGAAGCGACCTGGAGTGAAGAG<br>GCGACCCAGAGTGAGGAGGCGACCCAGGGCGAAGAGATGAATCGGAGCCAGGAGGTGACCCGGGACGAG<br>GAGTCGACCCGGAGCGAGGAGGTGACCAGGGAGGAACCGCTAGCCACCATGGCTTCCAAGGTGTACGAC<br>CCCGAGC

	G4mut	WT	AATTAACCCTCACTAAA**G**AGGCCG**A**G**A**CG**A**G**A**CTG**A**GAAGTAGTCG**A**GCG**A**GGTTGTGAGACGCCGCGC<br>TCAGCTTCCATCGCTGGGCGGTCAACAAGTGCGGGCCTGGCTCAGCGCGGGGGGGCGCGGAGACCGCGA<br>GGCGACCGGGAGCGGCTGGGTTCCCGGCTGCGCGCCCTTCGGCCAGGCCGGGAGCCGCGCCAGTCGGAG<br>CCCCCGGCCCAGCGTGGTCCGCCTCCCTCTCGGCGTCCACCTGCCCGGAGTACTGCCAGCGGGCATGAC<br>CGACCCACCAGGGGCGCCGCCGCCGGCGCTCGCAGGCCGCGGATGAAGAAGAAAACCCGGCGCCGCTCG<br>ACCCGGAGCGAGGAGTTGACCCGGAGCGAGGAGTTGACCCTGAGTGAGGAAGCGACCTGGAGTGAAGAG<br>GCGACCCAGAGTGAGGAGGCGACCCAGGGCGAAGAGATGAATCGGAGCCAGGAGGTGACCCGGGACGAG<br>GAGTCGACCCGGAGCGAGGAGGTGACCAGGGAGGAACCGCTAGCCACCATGGCTTCCAAGGTGTACGAC<br>CCCGAGC
		Stem3mutA	AATTAACCCTCACTAAA**G**AGGCCG**A**G**A**CG**A**G**A**CTG**A**GAAGTAGTCG**A**GCG**A**GGTTGTGAGACGCCGCGC<br>TCAGCTTCCATCGCTGGGCGGTCAACAAGTGCGGGCCTGGCTCAGCGCGGGGGGGCGCGGAGACCGCGA<br>GGCGACCGGGAGCGGCTGGGTTCCCGGCTGCGCGCCCTTCGGCCAGGCCGGGAGCCGCGCCAGTCGGAG<br>CCCCCGGCCCAGCGTGGTCCGCCTCCCTCTCGGCGTCCACCTGCCCGGAGTACTGCCAGCGGGCATGAC<br>CGACCCACCAGGGGCGCCGCCGCCGGCGCTCGCAGGCCGCGGATGAAGAAGAAAACCCGGCGCCGCTCG<br>ACCCGGAGCGAGGAGTTGACCCGGAGCGAGGAGTTGACC**GGC**AGTGAGGAAGCGACCTGGAGTGAAGAG<br>GCGACCCAGAGTGAGGAGGCGACCCAGGGCGAAGAGATGAATCGGAGCCAGGAGGTGACCCGGGACGAG<br>GAGTCGACCCGGAGCGAGGAGGTGACCAGGGAGGAACCGCTAGCCACCATGGCTTCCAAGGTGTACGAC<br>CCCGAGC

Supplementary Table S6. Sequences of the transfected mono- and bicistronic mRNAs.

DNA templates for mRNA transfection	Sequence 5'-3'
**Ctrl Monocistronic Rluc 1111bp**<br>T7 promoter, not transcribed except last 3 Gs<br>Rluc cds	CGCCGTAATACGACTCACTATA**G**GGCTAGCCACCATGACTTCGAAAGTTTATGATCCAGAACAAAGGAA<br>ACGGATGATAACTGGTCCGCAGTGGTGGGCCAGATGTAAACAAATGAATGTTCTTGATTCATTTATTAA<br>TTATTATGATTCAGAAAAACATGCAGAAAATGCTGTTATTTTTTTACATGGTAACGCGGCCTCTTCTTA<br>TTTATGGCGACATGTTGTGCCACATATTGAGCCAGTAGCGCGGTGTATTATACCAGACCTTATTGGTAT<br>GGGCAAATCAGGCAAATCTGGTAATGGTTCTTATAGGTTACTTGATCATTACAAATATCTTACTGCATG<br>GTTTGAACTTCTTAATTTACCAAAGAAGATCATTTTTGTCGGCCATGATTGGGGTGCTTGTTTGGCATT<br>TCATTATAGCTATGAGCATCAAGATAAGATCAAAGCAATAGTTCACGCTGAAAGTGTAGTAGATGTGAT<br>TGAATCATGGGATGAATGGCCTGATATTGAAGAAGATATTGCGTTGATCAAATCTGAAGAAGGAGAAAA<br>AATGGTTTTGGAGAATAACTTCTTCGTGGAAACCATGTTGCCATCAAAAATCATGAGAAAGTTAGAACC<br>AGAAGAATTTGCAGCATATCTTGAACCATTCAAAGAGAAAGGTGAAGTTCGTCGTCCAACATTATCATG

	GCCTCGTGAAATCCCGTTAGTAAAAGGTGGTAAACCTGACGTTGTACAAATTGTTAGGAATTATAATGC<br>TTATCTACGTGCAAGTGATGATTTACCAAAAATGTTTATTGAATCGGACCCAGGATTCTTTTCCAATGC<br>TATTGTTGAAGGTGCCAAGAAGTTTCCTAATACTGAATTTGTCAAAGTAAAAGGTCTTCATTTTTCGCA<br>AGAAGATGCACCTGATGAAATGGGAAAATATATCAAATCGTTCGTTGAGCGAGTTCTCAAAAATGAACA<br>ATAATTCTAGAAATGTAACTGTATTCAGCGATGACGAAATTCTTAGCTATTGTAATACTCTAGAGGATC<br>CCCCGGGCGAGCTCCCTTTTTTTTTTTTTTTTTTTTTTTTTTTTTTTTTTTTTTTTTTTTTTTTTTTTT<br>TTTTTTT
**Ctrl Monocistronic Fluc 1950bp**<br>Fluc cds	CGCCGTAATACGACTCACTATA**G**GGGAGGAAGTTAACATGGAAGACGCCAAAAACATAAAGAAAGGCCC<br>GGCGCCATTCTATCCTCTAGAGGATGGAACCGCTGGAGAGCAACTGCATAAGGCTATGAAGAGATACGC<br>CCTGGTTCCTGGAACAATTGCTTTTACAGATGCACATATCGAGGTGAACATCACGTACGCGGAATACTT<br>CGAAATGTCCGTTCGGTTGGCAGAAGCTATGAAACGATATGGGCTGAATACAAATCACAGAATCGTCGT<br>ATGCAGTGAAAACTCTCTTCAATTCTTTATGCCGGTGTTGGGCGCGTTATTTATCGGAGTTGCAGTTGC<br>GCCCGCGAACGACATTTATAATGAACGTGAATTGCTCAACAGTATGAACATTTCGCAGCCTACCGTAGT<br>GTTTGTTTCCAAAAAGGGGTTGCAAAAAATTTTGAACGTGCAAAAAAAATTACCAATAATCCAGAAAAT<br>TATTATCATGGATTCTAAAACGGATTACCAGGGATTTCAGTCGATGTACACGTTCGTCACATCTCATCT<br>ACCTCCCGGTTTTAATGAATACGATTTTGTACCAGAGTCCTTTGATCGTGACAAAACAATTGCACTGAT<br>AATGAATTCCTCTGGATCTACTGGGTTACCTAAGGGTGTGGCCCTTCCGCATAGAACTGCCTGCGTCAG<br>ATTCTCGCATGCCAGAGATCCTATTTTTGGCAATCAAATCATTCCGGATACTGCGATTTTAAGTGTTGT<br>TCCATTCCATCACGGTTTTGGAATGTTTACTACACTCGGATATTTGATATGTGGATTTCGAGTCGTCTT<br>AATGTATAGATTTGAAGAAGAGCTGTTTTTACGATCCCTTCAGGATTACAAAATTCAAAGTGCGTTGCT<br>AGTACCAACCCTATTTTCATTCTTCGCCAAAAGCACTCTGATTGACAAATACGATTTATCTAATTTACA<br>CGAAATTGCTTCTGGGGGCGCACCTCTTTCGAAAGAAGTCGGGGAAGCGGTTGCAAAACGCTTCCATCT<br>TCCAGGGATACGACAAGGATATGGGCTCACTGAGACTACATCAGCTATTCTGATTACACCCGAGGGGGA<br>TGATAAACCGGGCGCGGTCGGTAAAGTTGTTCCATTTTTTGAAGCGAAGGTTGTGGATCTGGATACCGG<br>GAAAACGCTGGGCGTTAATCAGAGAGGCGAATTATGTGTCAGAGGACCTATGATTATGTCCGGTTATGT<br>AAACAATCCGGAAGCGACCAACGCCTTGATTGACAAGGATGGATGGCTACATTCTGGAGACATAGCTTA<br>CTGGGACGAAGACGAACACTTCTTCATAGTTGACCGCTTGAAGTCTTTAATTAAATACAAAGGATATCA<br>GGTGGCCCCCGCTGAATTGGAATCGATATTGTTACAACACCCCAACATCTTCGACGCGGGCGTGGCAGG<br>TCTTCCCGACGATGACGCCGGTGAACTTCCCGCCGCCGTTGTTGTTTTGGAGCACGGAAAGACGATGAC<br>GGAAAAAGAGATCGTGGATTACGTGGCCAGTCAAGTAACAACCGCGAAAAAGTTGCGCGGAGGAGTTGT<br>GTTTGTGGACGAAGTACCGAAAGGTCTTACCGGAAAACTCGACGCAAGAAAAATCAGAGAGATCCTCAT<br>AAAGGCCAAGAAGGGCGGAAAGTCCAAATTGTAAGGATCCGGGCCCTATTCTATAGTGTCACCTAAATG<br>CTAGAGCTCGCTGATCAGCCTCGACTGTGCCTTCTAGTTGCCAGCCATCTGTTGTTTGCCCCTCCCCCG<br>TGCCTTCCTTGACCCTGGAAGGTGCCACTCCCACTGTCCTTTCCTAATAAAATGAGGAAATTGCATCGC

	ATTGTCTGAGTAGGTGTCATTCTATTCTTTTTTTTTTTTTTTTTTTTTTTTTTTTTTTTTTTTTTTTTT<br>TTTTTTTTTTTTTTTTTT
**Monocistronic Fluc WT 2445bp**	CGCCGTAATACGACTCACTATA**G**GGAGGCC<u>**GGGG**C**GGGG**CT**GGG**AAGTAGTC**GGG**C**GGGG**</u>TTGTGAGAC<br>GCCGCGCTCAGCTTCCATCGCTGGGCGGTCAACAAGTGCGGGCCTGGCTCAGCGCGGGGGGGCGCGGAG<br>ACCGCGAGGCGACCGGGAGCGGCTGGGTTCCCGGCTGCGCGCCCTTCGGCCAGGCCGGGAGCCGCGCCA<br>GTCGGAGCCCCCGGCCCAGCGTGGTCCGCCTCCCTCTCGGCGTCCACCTGCCCGGAGTACTGCCAGCGG<br>GCATGACCGACCCACCAGGGGCGCCGCCGCCGGCGCTCGCAGGCCGCGGATGAAGAAGAAAACCCGGCG<br>CCGCTCGACCCGGAGCGAGGAGTTGACCCGGAGCGAGGAGTTGACCCTGAGTGAGGAAGCGACCTGGAG<br>TGAAGAGGCGACCCAGAGTGAGGAGGCGACCCAGGGCGAAGAGATGAATCGGAGCCAGGAGGTGACCCG<br>GGACGAGGAGTCGACCCGGAGCGAGGAGGTGACCAGGGAGGAAGTTAACATGGAAGACGCCAAAAACAT<br>AAAGAAAGGCCCGGCGCCATTCTATCCTCTAGAGGATGGAACCGCTGGAGAGCAACTGCATAAGGCTAT<br>GAAGAGATACGCCCTGGTTCCTGGAACAATTGCTTTTACAGATGCACATATCGAGGTGAACATCACGTA<br>CGCGGAATACTTCGAAATGTCCGTTCGGTTGGCAGAAGCTATGAAACGATATGGGCTGAATACAAATCA<br>CAGAATCGTCGTATGCAGTGAAAACTCTCTTCAATTCTTTATGCCGGTGTTGGGCGCGTTATTTATCGG<br>AGTTGCAGTTGCGCCCGCGAACGACATTTATAATGAACGTGAATTGCTCAACAGTATGAACATTTCGCA<br>GCCTACCGTAGTGTTTGTTTCCAAAAAGGGGTTGCAAAAAATTTTGAACGTGCAAAAAAAATTACCAAT<br>AATCCAGAAAATTATTATCATGGATTCTAAAACGGATTACCAGGGATTTCAGTCGATGTACACGTTCGT<br>CACATCTCATCTACCTCCCGGTTTTAATGAATACGATTTTGTACCAGAGTCCTTTGATCGTGACAAAAC<br>AATTGCACTGATAATGAATTCCTCTGGATCTACTGGGTTACCTAAGGGTGTGGCCCTTCCGCATAGAAC<br>TGCCTGCGTCAGATTCTCGCATGCCAGAGATCCTATTTTTGGCAATCAAATCATTCCGGATACTGCGAT<br>TTTAAGTGTTGTTCCATTCCATCACGGTTTTGGAATGTTTACTACACTCGGATATTTGATATGTGGATT<br>TCGAGTCGTCTTAATGTATAGATTTGAAGAAGAGCTGTTTTTACGATCCCTTCAGGATTACAAAATTCA<br>AAGTGCGTTGCTAGTACCAACCCTATTTTCATTCTTCGCCAAAAGCACTCTGATTGACAAATACGATTT<br>ATCTAATTTACACGAAATTGCTTCTGGGGGCGCACCTCTTTCGAAAGAAGTCGGGGAAGCGGTTGCAAA<br>ACGCTTCCATCTTCCAGGGATACGACAAGGATATGGGCTCACTGAGACTACATCAGCTATTCTGATTAC<br>ACCCGAGGGGGATGATAAACCGGGCGCGGTCGGTAAAGTTGTTCCATTTTTTGAAGCGAAGGTTGTGGA<br>TCTGGATACCGGGAAAACGCTGGGCGTTAATCAGAGAGGCGAATTATGTGTCAGAGGACCTATGATTAT<br>GTCCGGTTATGTAAACAATCCGGAAGCGACCAACGCCTTGATTGACAAGGATGGATGGCTACATTCTGG<br>AGACATAGCTTACTGGGACGAAGACGAACACTTCTTCATAGTTGACCGCTTGAAGTCTTTAATTAAATA<br>CAAAGGATATCAGGTGGCCCCCGCTGAATTGGAATCGATATTGTTACAACACCCCAACATCTTCGACGC<br>GGGCGTGGCAGGTCTTCCCGACGATGACGCCGGTGAACTTCCCGCCGCCGTTGTTGTTTTGGAGCACGG<br>AAAGACGATGACGGAAAAAGAGATCGTGGATTACGTGGCCAGTCAAGTAACAACCGCGAAAAAGTTGCG<br>CGGAGGAGTTGTGTTTGTGGACGAAGTACCGAAAGGTCTTACCGGAAAACTCGACGCAAGAAAAATCAG

	AGAGATCCTCATAAAGGCCAAGAAGGGCGGAAAGTCCAAATTGTAAGGATCCGGGCCCTATTCTATAGT<br>GTCACCTAAATGCTAGAGCTCGCTGATCAGCCTCGACTGTGCCTTCTAGTTGCCAGCCATCTGTTGTTT<br>GCCCCTCCCCCGTGCCTTCCTTGACCCTGGAAGGTGCCACTCCCACTGTCCTTTCCTAATAAAATGAGG<br>AAATTGCATCGCATTGTCTGAGTAGGTGTCATTCTATTCTTTTTTTTTTTTTTTTTTTTTTT<br>TTTTTTTTTTTTTTTTTTTTTTTTTTT
**Monocistronic Fluc G4mut 2445bp**	CGCCGTAATACGACTCACTATA**G**GGAGGCCG**A**G**A**CG**A**G**A**CTG**A**GAAGTAGTCG**A**GCG**A**GGTTGTGAGAC<br>GCCGCGCTCAGCTTCCATCGCTGGGCGGTCAACAAGTGCGGGCCTGGCTCAGCGCGGGGGGCGCGGAG<br>ACCGCGAGGCGACCGGGAGCGGCTGGGTTCCCGGCTGCGCGCCCTTCGGCCAGGCCGGGAGCCGCGCCA<br>GTCGGAGCCCCCGGCCCAGCGTGGTCCGCCTCCCTCTCGGCGTCCACCTGCCCGGAGTACTGCCAGCGG<br>GCATGACCGACCCACCAGGGGCGCCGCCGCCGGCGCTCGCAGGCCGCGGATGAAGAAGAAAACCCGGCG<br>CCGCTCGACCCGGAGCGAGGAGTTGACCCGGAGCGAGGAGTTGACCCTGAGTGAGGAAGCGACCTGGAG<br>TGAAGAGGCGACCCAGAGTGAGGAGGCGACCCAGGGCGAAGAGATGAATCGGAGCCAGGAGGTGACCCG<br>GGACGAGGAGTCGACCCGGAGCGAGGAGGTGACCAGGGAGGAAGTTAACATGGAAGACGCCAAAAACAT<br>AAAGAAAGGCCCGGCGCCATTCTATCCTCTAGAGGATGGAACCGCTGGAGAGCAACTGCATAAGGCTAT<br>GAAGAGATACGCCCTGGTTCCTGGAACAATTGCTTTTACAGATGCACATATCGAGGTGAACATCACGTA<br>CGCGGAATACTTCGAAATGTCCGTTCGGTTGGCAGAAGCTATGAAACGATATGGGCTGAATACAAATCA<br>CAGAATCGTCGTATGCAGTGAAAACTCTCTTCAATTCTTTATGCCGGTGTTGGGCGCGTTATTTATCGG<br>AGTTGCAGTTGCGCCCGCGAACGACATTTATAATGAACGTGAATTGCTCAACAGTATGAACATTTCGCA<br>GCCTACCGTAGTGTTTGTTTCCAAAAAGGGGTTGCAAAAAATTTTGAACGTGCAAAAAAAATTACCAAT<br>AATCCAGAAAATTATTATCATGGATTCTAAAACGGATTACCAGGGATTTCAGTCGATGTACACGTTCGT<br>CACATCTCATCTACCTCCCGGTTTTAATGAATACGATTTTGTACCAGAGTCCTTTGATCGTGACAAAAC<br>AATTGCACTGATAATGAATTCCTCTGGATCTACTGGGTTACCTAAGGGTGTGGCCCTTCCGCATAGAAC<br>TGCCTGCGTCAGATTCTCGCATGCCAGAGATCCTATTTTTGGCAATCAAATCATTCCGGATACTGCGAT<br>TTTAAGTGTTGTTCCATTCCATCACGGTTTTGGAATGTTTACTACACTCGGATATTTGATATGTGGATT<br>TCGAGTCGTCTTAATGTATAGATTTGAAGAAGAGCTGTTTTTACGATCCCTTCAGGATTACAAAATTCA<br>AAGTGCGTTGCTAGTACCAACCCTATTTTCATTCTTCGCCAAAAGCACTCTGATTGACAAATACGATTT<br>ATCTAATTTACACGAAATTGCTTCTGGGGGCGCACCTCTTTCGAAAGAAGTCGGGGAAGCGGTTGCAAA<br>ACGCTTCCATCTTCCAGGGATACGACAAGGATATGGGCTCACTGAGACTACATCAGCTATTCTGATTAC<br>ACCCGAGGGGGATGATAAACCGGGCGCGGTCGGTAAAGTTGTTCCATTTTTTGAAGCGAAGGTTGTGGA<br>TCTGGATACCGGGAAAACGCTGGGCGTTAATCAGAGAGGCGAATTATGTGTCAGAGGACCTATGATTAT<br>GTCCGGTTATGTAAACAATCCGGAAGCGACCAACGCCTTGATTGACAAGGATGGATGGCTACATTCTGG<br>AGACATAGCTTACTGGACGAAGACGAACACTTCTTCATAGTTGACCGCTTGAAGTCTTTAATTAAATA<br>CAAAGGATATCAGGTGGCCCCCGCTGAATTGGAATCGATATTGTTACAACACCCCAACATCTTCGACGC<br>GGGCGTGGCAGGTCTTCCCGACGATGACGCCGGTGAACTTCCCGCCGCCGTTGTTGTTTTGGAGCACGG

	AAAGACGATGACGGAAAAAGAGATCGTGGATTACGTGGCCAGTCAAGTAACAACCGCGAAAAAGTTGCG<br>CGGAGGAGTTGTGTTTGTGGACGAAGTACCGAAAGGTCTTACCGGAAAACTCGACGCAAGAAAAATCAG<br>AGAGATCCTCATAAAGGCCAAGAAGGGCGGAAAGTCCAAATTGTAAGGATCCGGGCCCTATTCTATAGT<br>GTCACCTAAATGCTAGAGCTCGCTGATCAGCCTCGACTGTGCCTTCTAGTTGCCAGCCATCTGTTGTTT<br>GCCCCTCCCCCGTGCCTTCCTTGACCCTGGAAGGTGCCACTCCCACTGTCCTTTCCTAATAAAATGAGG<br>AAATTGCATCGCATTGTCTGAGTAGGTGTCATTCTATTCTTTTTTTTTTTTTTTTTTTTT<br>TTTTTTTTTTTTTTTTTTTTTTTTTT
**Monocistronic Hairpin Fluc WT 2466bp**<br>Hairpin sequence	CGCCGTAATACGACTCACTATA**G**GGAGTGGACTTCGGTCCACTCCCAGGCCGGGGCGGGGCTGGAAGT<br>AGTCGGGCGGGGTTGTGAGACGCCGCGCTCAGCTTCCATCGCTGGGCGGTCAACAAGTGCGGGCCTGGC<br>TCAGCGCGGGGGGGCGCGGAGACCGCGAGGCGACCGGGAGCGGCTGGGTTCCCGGCTGCGCGCCCTTCG<br>GCCAGGCCGGGAGCCGCGCCAGTCGGAGCCCCCGGCCCAGCGTGGTCCGCCTCCCTCTCGGCGTCCACC<br>TGCCCGGAGTACTGCCAGCGGGCATGACCGACCCACCAGGGGCGCCGCCGCCGGCGCTCGCAGGCCGCG<br>GATGAAGAAGAAAACCCGGCGCCGCTCGACCCGGAGCGAGGAGTTGACCCGGAGCGAGGAGTTGACCCT<br>GAGTGAGGAAGCGACCTGGAGTGAAGAGGCGACCCAGAGTGAGGAGGCGACCCAGGGCGAAGAGATGAA<br>TCGGAGCCAGGAGGTGACCCGGGACGAGGAGTCGACCCGGAGCGAGGAGGTGACCAGGGAGGAAGTTAA<br>CATGGAAGACGCCAAAAACATAAAGAAAGGCCCGGCGCCATTCTATCCTCTAGAGGATGGAACCGCTGG<br>AGAGCAACTGCATAAGGCTATGAAGAGATACGCCCTGGTTCCTGGAACAATTGCTTTTACAGATGCACA<br>TATCGAGGTGAACATCACGTACGCGGAATACTTCGAAATGTCCGTTCGGTTGGCAGAAGCTATGAAACG<br>ATATGGGCTGAATACAAATCACAGAATCGTCGTATGCAGTGAAAACTCTCTTCAATTCTTTATGCCGGT<br>GTTGGGCGCGTTATTTATCGGAGTTGCAGTTGCGCCCGCGAACGACATTTATAATGAACGTGAATTGCT<br>CAACAGTATGAACATTTCGCAGCCTACCGTAGTGTTTGTTTCCAAAAAGGGGTTGCAAAAAATTTTGAA<br>CGTGCAAAAAAAATTACCAATAATCCAGAAAATTATTATCATGGATTCTAAAACGGATTACCAGGGATT<br>TCAGTCGATGTACACGTTCGTCACATCTCATCTACCTCCCGGTTTTAATGAATACGATTTTGTACCAGA<br>GTCCTTTGATCGTGACAAAACAATTGCACTGATAATGAATTCCTCTGGATCTACTGGGTTACCTAAGGG<br>TGTGGCCCTTCCGCATAGAACTGCCTGCGTCAGATTCTCGCATGCCAGAGATCCTATTTTTGGCAATCA<br>AATCATTCCGGATACTGCGATTTTAAGTGTTGTTCCATTCCATCACGGTTTTGGAATGTTTACTACACT<br>CGGATATTTGATATGTGGATTTCGAGTCGTCTTAATGTATAGATTTGAAGAAGAGCTGTTTTTACGATC<br>CCTTCAGGATTACAAAATTCAAAGTGCGTTGCTAGTACCAACCCTATTTTCATTCTTCGCCAAAAGCAC<br>TCTGATTGACAAATACGATTTATCTAATTTACACGAAATTGCTTCTGGGGGCGCACCTCTTTCGAAAGA<br>AGTCGGGGAAGCGGTTGCAAAACGCTTCCATCTTCCAGGGATACGACAAGGATATGGGCTCACTGAGAC<br>TACATCAGCTATTCTGATTACACCCGAGGGGGATGATAAACCGGGCGCGGTCGGTAAAGTTGTTCCATT<br>TTTTGAAGCGAAGGTTGTGGATCTGGATACCGGGAAAACGCTGGGCGTTAATCAGAGAGGCGAATTATG<br>TGTCAGAGGACCTATGATTATGTCCGGTTATGTAAACAATCCGGAAGCGACCAACGCCTTGATTGACAA<br>GGATGGATGGCTACATTCTGGAGACATAGCTTACTGGGACGAAGACGAACACTTCTTCATAGTTGACCG

	CTTGAAGTCTTTAATTAAATACAAAGGATATCAGGTGGCCCCCGCTGAATTGGAATCGATATTGTTACA<br>ACACCCCAACATCTTCGACGCGGGCGTGGCAGGTCTTCCCGACGATGACGCCGGTGAACTTCCCGCCGC<br>CGTTGTTGTTTTGGAGCACGGAAAGACGATGACGGAAAAAGAGATCGTGGATTACGTGGCCAGTCAAGT<br>AACAACCGCGAAAAAGTTGCGCGGAGGAGTTGTGTTTGTGGACGAAGTACCGAAAGGTCTTACCGGAAA<br>ACTCGACGCAAGAAAAATCAGAGAGATCCTCATAAAGGCCAAGAAGGGCGGAAAGTCCAAATTGTAAGG<br>ATCCGGGCCCTATTCTATAGTGTCACCTAAATGCTAGAGCTCGCTGATCAGCCTCGACTGTGCCTTCTA<br>GTTGCCAGCCATCTGTTGTTTGCCCCTCCCCCGTGCCTTCCTTGACCCTGGAAGGTGCCACTCCCACTG<br>TCCTTTCCTAATAAAATGAGGAAATTGCATCGCATTGTCTGAGTAGGTGTCATTCTATTCTTTTTTTT<br>TTTTTTTTTTTTTTTTTTTTTTTTTTTTTTTTTTTTTTTTTTTT
**Monocistronic Hairpin Fluc G4mut 2466bp**	CGCCGTAATACGACTCACTATA**G**GGAGTGGACTTCGGTCCACTCCCAGGCCG**A**G**A**CG**A**G**A**CTG**A**GAAGT<br>AGTCG**A**GCG**A**GGTTGTGAGACGCCGCGCTCAGCTTCCATCGCTGGGCGGTCAACAAGTGCGGGCCTGGC<br>TCAGCGCGGGGGGGCGCGGAGACCGCGAGGCGACCGGGAGCGGCTGGGTTCCCGGCTGCGCGCCCTTCG<br>GCCAGGCCGGGAGCCGCGCCAGTCGGAGCCCCCGGCCCAGCGTGGTCCGCCTCCCTCTCGGCGTCCACC<br>TGCCCGGAGTACTGCCAGCGGGCATGACCGACCCACCAGGGGCGCCGCCGCCGGCGCTCGCAGGCCGCG<br>GATGAAGAAGAAAACCCGGCGCCGCTCGACCCGGAGCGAGGAGTTGACCCGGAGCGAGGAGTTGACCCT<br>GAGTGAGGAAGCGACCTGGAGTGAAGAGGCGACCCAGAGTGAGGAGGCGACCCAGGGCGAAGAGATGAA<br>TCGGAGCCAGGAGGTGACCCGGGACGAGGAGTCGACCCGGAGCGAGGAGGTGACCAGGGAGGAAGTTAA<br>CATGGAAGACGCCAAAAACATAAAGAAAGGCCCGGCGCCATTCTATCCTCTAGAGGATGGAACCGCTGG<br>AGAGCAACTGCATAAGGCTATGAAGAGATACGCCCTGGTTCCTGGAACAATTGCTTTTACAGATGCACA<br>TATCGAGGTGAACATCACGTACGCGGAATACTTCGAAATGTCCGTTCGGTTGGCAGAAGCTATGAAACG<br>ATATGGGCTGAATACAAATCACAGAATCGTCGTATGCAGTGAAAACTCTCTTCAATTCTTTATGCCGGT<br>GTTGGGCGCGTTATTTATCGGAGTTGCAGTTGCGCCCGCGAACGACATTTATAATGAACGTGAATTGCT<br>CAACAGTATGAACATTTCGCAGCCTACCGTAGTGTTTGTTTCCAAAAAGGGGTTGCAAAAAATTTTGAA<br>CGTGCAAAAAAAATTACCAATAATCCAGAAAATTATTATCATGGATTCTAAAACGGATTACCAGGGATT<br>TCAGTCGATGTACACGTTCGTCACATCTCATCTACCTCCCGGTTTTAATGAATACGATTTTGTACCAGA<br>GTCCTTTGATCGTGACAAAACAATTGCACTGATAATGAATTCCTCTGGATCTACTGGGTTACCTAAGGG<br>TGTGGCCCTTCCGCATAGAACTGCCTGCGTCAGATTCTCGCATGCCAGAGATCCTATTTTTGGCAATCA<br>AATCATTCCGGATACTGCGATTTTAAGTGTTGTTCCATTCCATCACGGTTTTGGAATGTTTACTACACT<br>CGGATATTTGATATGTGGATTTCGAGTCGTCTTAATGTATAGATTTGAAGAAGAGCTGTTTTTACGATC<br>CCTTCAGGATTACAAAATTCAAAGTGCGTTGCTAGTACCAACCCTATTTTCATTCTTCGCCAAAAGCAC<br>TCTGATTGACAAATACGATTTATCTAATTTACACGAAATTGCTTCTGGGGGCGCACCTCTTTCGAAAGA<br>AGTCGGGGAAGCGGTTGCAAAACGCTTCCATCTTCCAGGGATACGACAAGGATATGGGCTCACTGAGAC<br>TACATCAGCTATTCTGATTACACCCGAGGGGGATGATAAACCGGGCGCGGTCGGTAAAGTTGTTCCATT<br>TTTTGAAGCGAAGGTTGTGGATCTGGATACCGGGAAAACGCTGGGCGTTAATCAGAGAGGCGAATTATG

	TGTCAGAGGACCTATGATTATGTCCGGTTATGTAAACAATCCGGAAGCGACCAACGCCTTGATTGACAA<br>GGATGGATGGCTACATTCTGGAGACATAGCTTACTGGGACGAAGACGAACACTTCTTCATAGTTGACCG<br>CTTGAAGTCTTTAATTAAATACAAAGGATATCAGGTGGCCCCCGCTGAATTGGAATCGATATTGTTACA<br>ACACCCCAACATCTTCGACGCGGGCGTGGCAGGTCTTCCCGACGATGACGCCGGTGAACTTCCCGCCGC<br>CGTTGTTGTTTTGGAGCACGGAAAGACGATGACGGAAAAAGAGATCGTGGATTACGTGGCCAGTCAAGT<br>AACAACCGCGAAAAAGTTGCGCGGAGGAGTTGTGTTTGTGGACGAAGTACCGAAAGGTCTTACCGGAAA<br>ACTCGACGCAAGAAAAATCAGAGAGATCCTCATAAAGGCCAAGAAGGGCGGAAAGTCCAAATTGTAAGG<br>ATCCGGGCCCTATTCTATAGTGTCACCTAAATGCTAGAGCTCGCTGATCAGCCTCGACTGTGCCTTCTA<br>GTTGCCAGCCATCTGTTGTTTGCCCCTCCCCCGTGCCTTCCTTGACCCTGGAAGGTGCCACTCCCACTG<br>TCCTTTCCTAATAAAATGAGGAAATTGCATCGCATTGTCTGAGTAGGTGTCATTCTATTCTTTTTTTTTT<br>TTTTTTTTTTTTTTTTTTTTTTTTTTTTTTTTTTTTTTTTTTTTTTTTTTTT
**Bicistronic WT 3479bp**	CGCCGTAATACGACTCACTATA**G**GGCTAGCCACCATGACTTCGAAAGTTTATGATCCAGAACAAAGGAA<br>ACGGATGATAACTGGTCCGCAGTGGTGGGCCAGATGTAAACAAATGAATGTTCTTGATTCATTTATTAA<br>TTATTATGATTCAGAAAAACATGCAGAAAATGCTGTTATTTTTTTACATGGTAACGCGGCCTCTTCTTA<br>TTTATGGCGACATGTTGTGCCACATATTGAGCCAGTAGCGCGGTGTATTATACCAGACCTTATTGGTAT<br>GGGCAAATCAGGCAAATCTGGTAATGGTTCTTATAGGTTACTTGATCATTACAAATATCTTACTGCATG<br>GTTTGAACTTCTTAATTTACCAAAGAAGATCATTTTTGTCGGCCATGATTGGGGTGCTTGTTTGGCATT<br>TCATTATAGCTATGAGCATCAAGATAAGATCAAAGCAATAGTTCACGCTGAAAGTGTAGTAGATGTGAT<br>TGAATCATGGGATGAATGGCCTGATATTGAAGAAGATATTGCGTTGATCAAATCTGAAGAAGGAGAAAA<br>AATGGTTTTGGAGAATAACTTCTTCGTGGAAACCATGTTGCCATCAAAAATCATGAGAAAGTTAGAACC<br>AGAAGAATTTGCAGCATATCTTGAACCATTCAAAGAGAAAGGTGAAGTTCGTCGTCCAACATTATCATG<br>GCCTCGTGAAATCCCGTTAGTAAAAGGTGGTAAACCTGACGTTGTACAAATTGTTAGGAATTATAATGC<br>TTATCTACGTGCAAGTGATGATTTACCAAAAATGTTTATTGAATCGGACCCAGGATTCTTTTCCAATGC<br>TATTGTTGAAGGTGCCAAGAAGTTTCCTAATACTGAATTTGTCAAAGTAAAAGGTCTTCATTTTTCGCA<br>AGAAGATGCACCTGATGAAATGGGAAAATATATCAAATCGTTCGTTGAGCGAGTTCTCAAAAATGAACA<br>ATAATTCTAGAAATGTAACTGTATTCAGCGATGACGAAATTCTTAGCTATTGTAATACTCTAGAGGATC<br>CCCCGGGCGAGCTCCCGCGGCCGCAGGCCGGGGCGGGGCTGGAAGTAGTCGGCGGGGTTGTGAGACG<br>CCGCGCTCAGCTTCCATCGCTGGGCGGTCAACAAGTGCGGGCCTGGCTCAGCGCGGGGGGGCGCGGAGA<br>CCGCGAGGCGACCGGGAGCGGCTGGGTTCCCGGCTGCGCGCCCTTCGGCCAGGCCGGGAGCCGCGCCAG<br>TCGGAGCCCCCGGCCCAGCGTGGTCCGCCTCCCTCTCGGCGTCCACCTGCCCGGAGTACTGCCAGCGGG<br>CATGACCGACCCACCAGGGGCGCCGCCGCCGGCGCTCGCAGGCCGCGGATGAAGAAGAAAACCCGGCGC<br>CGCTCGACCCGGAGCGAGGAGTTGACCCGGAGCGAGGAGTTGACCCTGAGTGAGGAAGCGACCTGGAGT<br>GAAGAGGCGACCCAGAGTGAGGAGGCGACCCAGGGCGAAGAGATGAATCGGAGCCAGGAGGTGACCCGG<br>GACGAGGAGTCGACCCGGAGCGAGGAGGTGACCAGGGAGGAAGTTAACATGGAAGACGCCAAAAACATA

	AAGAAAGGCCCGGCGCCATTCTATCCTCTAGAGGATGGAACCGCTGGAGAGCAACTGCATAAGGCTATG<br>AAGAGATACGCCCTGGTTCCTGGAACAATTGCTTTTACAGATGCACATATCGAGGTGAACATCACGTAC<br>GCGGAATACTTCGAAATGTCCGTTCGGTTGGCAGAAGCTATGAAACGATATGGGCTGAATACAAATCAC<br>AGAATCGTCGTATGCAGTGAAAACTCTCTTCAATTCTTTATGCCGGTGTTGGGCGCGTTATTTATCGGA<br>GTTGCAGTTGCGCCCGCGAACGACATTTATAATGAACGTGAATTGCTCAACAGTATGAACATTTCGCAG<br>CCTACCGTAGTGTTTGTTTCCAAAAAGGGGTTGCAAAAAATTTTGAACGTGCAAAAAAAATTACCAATA<br>ATCCAGAAAATTATTATCATGGATTCTAAAACGGATTACCAGGGATTTCAGTCGATGTACACGTTCGTC<br>ACATCTCATCTACCTCCCGGTTTTAATGAATACGATTTTGTACCAGAGTCCTTTGATCGTGACAAAACA<br>ATTGCACTGATAATGAATTCCTCTGGATCTACTGGGTTACCTAAGGGTGTGGCCCTTCCGCATAGAACT<br>GCCTGCGTCAGATTCTCGCATGCCAGAGATCCTATTTTTGGCAATCAAATCATTCCGGATACTGCGATT<br>TTAAGTGTTGTTCCATTCCATCACGGTTTTGGAATGTTTACTACACTCGGATATTTGATATGTGGATTT<br>CGAGTCGTCTTAATGTATAGATTTGAAGAAGAGCTGTTTTTACGATCCCTTCAGGATTACAAAATTCAA<br>AGTGCGTTGCTAGTACCAACCCTATTTTCATTCTTCGCCAAAAGCACTCTGATTGACAAATACGATTTA<br>TCTAATTTACACGAAATTGCTTCTGGGGGCGCACCTCTTTCGAAAGAAGTCGGGGAAGCGGTTGCAAAA<br>CGCTTCCATCTTCCAGGGATACGACAAGGATATGGGCTCACTGAGACTACATCAGCTATTCTGATTACA<br>CCCGAGGGGGATGATAAACCGGGCGCGGTCGGTAAAGTTGTTCCATTTTTTGAAGCGAAGGTTGTGGAT<br>CTGGATACCGGGAAAACGCTGGGCGTTAATCAGAGAGGCGAATTATGTGTCAGAGGACCTATGATTATG<br>TCCGGTTATGTAAACAATCCGGAAGCGACCAACGCCTTGATTGACAAGGATGGATGGCTACATTCTGGA<br>GACATAGCTTACTGGGACGAAGACGAACACTTCTTCATAGTTGACCGCTTGAAGTCTTTAATTAAATAC<br>AAAGGATATCAGGTGGCCCCCGCTGAATTGGAATCGATATTGTTACAACACCCCAACATCTTCGACGCG<br>GGCGTGGCAGGTCTTCCCGACGATGACGCCGGTGAACTTCCCGCCGCCGTTGTTGTTTTGGAGCACGGA<br>AAGACGATGACGGAAAAAGAGATCGTGGATTACGTGGCCAGTCAAGTAACAACCGCGAAAAAGTTGCGC<br>GGAGGAGTTGTGTTTGTGGACGAAGTACCGAAAGGTCTTACCGGAAAACTCGACGCAAGAAAAATCAGA<br>GAGATCCTCATAAAGGCCAAGAAGGGCGGAAAGTCCAAATTGTAAGGATCCGGGCCCTATTCTATAGTG<br>TCACCTAAATGCTAGAGCTCGCTGATCAGCCTCGACTGTGCCTTCTAGTTGCCAGCCATCTGTTGTTTG<br>CCCCTCCCCCGTGCCTTCCTTGACCCTGGAAGGTGCCACTCCCACTGTCCTTTCCTAATAAAATGAGGA<br>AATTGCATCGCATTGTCTGAGTAGGTGTCATTCTATTCTTTTTTTTTTTTTTTTTTTTTTTTTTTTTTTT<br>TTTTTTTTTTTTTTTTTTTTTTTTTTTTT
**Bicistronic G4mut 3479bp**	CGCCGTAATACGACTCACTATA**G**GGCTAGCCACCATGACTTCGAAAGTTTATGATCCAGAACAAAGGAA<br>ACGGATGATAACTGGTCCGCAGTGGTGGGCCAGATGTAAACAAATGAATGTTCTTGATTCATTTATTAA<br>TTATTATGATTCAGAAAAACATGCAGAAAATGCTGTTATTTTTTTACATGGTAACGCGGCCTCTTCTTA<br>TTTATGGCGACATGTTGTGCCACATATTGAGCCAGTAGCGCGGTGTATTATACCAGACCTTATTGGTAT<br>GGGCAAATCAGGCAAATCTGGTAATGGTTCTTATAGGTTACTTGATCATTACAAATATCTTACTGCATG<br>GTTTGAACTTCTTAATTTACCAAAGAAGATCATTTTTGTCGGCCATGATTGGGGTGCTTGTTTGGCATT

	TCATTATAGCTATGAGCATCAAGATAAGATCAAAGCAATAGTTCACGCTGAAAGTGTAGTAGATGTGAT<br>TGAATCATGGGATGAATGGCCTGATATTGAAGAAGATATTGCGTTGATCAAATCTGAAGAAGGAGAAAA<br>AATGGTTTTGGAGAATAACTTCTTCGTGGAAACCATGTTGCCATCAAAAATCATGAGAAAGTTAGAACC<br>AGAAGAATTTGCAGCATATCTTGAACCATTCAAAGAGAAAGGTGAAGTTCGTCGTCCAACATTATCATG<br>GCCTCGTGAAATCCCGTTAGTAAAAGGTGGTAAACCTGACGTTGTACAAATTGTTAGGAATTATAATGC<br>TTATCTACGTGCAAGTGATGATTTACCAAAAATGTTTATTGAATCGGACCCAGGATTCTTTTCCAATGC<br>TATTGTTGAAGGTGCCAAGAAGTTTCCTAATACTGAATTTGTCAAAGTAAAAGGTCTTCATTTTTCGCA<br>AGAAGATGCACCTGATGAAATGGGAAAATATATCAAATCGTTCGTTGAGCGAGTTCTCAAAAATGAACA<br>ATAATTCTAGAAATGTAACTGTATTCAGCGATGACGAAATTCTTAGCTATTGTAATACTCTAGAGGATC<br>CCCCGGGCGAGCTCCCGCGGCCGCAGGCCG**A**G**A**CG**A**G**A**CTG**A**GAAGTAGTCG**A**GCG**A**GGTTGTGAGACG<br>CCGCGCTCAGCTTCCATCGCTGGGCGGTCAACAAGTGCGGGCCTGGCTCAGCGCGGGGGGGCGCGGAGA<br>CCGCGAGGCGACCGGGAGCGGCTGGGTTCCCGGCTGCGCGCCCTTCGGCCAGGCCGGGAGCCGCGCCAG<br>TCGGAGCCCCCGGCCCAGCGTGGTCCGCCTCCCTCTCGGCGTCCACCTGCCCGGAGTACTGCCAGCGGG<br>CATGACCGACCCACCAGGGGCGCCGCCGCCGGCGCTCGCAGGCCGCGGATGAAGAAGAAAACCCGGCGC<br>CGCTCGACCCGGAGCGAGGAGTTGACCCGGAGCGAGGAGTTGACCCTGAGTGAGGAAGCGACCTGGAGT<br>GAAGAGGCGACCCAGAGTGAGGAGGCGACCCAGGGCGAAGAGATGAATCGGAGCCAGGAGGTGACCCGG<br>GACGAGGAGTCGACCCGGAGCGAGGAGGTGACCAGGGAGGAAGTTAACATGGAAGACGCCAAAAACATA<br>AAGAAAGGCCCGGCGCCATTCTATCCTCTAGAGGATGGAACCGCTGGAGAGCAACTGCATAAGGCTATG<br>AAGAGATACGCCCTGGTTCCTGGAACAATTGCTTTTACAGATGCACATATCGAGGTGAACATCACGTAC<br>GCGGAATACTTCGAAATGTCCGTTCGGTTGGCAGAAGCTATGAAACGATATGGGCTGAATACAAATCAC<br>AGAATCGTCGTATGCAGTGAAAACTCTCTTCAATTCTTTATGCCGGTGTTGGGCGCGTTATTTATCGGA<br>GTTGCAGTTGCGCCCGCGAACGACATTTATAATGAACGTGAATTGCTCAACAGTATGAACATTTCGCAG<br>CCTACCGTAGTGTTTGTTTCCAAAAAGGGGTTGCAAAAAATTTTGAACGTGCAAAAAAAATTACCAATA<br>ATCCAGAAAATTATTATCATGGATTCTAAAACGGATTACCAGGGATTTCAGTCGATGTACACGTTCGTC<br>ACATCTCATCTACCTCCCGGTTTTAATGAATACGATTTTGTACCAGAGTCCTTTGATCGTGACAAAACA<br>ATTGCACTGATAATGAATTCCTCTGGATCTACTGGGTTACCTAAGGGTGTGGCCCTTCCGCATAGAACT<br>GCCTGCGTCAGATTCTCGCATGCCAGAGATCCTATTTTTGGCAATCAAATCATTCCGGATACTGCGATT<br>TTAAGTGTTGTTCCATTCCATCACGGTTTTGGAATGTTTACTACACTCGGATATTTGATATGTGGATTT<br>CGAGTCGTCTTAATGTATAGATTTGAAGAAGAGCTGTTTTTACGATCCCTTCAGGATTACAAAATTCAA<br>AGTGCGTTGCTAGTACCAACCCTATTTTCATTCTTCGCCAAAAGCACTCTGATTGACAAATACGATTTA<br>TCTAATTTACACGAAATTGCTTCTGGGGGCGCACCTCTTTCGAAAGAAGTCGGGGAAGCGGTTGCAAAA<br>CGCTTCCATCTTCCAGGGATACGACAAGGATATGGGCTCACTGAGACTACATCAGCTATTCTGATTACA<br>CCCGAGGGGGATGATAAACCGGGCGCGGTCGGTAAAGTTGTTCCATTTTTTGAAGCGAAGGTTGTGGAT<br>CTGGATACCGGGAAAACGCTGGGCGTTAATCAGAGAGGCGAATTATGTGTCAGAGGACCTATGATTATG

	TCCGGTTATGTAAACAATCCGGAAGCGACCAACGCCTTGATTGACAAGGATGGATGGCTACATTCTGGA<br>GACATAGCTTACTGGGACGAAGACGAACACTTCTTCATAGTTGACCGCTTGAAGTCTTTAATTAAATAC<br>AAAGGATATCAGGTGGCCCCCGCTGAATTGGAATCGATATTGTTACAACACCCCAACATCTTCGACGCG<br>GGCGTGGCAGGTCTTCCCGACGATGACGCCGGTGAACTTCCCGCCGCCGTTGTTGTTTTGGAGCACGGA<br>AAGACGATGACGGAAAAAGAGATCGTGGATTACGTGGCCAGTCAAGTAACAACCGCGAAAAAGTTGCGC<br>GGAGGAGTTGTGTTTGTGGACGAAGTACCGAAAGGTCTTACCGGAAAACTCGACGCAAGAAAAATCAGA<br>GAGATCCTCATAAAGGCCAAGAAGGGCGGAAAGTCCAAATTGTAAGGATCCGGGCCCTATTCTATAGTG<br>TCACCTAAATGCTAGAGCTCGCTGATCAGCCTCGACTGTGCCTTCTAGTTGCCAGCCATCTGTTGTTTG<br>CCCCTCCCCCGTGCCTTCCTTGACCCTGGAAGGTGCCACTCCCACTGTCCTTTCCTAATAAAATGAGGA<br>AATTGCATCGCATTGTCTGAGTAGGTGTCATTCTATTCTTTTTTTTTTTTTTTTTTTTTT<br>TTTTTTTTTTTTTTTTTTTTTTTTT
**Bicistronic Hairpin WT 3500pb**	CGCCGTAATACGACTCACTATA**G**GGAGTGGACTTCGGTCCACTCCCCTAGCCACCATGACTTCGAAAGT<br>TTATGATCCAGAACAAAGGAAACGGATGATAACTGGTCCGCAGTGGTGGGCCAGATGTAAACAAATGAA<br>TGTTCTTGATTCATTTATTAATTATTATGATTCAGAAAAACATGCAGAAAATGCTGTTATTTTTTTACA<br>TGGTAACGCGGCCTCTTCTTATTTATGGCGACATGTTGTGCCACATATTGAGCCAGTAGCGCGGTGTAT<br>TATACCAGACCTTATTGGTATGGGCAAATCAGGCAAATCTGGTAATGGTTCTTATAGGTTACTTGATCA<br>TTACAAATATCTTACTGCATGGTTTGAACTTCTTAATTTACCAAAGAAGATCATTTTTGTCGGCCATGA<br>TTGGGGTGCTTGTTTGGCATTTCATTATAGCTATGAGCATCAAGATAAGATCAAAGCAATAGTTCACGC<br>TGAAAGTGTAGTAGATGTGATTGAATCATGGGATGAATGGCCTGATATTGAAGAAGATATTGCGTTGAT<br>CAAATCTGAAGAAGGAGAAAAAATGGTTTTGGAGAATAACTTCTTCGTGGAAACCATGTTGCCATCAAA<br>AATCATGAGAAAGTTAGAACCAGAAGAATTTGCAGCATATCTTGAACCATTCAAAGAGAAAGGTGAAGT<br>TCGTCGTCCAACATTATCATGGCCTCGTGAAATCCCGTTAGTAAAAGGTGGTAAACCTGACGTTGTACA<br>AATTGTTAGGAATTATAATGCTTATCTACGTGCAAGTGATGATTTACCAAAAATGTTTATTGAATCGGA<br>CCCAGGATTCTTTTCCAATGCTATTGTTGAAGGTGCCAAGAAGTTTCCTAATACTGAATTTGTCAAAGT<br>AAAAGGTCTTCATTTTTCGCAAGAAGATGCACCTGATGAAATGGGAAAATATATCAAATCGTTCGTTGA<br>GCGAGTTCTCAAAAATGAACAATAATTCTAGAAATGTAACTGTATTCAGCGATGACGAAATTCTTAGCT<br>ATTGTAATACTCTAGAGGATCCCCCGGGCGAGCTCCCGCGGCCGCAGGCCGGGGCGGGGCTGGAAGTA<br>GTCGGGCGGGGTTGTGAGACGCCGCGCTCAGCTTCCATCGCTGGGCGGTCAACAAGTGCGGGCCTGGCT<br>CAGCGCGGGGGGGCGCGGAGACCGCGAGGCGACCGGGAGCGGCTGGGTTCCCGGCTGCGCGCCCTTCGG<br>CCAGGCCGGGAGCCGCGCCAGTCGGAGCCCCCGGCCCAGCGTGGTCCGCCTCCCTCTCGGCGTCCACCT<br>GCCCGGAGTACTGCCAGCGGGCATGACCGACCCACCAGGGGCGCCGCCGCCGGCGCTCGCAGGCCGCGG<br>ATGAAGAAGAAAACCCGGCGCCGCTCGACCCGGAGCGAGGAGTTGACCCGGAGCGAGGAGTTGACCCTG<br>AGTGAGGAAGCGACCTGGAGTGAAGAGGCGACCCAGAGTGAGGAGGCGACCCAGGGCGAAGAGATGAAT<br>CGGAGCCAGGAGGTGACCCGGGACGAGGAGTCGACCCGGAGCGAGGAGGTGACCAGGGAGGAAGTTAAC

	ATGGAAGACGCCAAAAACATAAAGAAAGGCCCGGCGCCATTCTATCCTCTAGAGGATGGAACCGCTGGA<br>GAGCAACTGCATAAGGCTATGAAGAGATACGCCCTGGTTCCTGGAACAATTGCTTTTACAGATGCACAT<br>ATCGAGGTGAACATCACGTACGCGGAATACTTCGAAATGTCCGTTCGGTTGGCAGAAGCTATGAAACGA<br>TATGGGCTGAATACAAATCACAGAATCGTCGTATGCAGTGAAAACTCTCTTCAATTCTTTATGCCGGTG<br>TTGGGCGCGTTATTTATCGGAGTTGCAGTTGCGCCCGCGAACGACATTTATAATGAACGTGAATTGCTC<br>AACAGTATGAACATTTCGCAGCCTACCGTAGTGTTTGTTTCCAAAAAGGGGTTGCAAAAAATTTTGAAC<br>GTGCAAAAAAAATTACCAATAATCCAGAAAATTATTATCATGGATTCTAAAACGGATTACCAGGGATTT<br>CAGTCGATGTACACGTTCGTCACATCTCATCTACCTCCCGGTTTTAATGAATACGATTTTGTACCAGAG<br>TCCTTTGATCGTGACAAAACAATTGCACTGATAATGAATTCCTCTGGATCTACTGGGTTACCTAAGGGT<br>GTGGCCCTTCCGCATAGAACTGCCTGCGTCAGATTCTCGCATGCCAGAGATCCTATTTTTGGCAATCAA<br>ATCATTCCGGATACTGCGATTTTAAGTGTTGTTCCATTCCATCACGGTTTTGGAATGTTTACTACACTC<br>GGATATTTGATATGTGGATTTCGAGTCGTCTTAATGTATAGATTTGAAGAAGAGCTGTTTTTACGATCC<br>CTTCAGGATTACAAAATTCAAAGTGCGTTGCTAGTACCAACCCTATTTTCATTCTTCGCCAAAAGCACT<br>CTGATTGACAAATACGATTTATCTAATTTACACGAAATTGCTTCTGGGGGCGCACCTCTTTCGAAAGAA<br>GTCGGGGAAGCGGTTGCAAAACGCTTCCATCTTCCAGGGATACGACAAGGATATGGGCTCACTGAGACT<br>ACATCAGCTATTCTGATTACACCCGAGGGGGATGATAAACCGGGCGCGGTCGGTAAAGTTGTTCCATTT<br>TTTGAAGCGAAGGTTGTGGATCTGGATACCGGGAAAACGCTGGGCGTTAATCAGAGAGGCGAATTATGT<br>GTCAGAGGACCTATGATTATGTCCGGTTATGTAAACAATCCGGAAGCGACCAACGCCTTGATTGACAAG<br>GATGGATGGCTACATTCTGGAGACATAGCTTACTGGGACGAAGACGAACACTTCTTCATAGTTGACCGC<br>TTGAAGTCTTTAATTAAATACAAAGGATATCAGGTGGCCCCCGCTGAATTGGAATCGATATTGTTACAA<br>CACCCCAACATCTTCGACGCGGGCGTGGCAGGTCTTCCCGACGATGACGCCGGTGAACTTCCCGCCGCC<br>GTTGTTGTTTTGGAGCACGGAAAGACGATGACGGAAAAAGAGATCGTGGATTACGTGGCCAGTCAAGTA<br>ACAACCGCGAAAAAGTTGCGCGGAGGAGTTGTGTTTGTGGACGAAGTACCGAAAGGTCTTACCGGAAAA<br>CTCGACGCAAGAAAAATCAGAGAGATCCTCATAAAGGCCAAGAAGGGCGGAAAGTCCAAATTGTAAGGA<br>TCCGGGCCCTATTCTATAGTGTCACCTAAATGCTAGAGCTCGCTGATCAGCCTCGACTGTGCCTTCTAG<br>TTGCCAGCCATCTGTTGTTTGCCCCTCCCCCGTGCCTTCCTTGACCCTGGAAGGTGCCACTCCCACTGT<br>CCTTTCCTAATAAAATGAGGAAATTGCATCGCATTGTCTGAGTAGGTGTCATTCTATTCTTTTTTTTTT<br>TTTTTTTTTTTTTTTTTTTTTTTTTTTTTTTTTTTTTTTTTTTTTTTTTTT
**BicistronicHairpin G4mut 3500pb**	CGCCGTAATACGACTCACTATA**G**GGAGTGGACTTCGGTCCACTCCCCTAGCCACCATGACTTCGAAAGT<br>TTATGATCCAGAACAAAGGAAACGGATGATAACTGGTCCGCAGTGGTGGGCCAGATGTAAACAAATGAA<br>TGTTCTTGATTCATTTATTAATTATTATGATTCAGAAAAACATGCAGAAAATGCTGTTATTTTTTTACA<br>TGGTAACGCGGCCTCTTCTTATTTATGGCGACATGTTGTGCCACATATTGAGCCAGTAGCGCGGTGTAT<br>TATACCAGACCTTATTGGTATGGGCAAATCAGGCAAATCTGGTAATGGTTCTTATAGGTTACTTGATCA<br>TTACAAATATCTTACTGCATGGTTTGAACTTCTTAATTTACCAAAGAAGATCATTTTTGTCGGCCATGA

	TTGGGGTGCTTGTTTGGCATTTCATTATAGCTATGAGCATCAAGATAAGATCAAAGCAATAGTTCACGC<br>TGAAAGTGTAGTAGATGTGATTGAATCATGGGATGAATGGCCTGATATTGAAGAAGATATTGCGTTGAT<br>CAAATCTGAAGAAGGAGAAAAAATGGTTTTGGAGAATAACTTCTTCGTGGAAACCATGTTGCCATCAAA<br>AATCATGAGAAAGTTAGAACCAGAAGAATTTGCAGCATATCTTGAACCATTCAAAGAGAAAGGTGAAGT<br>TCGTCGTCCAACATTATCATGGCCTCGTGAAATCCCGTTAGTAAAAGGTGGTAAACCTGACGTTGTACA<br>AATTGTTAGGAATTATAATGCTTATCTACGTGCAAGTGATGATTTACCAAAAATGTTTATTGAATCGGA<br>CCCAGGATTCTTTTCCAATGCTATTGTTGAAGGTGCCAAGAAGTTTCCTAATACTGAATTTGTCAAAGT<br>AAAAGGTCTTCATTTTTCGCAAGAAGATGCACCTGATGAAATGGGAAAATATATCAAATCGTTCGTTGA<br>GCGAGTTCTCAAAAATGAACAATAATTCTAGAAATGTAACTGTATTCAGCGATGACGAAATTCTTAGCT<br>ATTGTAATACTCTAGAGGATCCCCCGGGCGAGCTCCCGCGGCCGCAGGCCG**A**G**A**CG**A**G**A**CTG**A**GAAGTA<br>GTCG**A**GCG**A**GGTTGTGAGACGCCGCGCTCAGCTTCCATCGCTGGGCGGTCAACAAGTGCGGGCCTGGCT<br>CAGCGCGGGGGGGCGCGGAGACCGCGAGGCGACCGGGAGCGGCTGGGTTCCCGGCTGCGCGCCCTTCGG<br>CCAGGCCGGGAGCCGCGCCAGTCGGAGCCCCCGGCCCAGCGTGGTCCGCCTCCCTCTCGGCGTCCACCT<br>GCCCGGAGTACTGCCAGCGGGCATGACCGACCCACCAGGGGCGCCGCCGCCGGCGCTCGCAGGCCGCGG<br>ATGAAGAAGAAAACCCGGCGCCGCTCGACCCGGAGCGAGGAGTTGACCCGGAGCGAGGAGTTGACCCTG<br>AGTGAGGAAGCGACCTGGAGTGAAGAGGCGACCCAGAGTGAGGAGGCGACCCAGGGCGAAGAGATGAAT<br>CGGAGCCAGGAGGTGACCCGGGACGAGGAGTCGACCCGGAGCGAGGAGGTGACCAGGGAGGAAGTTAAC<br>ATGGAAGACGCCAAAAACATAAAGAAAGGCCCGGCGCCATTCTATCCTCTAGAGGATGGAACCGCTGGA<br>GAGCAACTGCATAAGGCTATGAAGAGATACGCCCTGGTTCCTGGAACAATTGCTTTTACAGATGCACAT<br>ATCGAGGTGAACATCACGTACGCGGAATACTTCGAAATGTCCGTTCGGTTGGCAGAAGCTATGAAACGA<br>TATGGGCTGAATACAAATCACAGAATCGTCGTATGCAGTGAAAACTCTCTTCAATTCTTTATGCCGGTG<br>TTGGGCGCGTTATTTATCGGAGTTGCAGTTGCGCCCGCGAACGACATTTATAATGAACGTGAATTGCTC<br>AACAGTATGAACATTTCGCAGCCTACCGTAGTGTTTGTTTCCAAAAAGGGGTTGCAAAAAATTTTGAAC<br>GTGCAAAAAAAATTACCAATAATCCAGAAAATTATTATCATGGATTCTAAAACGGATTACCAGGGATTT<br>CAGTCGATGTACACGTTCGTCACATCTCATCTACCTCCCGGTTTTAATGAATACGATTTTGTACCAGAG<br>TCCTTTGATCGTGACAAAACAATTGCACTGATAATGAATTCCTCTGGATCTACTGGGTTACCTAAGGGT<br>GTGGCCCTTCCGCATAGAACTGCCTGCGTCAGATTCTCGCATGCCAGAGATCCTATTTTTGGCAATCAA<br>ATCATTCCGGATACTGCGATTTTAAGTGTTGTTCCATTCCATCACGGTTTTGGAATGTTTACTACACTC<br>GGATATTTGATATGTGGATTTCGAGTCGTCTTAATGTATAGATTTGAAGAAGAGCTGTTTTTACGATCC<br>CTTCAGGATTACAAAATTCAAAGTGCGTTGCTAGTACCAACCCTATTTTCATTCTTCGCCAAAAGCACT<br>CTGATTGACAAATACGATTTATCTAATTTACACGAAATTGCTTCTGGGGGCGCACCTCTTTCGAAAGAA<br>GTCGGGGAAGCGGTTGCAAAACGCTTCCATCTTCCAGGGATACGACAAGGATATGGGCTCACTGAGACT<br>ACATCAGCTATTCTGATTACACCCGAGGGGGATGATAAACCGGGCGCGGTCGGTAAAGTTGTTCCATTT<br>TTTGAAGCGAAGGTTGTGGATCTGGATACCGGGAAAACGCTGGGCGTTAATCAGAGAGGCGAATTATGT

	GTCAGAGGACCTATGATTATGTCCGGTTATGTAAACAATCCGGAAGCGACCAACGCCTTGATTGACAAG<br>GATGGATGGCTACATTCTGGAGACATAGCTTACTGGGACGAAGACGAACACTTCTTCATAGTTGACCGC<br>TTGAAGTCTTTAATTAAATACAAAGGATATCAGGTGGCCCCCGCTGAATTGGAATCGATATTGTTACAA<br>CACCCCAACATCTTCGACGCGGGCGTGGCAGGTCTTCCCGACGATGACGCCGGTGAACTTCCCGCCGCC<br>GTTGTTGTTTTGGAGCACGGAAAGACGATGACGGAAAAAGAGATCGTGGATTACGTGGCCAGTCAAGTA<br>ACAACCGCGAAAAAGTTGCGCGGAGGAGTTGTGTTTGTGGACGAAGTACCGAAAGGTCTTACCGGAAAA<br>CTCGACGCAAGAAAAATCAGAGAGATCCTCATAAAGGCCAAGAAGGGCGGAAAGTCCAAATTGTAAGGA<br>TCCGGGCCCTATTCTATAGTGTCACCTAAATGCTAGAGCTCGCTGATCAGCCTCGACTGTGCCTTCTAG<br>TTGCCAGCCATCTGTTGTTTGCCCCTCCCCCGTGCCTTCCTTGACCCTGGAAGGTGCCACTCCCACTGT<br>CCTTTCCTAATAAAATGAGGAAATTGCATCGCATTGTCTGAGTAGGTGTCATTCTATTCTTTTTTTTTT<br>TTTTTTTTTTTTTTTTTTTTTTTTTTTTTTTTTTTTTTTTTTTTTTTTTT

Supplementary References

1. Noderer,W.L., Flockhart,R.J., Bhaduri,A., Arce,A.J.D. de, Zhang,J., Khavari,P.A. and Wang,C.L. (2014) Quantitative analysis of mammalian translation initiation sites by FACS- seq. *Mol. Syst. Biol.*, **10**, 748.

2. Diaz de Arce,A.J., Noderer,W.L. and Wang,C.L. (2018) Complete motif analysis of sequence requirements for translation initiation at non-AUG start codons. *Nucleic Acids Res.*, **46**, 985–994.

3. Kozak,M. (1987) At least six nucleotides preceding the AUG initiator codon enhance translation in mammalian cells. *J. Mol. Biol.*, **196**, 947–950.
